# Chronic alcohol metabolism results in DNA repair infidelity and cell cycle‐induced senescence in neurons

**DOI:** 10.1111/acel.13772

**Published:** 2023-01-23

**Authors:** Jacquelyne Ka‐Li Sun, Deng Wu, Genper Chi‐Ngai Wong, Tsun‐Ming Lau, Meigui Yang, Ronald P. Hart, Kin‐Ming Kwan, Ho Yin Edwin Chan, Hei‐Man Chow

**Affiliations:** ^1^ School of Life Sciences, Faculty of Science The Chinese University of Hong Kong Hong Kong Hong Kong; ^2^ Department of Cell Biology and Neuroscience Rutgers University Piscataway New Jersey USA; ^3^ State Key Laboratory of Agrobiotechnology The Chinese University of Hong Kong Hong Kong Hong Kong; ^4^ Centre for Cell and Developmental Biology The Chinese University of Hong Kong Hong Kong Hong Kong; ^5^ Gerald Choa Neuroscience Centre The Chinese University of Hong Kong Hong Kong Hong Kong

**Keywords:** 1‐carbon metabolism, cell cycle re‐entry, chronic alcohol use, DNA damage response, metabolic reprogramming, neuronal senescence

## Abstract

Chronic binge‐like drinking is a risk factor for age‐related dementia, however, the lasting and irreversible effect of alcohol on the brain remains elusive. Transcriptomic changes in brain cortices revealed pro‐ageing hallmarks upon chronic ethanol exposure and these changes predominantly occur in neurons. The changes are attributed to a prioritized ethyl alcohol oxidation in these cells via the NADPH‐dependent cytochrome pathway. This hijacks the folate metabolism of the 1‐carbon network which supports the pathway choice of DNA repair via the non‐cell cycle‐dependent mismatch repair networks. The lost‐in‐function of such results in the de‐inactivation of the less preferred cell cycle‐dependent homologous recombination (HR) repair, forcing these post‐mitotic cells to re‐engage in a cell cycle‐like process. However, mature neurons are post‐mitotic. Therefore, instead of successfully completing a full round of cell cycle which is necessary for the completion of HR‐mediated repair; these cells are arrested at checkpoints. The resulting persistence of repair intermediates induces and promotes the nuclear accumulation of p21 and cyclin B—a trigger for permanent cell cycle exits and irreversible senescence response. Supplementation of bioactive 5‐methyl tetrahydrofolate simultaneously at times with ethyl alcohol exposure supports the fidelity of the 1‐carbon network and hence the activity of the mismatch repair. This prevents aberrant and irreversible cell cycle re‐entry and senescence events of neurons. Together, our findings offer a direct connection between binge‐drinking behaviour and its irreversible impact on the brain, which makes it a potential risk factor for dementia.

## Graphical abstract

Metabolism of ethyl alcohol in neurons via the NADPH‐dependent CYP2E1 axis contributes to heightened risks of dementia in chronic binge‐like drinking. The findings bridged how the consequential cellular metabolic rewiring cross‐talks to the choice of DNA repair and illustrated how unfavourable deployment of a cell cycle‐based DNA repair network aberrantly leads to neuronal senescence. A metabolic‐correction strategy targeting related metabolic changes offers new perspectives in therapeutic design.

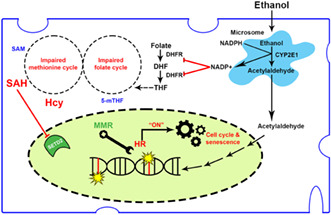

## 1 INTRODUCTION

Over the last few decades, human life expectancy has tremendously increased, resulting in new healthcare challenges and emerging needs to understand the physiological changes behind ageing. Overall, age is the greatest risk factor for dementia (Guerreiro & Bras, 2015), and it is suggested that 40% of the cases worldwide could be prevented by modifying lifestyle risk factors that accelerate body physiological ageing (Livingston et al., 2020). According to the recent recommendations from the Lancet Commission on Dementia Prevention, Intervention and Care, excessive alcohol consumption is now a recognized risk factor for dementia (Livingston et al., 2020). A 10‐year longitudinal study reveals that the absolute alcohol quantity consumed is positively associated with accelerated cognitive and memory decline in midlife even when drinking starts in adolescence (Sabia et al., 2014). Whilst these epidemiological observations are clear, the molecular mechanism of how ethyl alcohol leads to irreversible and persistent changes in brain cells that last even after alcohol abstinence remains poorly understood.

In this study, we report that chronic binge‐like drinking results in global brain transcriptome changes, activating a network of pathways associated with DNA damage‐driven senescence. Further analyses indicate that cortical neurons suffer greatly from the metabolic reprogramming effect induced by the prioritized ethyl alcohol metabolism, which the latter cross‐talks with the choice of DNA repair and cell cycle machinery. The incomplete repair of lesions in these cells via a decision towards a cell cycle‐dependent repair pathway triggers a permanent cell cycle arrest response and cellular senescence. Such changes are unlikely reversible, contributing to lasting impairments in both cognitive and memory functions.

## 2 RESULTS

### 2.1 Chronic binge‐like drinking is associated with lasting cognitive and memory impairments

**FIGURE 1:**
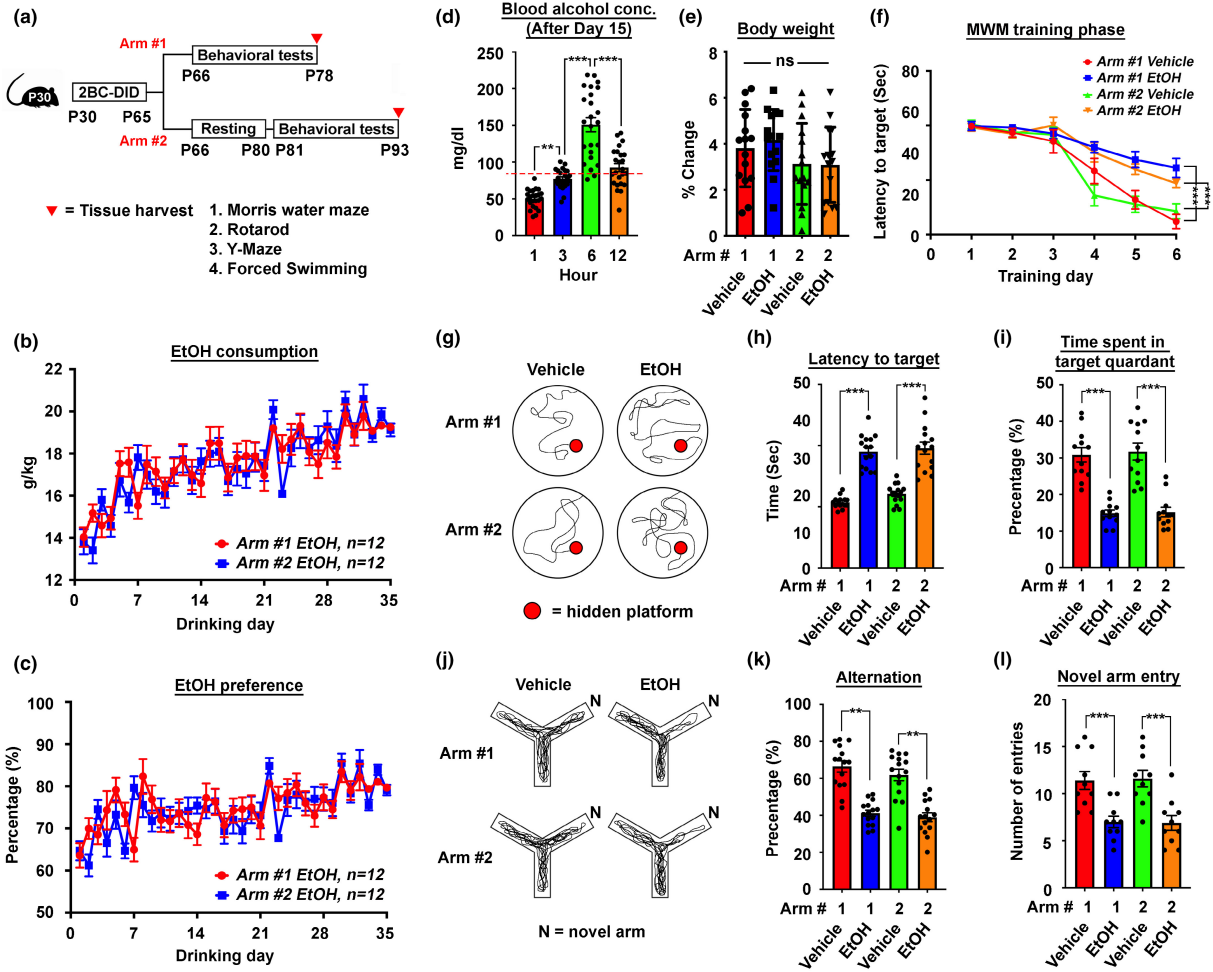
Mouse 2BC‐DID paradigm revealed lasting cognitive and memory impairments. (a) The schematic diagram illustrates the timeline of the 2BC‐DID treatment paradigm with P30 *C57BL/6* mice. Arms #1 and #2 represent two treatment arms with behavioural tests being immediately carried out right after the entire drinking program or being delated after a 14‐day resting period. (b) Ethanol consumption trends and (c) preference of mice in Arms #1 and 2 along the entire 2BC‐DID treatment paradigm (*N* = 12). (d) Blood alcohol concentration of all alcohol‐administering mice in both Arms #1 and #2 at different time points after the initiation of drinking at day 15 (*N* = 24, **p < 0.001, ***p < 0.0001, one‐way ANOVA). (e) Percentage body weight changes in mice from all arms and all treatments measured on the day after Day 35 of the drinking paradigm relative to the day before drinking treatment started (*N* = 12, ns = non‐significant, one‐way ANOVA). (f) The latency to target plot during the training phase (Day 1 to Day 6) in the Morris water maze (MWM) test (*N* = 12, ***p < 0.0001, two‐way ANOVA). (g) Representative swimming patterns of mice in different treatment arms during the MWM probe trial performed on Day 7. (h) The latency to target and (i) the time spent in target quadrant plots of the probe trial in the MWM test (*N* = 12, ***p < 0.0001, one‐way ANOVA). (j) Representative walking patterns of mice in different treatment arms in a Y‐maze paradigm. (k) The percentage of alternation and (l) the number of novel time entry was recorded and calculated (*N* = 12, **p < 0.001, ***P < 0.0001, one‐way ANOVA). Values represent the mean ± SEM.

To interrogate if the observations from the human populational studies can be mimicked by a well‐controlled laboratory model, two‐bottle choice (2BC)‐drinking in the dark (DID) paradigm that examines the binge‐drinking effect was adopted (Sprow & Thiele, 2012; Thiele & Navarro, 2014). Briefly, adolescent P30 mice were given intermittent access to 20% alcohol (v/v) every night for 35 days (5 weeks). Their behavioural changes were either immediately compared with the controls (Figure 1a, Arm #1); or evaluated after being abstinent from drinking for an additional 14 days to investigate if any behavioural changes immediately observed would last (Figure 1a, Arm #2). Alcohol consumption in both cohorts gradually increased with the number of drinking days engaged, with a mean consumption ranging around 13.04–14.03 g/kg during the first day and escalating to 17.56–17.62 g/kg on the last day of the treatment paradigm (Figure 1b). For alcohol preference, the record ranged from 61.21% to 63.59% on day one and escalated to around 74.62%–75.16% on the last day (Figure 1c). On average, the mean blood alcohol concentration (BACs) of these animals reached 77.25 ± 2.681 mg/dl at 3 h into the dark cycle when the drinking started and heightened to 150.9 ± 9.577 mg/dl at 6 h (Figure 1d), confirming that mice under this settings consumed ethyl alcohol in quantities over the threshold for defining binge‐level drinking in humans (i.e., ≥80 mg/dl) (Becker, 2013).

Whilst the changes in body weight before and after the entire 35 days of alcohol administration were not significant amongst all the tested animals (Figure 1e), alcohol drinking did introduce a lasting impact on both cognitive and memory functions, the major higher‐ordered brain functions affected in dementia. Spatial learning was assessed as the latency to target and reference memory was determined by the preference for the platform area when the platform was absent in a Morris Water Maze paradigm. During the 6‐day long training phase, whilst all mice eventually became more familiar with the spatial cues and relative positions of the hidden platform, such improvements were less obvious in those that had undergone the alcohol drinking (Figure 1f). During the probe trial (Day 7), similar results were found in the time latency to the target (Figure 1g,h), as the time spent in the target quadrant became significantly less than that of the water‐drinking controls (Figure 1i). Notably, these trends were also evident in the abstinence arm (i.e., Arm #2), hinting that the impairment was somehow lasting. Apart from this, short‐term memory function was also assessed by the Y‐maze experiments. In the standard spontaneous alternation paradigm, alcohol‐administered mice revealed higher tendencies in re‐entering a recently visited arm (Figure 1k), indicating a less intact working memory and prefrontal cortical functions. In the novel arm entry paradigm, alcohol‐drinking mice again revealed poorer performances (Figure 1l), which is again evident in animals of the abstinence arm, indicating a lasting impairment in short‐term memory as well. Other brain functions, such as depressive‐like behaviour revealed by the mobility time in the forced swimming test (Figure S1a), as well as the motor coordination functions evaluated by the rotarod test (Figure S1b), both domains revealed no obvious differences amongst all the groups, suggested that the lasting impairment on cognitive and memory functions were unique.

### 2.2 Accumulation of senescent‐like neurons characterized by cell cycle and DNA damage response markers upon binge‐like drinking

**FIGURE 2:**
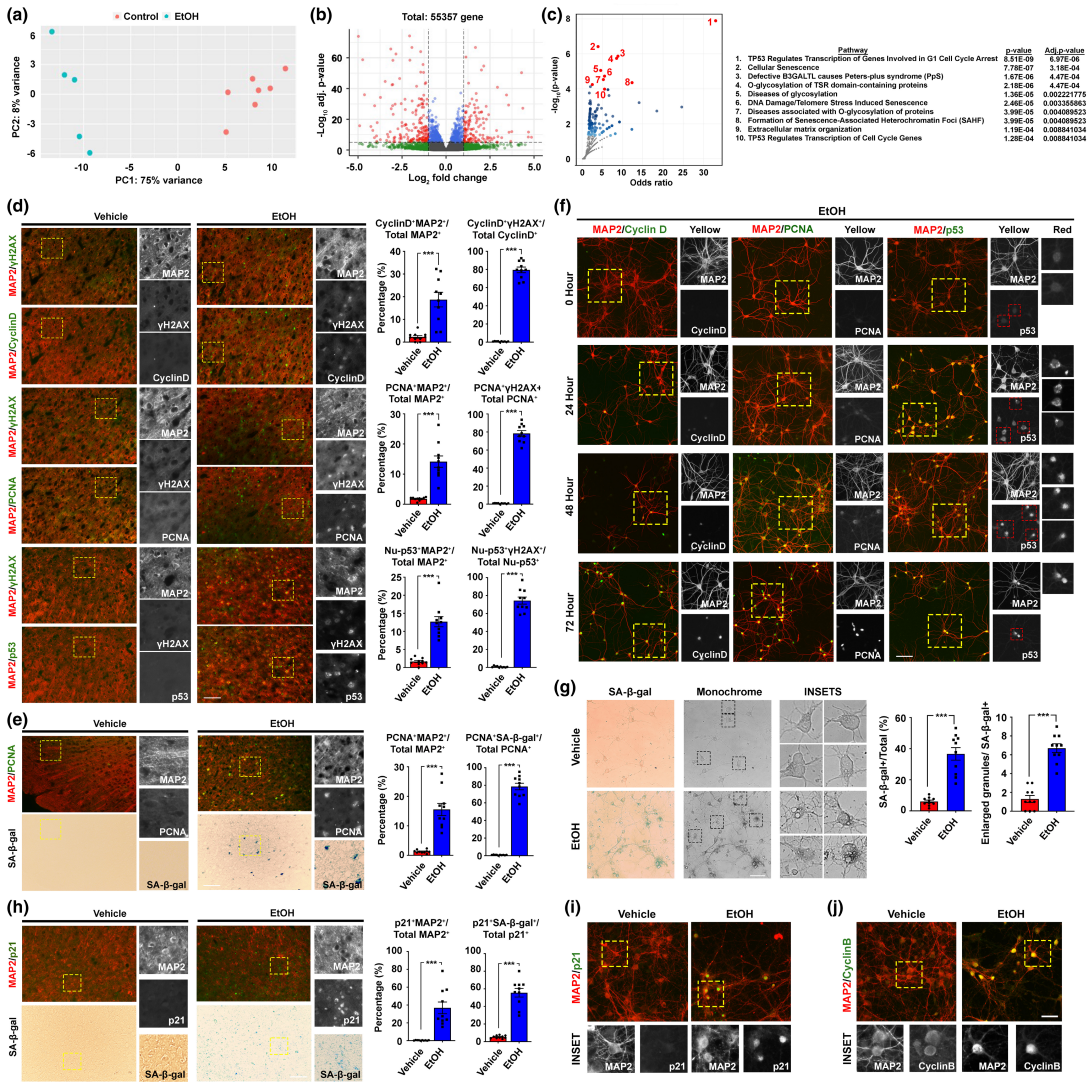
Mouse 2BC‐DID paradigm resulted in a transcriptomic profile supporting neuronal senescence. (a) Principal component plot (PCA) indicated that samples from the control and ethanol‐administered group of Arm #2 were distinctly clustered (*N* = 5–7). (b) The volcano plot indicated that 1643 transcripts were significantly different between the two groups (adjusted p‐value <0.05; Log_2_(fold change) > |0.5|). (c) With the Reactome pathway database, significantly upregulated genes were clustered, and the volcano plot indicated the odds ratio and significance of the enriched pathways. The top 10 pathways were listed. (d) Representative immunofluorescent staining images of the prefrontal cortex regions of brain samples harvested and the corresponding quantifications (*N* = 10, ***p < 0.0001, two‐tailed unpaired *t*‐test, scale bar: 200 μm). (e) Representative SA‐β‐gal and immunofluorescent staining images indicating the relationship between cellular senescence and neuronal cell cycle re‐entry in the prefrontal cortex regions (*N* = 10, ***p < 0.0001, two‐tailed unpaired *t*‐test, scale bar: 200 μm). (f) Representative immunofluorescent staining images of primary neurons subjected to ethanol for different time courses in the CIE treatment paradigm (*N* = 20, scale bar: 100 μm). (g) Representative images of SA‐β‐gal signals in primary neuronal culture subjected to CIE paradigm for 72 h (*N* = 10, ***p < 0.0001, two‐tailed unpaired *t*‐test, scale bar: 100 μm). (h) Representative SA‐β‐gal and immunofluorescent staining images indicating the relationship between SA‐β‐gal signals and other senescence markers (i.e., p21) in the prefrontal cortex region (*N* = 10, ***p < 0.0001, two‐tailed unpaired *t*‐test, scale bar: 200 μm). (i and j) Representative immunofluorescent staining images of primary neurons subjected to exposure to alcohol for 72 h in the CIE treatment paradigm for senescence markers, including nuclear p21 and cyclin B signals (*N* = 20, scale bar: 100 μm). Values represent the mean ± SEM.

To understand the molecular landscape underlies such irreversible behavioural changes, bulk RNA sequencing of mouse cortex tissues harvested Arm #2 animals was performed. With samples of the alcohol‐ and water‐administered groups found distinct from one another (Figure 2a), a total of significantly 823 up‐ and 818 down‐regulated transcripts with |log_2_‐fold change| > 0.5 were identified (Figure 2b, Figure S2a, Table S1). Amongst the significantly downregulated genes, many of them were clustered to the G‐protein‐coupled receptor (GPCR) ligand binding and signalling pathways (Figure S2b,c, Table S2), suggesting a compromised action potential and synaptic communication network in these brains (Gerber et al., 2016). Unexpectedly, upregulated genes indicated activation of a network of pathways associated with DNA damage‐related senescence, p53‐related cell cycle activation and arrest responses (Figure 2c, Table S2). By further immunohistochemistry analyses with antibodies against p53, cyclin D (G1‐phase marker), PCNA (S‐phase marker), cleaved caspase‐3 (CC3, apoptotic marker) and γH2AX (DNA double‐stranded break marker), as well as histology staining of classic senescence‐associated β‐galactosidase (SA‐β‐gal), these confirmed that the changes detected at the transcriptome level were primarily enriched in non‐apoptotic neurons located at the motor‐ and somatosensory‐cortex regions of the prefrontal cortex (Figure 2d,e, Figure S2e,f). Similarly, in DIV10‐12 mouse primary cortical neurons, chronic intermittent ethanol (CIE) treatment involving daily refreshment with 20 mM ethanol (Guo et al., 2012)—a dose slightly above the NIAAA binge‐drinking definition (i.e., Blood EtOH concentration of 17.4 mM) (Chang et al., 2018)—in every 24 h to mimic a stable chronic drinking pattern was performed (Figure S2d). From there, progressive inductions in signal intensities and the proportions of neurons being double positive for MAP2 (neuronal marker) and various cell cycle markers were found (Figure 2f, Figure S3). As early as 24 h of CIE treatment, nuclear signals of cyclin D and cytoplasmic signals of p53 started to emerge, suggesting these neurons were re‐engaging in the G1 phase of the cell cycle. As treatment time prolonged, not only more neurons became positive for these signals, nuclear signals of PCNA and p53 also started to emerge (Figure 2f), hinting some neurons are re‐engaging to this S‐phase, which was supported by the Fucci‐green reporter assay (Figure S2h). As treatment time progressed further, many of these cells started exhibiting classic features of cellular senescence, including lipofuscin aggregates (Figure S2i), SA‐β‐gal signals and enlarged intracellular granules (Figure 2g). Furthermore, molecular markers of senescence, such as nuclear signals of p53 target p21^WAF1/CIP1^ (p21) (Figure 2h,i, Figure S2j,k) (Jurk et al., 2012) and cyclin B (Figure 2j, Figure S2l) (Charrier‐Savournin et al., 2004) were observed. With p21, further analyses revealed that these cells were mainly enriched in the upper cortical layer (i.e., Layer II‐IV) (Figure S4a) and with robust phospho‐Tau signals (Figure S4b)—a newly defined neuronal senescent marker (Dehkordi et al., 2021).

**FIGURE 3:**
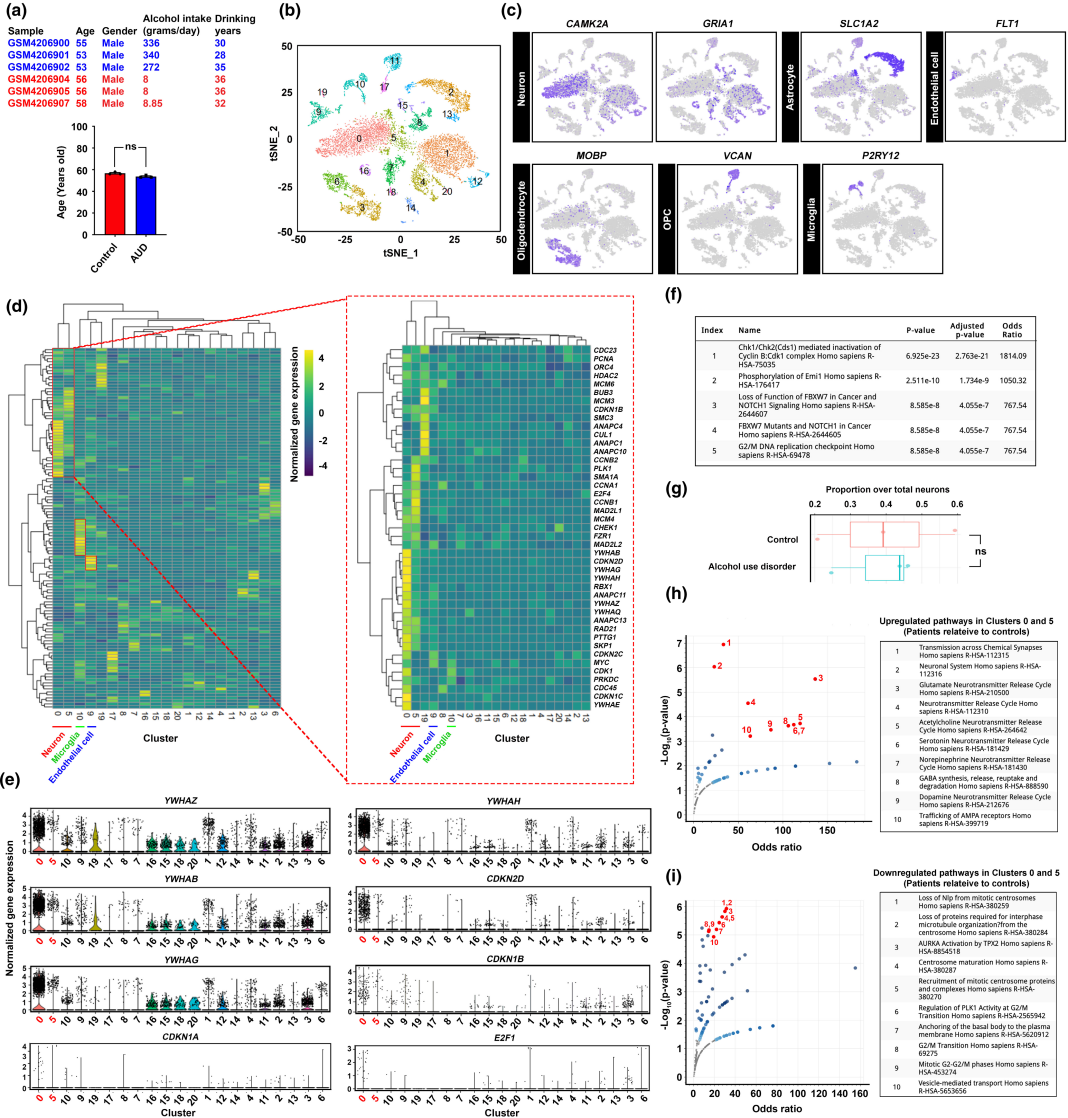
Senescent neurons in the post‐mortem brain prefrontal cortex harvested from individuals with alcohol use disorders revealed unique transcriptomic features. (a) Single‐nuclei transcriptome profiling of an existing public dataset (GSE141552) was performed. Age‐ samples (i.e., control group = 56.67 ± 0.6667; AUD = 53.67 ± 0.6667) and sex‐matched samples were selected and analysed. (b) T‐distributed stochastic neighbour embedding (t‐SNE) plot of all nuclei extracted from the dataset, which was then segregated and coloured as 20 distinct clusters of brain cells, based on their transcriptome features. (c) Based on the known markers of the major cell types, clusters of neurons, astrocytes, endothelial cells, oligodendrocytes, oligodendrocyte progenitor cells (OPCs) and microglia were identified. (d) Targeted analyses of cell cycle‐associated genes obtained from the KEGG database (hsa04110) were performed. Heightened expression of cell cycle‐associated genes was observed in clusters 0 (neuron), 5 (neuron), 10 (microglia) and 10 (endothelial cells). (e) Violin plots indicate the expression levels of a number of cell cycle‐related genes that are mostly enriched in clusters 0 and 5. (f) Pathways of 42 cell cycle‐related genes enriched in clusters 0 and 5 relative to the rest of other neurons were clustered based on the Reactome database. The top 5 enriched pathways were listed. (g) The proportion of clusters 0 and 5 neurons over total neurons in control and AUD samples (*N* = 3, ns = non‐significant, two‐tailed unpaired *t*‐test). (h and i) Comparison of neurons in clusters 0 and 5 between AUD and control patient samples. With the Reactome pathway database, significantly (h) upregulated and (i) downregulated genes were clustered. The volcano plots indicated the odds ratio and significance of the enriched pathways. The top 10 pathways were listed.

To better compare the effect of alcohol intake per day on the brain transcriptome changes in humans, age‐matched samples from a publicly available single nucleus transcriptome dataset were selected for analysis, which contains prefrontal cortex samples isolated from 3 patients (AUD) and 3 control individuals (Figure 3a, one sample was excluded due to the much older age of the patient for >10 years) (Brenner et al., 2020). Unsupervised clustering analysis yielded 20 clusters of cells (Figure 3b). From there, clusters of the six major cell types of the brain were identified based on their specific gene expression markers (Figure 3c, Figure S4c). To investigate their cell cycle status, targeted analyses of cell cycle‐associated genes obtained from the KEGG database (hsa04110) were performed. With a limited number of excitatory neurons of other cortical layers available, Layer IV cells in Clusters 0 and 5 were ones revealed (Figure S4a, Table S3) heightened expression of various cell cycle‐associated genes, with their number even higher than that of the proliferating microglia (Cluster 10) and endothelial cells (Cluster 9) (Figure 3d, red boxes). Notably, several 14–3‐3 family genes (i.e., *YWHAZ, YWHAQ, YWHAG, YWHAH, YWHAE and YWHAB*) which ensure the mitotic (M) phase is not prematurely activated were uniquely enriched in these neuronal clusters (Brunet et al., 2002). Similarly, the expression of multiple cyclin‐dependent kinase inhibitors (i.e., *CDKN2D*, *CDKN2C* and *CDKN1B*) which mediate the initiation of cell cycle checkpoints were also found (Figure 3e). Further investigation of these clusters 0 and 5 neurons revealed that many were directly involved in a network of pathways initiating the G2/M checkpoint, including the “checkpoint kinase‐1/‐2 (Chk1/Chk2)‐mediated inactivation of Cyclin B:Cdk1 complex” to prevent M‐phase onset; “phosphorylation of early mitotic inhibitor (Emi1)” that facilitates the inactivation of G2/M‐promoting APC/C; and “G2/M DNA replication checkpoint” signalling (Figure 3f). The relative abundance of neurons in Clusters 0 and 5 trended higher in the patient group over the control, despite the number was not statistically significant probably due to the limited sample sizes (Figure 3f). Qualitatively, 80 differentially expressed genes were identified in these cells between the unaffected and affected groups (Table S4). In the affected group, genes upregulated may promote hyperexcitability due to their involvement in multiple neurotransmitter releases pathways (Figure 3h). On the contrary, genes downregulated were one involved in G2/M phase transition and the mitotic centrosome maturation, which could prevent mitotic phase entry but support a robust G2/M cell cycle arrest status amongst these cells (Figure 3i).

### 2.3 Ethanol metabolism hijacks cellular NADPH/NADP ^+^ balance critical for sustaining the 1‐carbon metabolic network

**FIGURE 4:**
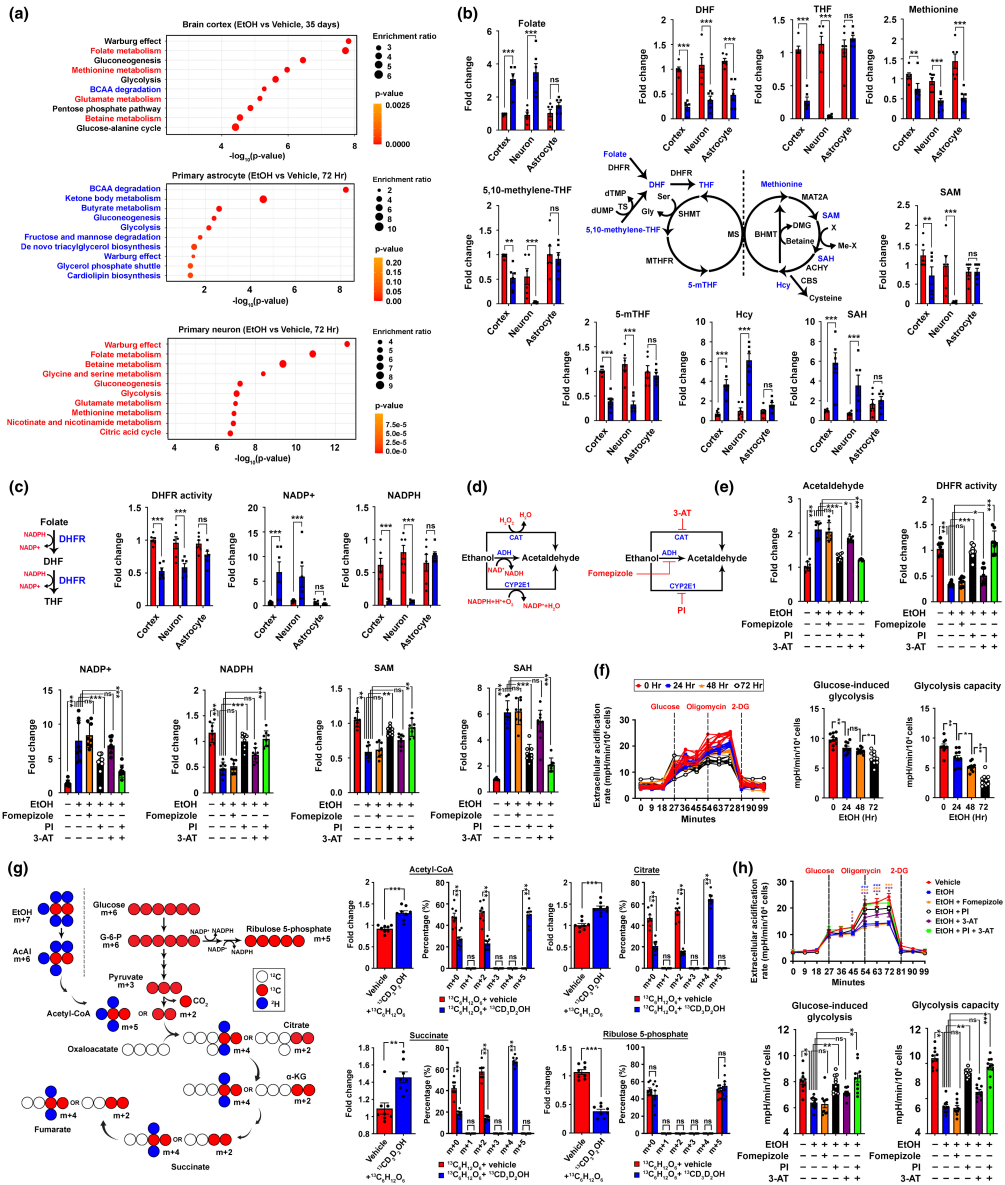
CYP2E1‐dependent ethanol metabolism hijacks the NADPH/NADP+ homeostasis and global metabolic landscape in neurons. (a) Global metabolic profiling of freshly harvested mouse brain cortex tissues right after completing the entire 2BC‐DID treatment paradigm (Arm #1), so as primary astrocytes and neurons after being subjected to the CIE treatment for 72 h was performed. Differentially changed metabolites were clustered and analysed by the metabolite set enrichment analysis (MESA). Pathways were ranked according to the significance values (*N* = 6). (b) Schematic diagram of the folate‐methionine cycle, detailed changes in key metabolites involved were shown (*N* = 6, **p < 0.001, ***p < 0.0001, two‐tailed unpaired *t*‐test within specific type of samples). (c) Schematic diagram showing the reactions involved in converting folate to DHF and then THF by the common DHFR enzyme. Quantifications of DHFR activities, NADP+ and NADPH levels were performed (*N* = 6, ***p < 0.0001, ns = non‐significant, two‐tailed unpaired *t*‐test). (d) Schematic diagram showing the possible reactions in converting ethanol to acetaldehyde, and the specific inhibitors against these reactions (PI: Phenethyl isothiocyanate; 3‐AT: 3‐amino‐1,2,4‐triazole). (e) Changes in DHFR activities so as the levels of acetaldehyde, NADP+, NADPH, SAM and SAH were analysed in primary neurons upon CIE and various drug treatments for 72 h (50 μM fomepizole, 10 μM PI and 20 μM 3‐AT) as indicated (*N* = 8, ***p < 0.0001, **p < 0.001, *p < 0.01, ns = non‐significant, one‐way ANOVA). (f) Glycolytic functions of primary neurons subjected to the CIE paradigm for various time course was evaluated. Glucose‐induced and maximal glycolytic capacities were calculated (*N* = 10, **p < 0.001, *p < 0.01, ns = non‐significant, one‐way ANOVA). (g) Schematic presentation of the oxidative reactions of glucose versus ethanol flux into the central carbon metabolic network. Mass isotopologue analysis of acetyl‐CoA, citrate, succinate and ribulose‐5‐phosphate in primary neurons exposed to glucose‐^13^C_6_ isotope alone or simultaneously with ethanol‐^13^C_2_,1,1,2,2,2‐d_5_ for 2 h (*N* = 8, ***p < 0.0001, **p < 0.001, ns = non‐significant, two‐tailed unpaired *t*‐test). (h) In primary neurons subjected to the CIE paradigm co‐treated with various small molecules for 72 h (50 μM fomepizole, 10 μM PI and 20 μM 3‐AT) as labelled, glycolytic functions against time, glycose‐induced and maximal glycolytic capacities were evaluated (*N* = 10, ***p < 0.0001, **p < 0.001, *p < 0.01, ns = non‐significant, one‐way ANOVA). Values represent the mean ± SEM.

To understand how alcohol administration could lead to the above changes in neurons, we investigated the immediate reactions that happen to ethyl alcohol (i.e., ethanol) once it reaches the cells. Ethanol is a two‐carbon‐containing amphiphilic compound, allowing it to pass through the cell membrane lipid bilayer with little restrictions (Kumari et al., 2018) and be subsequently metabolized. Like other macronutrients, ethanol may serve as a source of metabolic carbon; but unlike others, it is non‐storable in mammalian cells (Schutz, 2000). By global metabolite profiling of brain cortex tissues, the alcohol‐administered group revealed substantially different metabolite landscapes (Table S5). Changes were found enriched to the 1‐carbon network (i.e., folate, methionine and betaine metabolism) and second to the glucose‐related metabolism (i.e., Warburg effect, gluconeogenesis, glycolysis, pentose phosphate pathway and glucose‐alanine cycle) (Figure 4a). Similar analyses were also performed with metabolites extracted from primary neurons and astrocytes—the major metabolically active cells—after the CIE paradigm for 72 h (Table S5). Intriguingly, whilst changes related to glucose‐centric metabolic pathways were found in both cell types, those involved in the 1‐carbon network were uniquely enriched to neurons (Figure 4a,b) and were not caused by altered expression of associated enzymes (Figure S5). The 1‐carbon metabolism metabolites commonly upregulated in neurons and brain tissues included folate, s‐adenosylhomocysteine (SAH) and homocysteine (Hcy); whereas other metabolites in the same network were consistently diminished as well (Figure 4b). At the open‐ended point of the folate cycle, folate was aberrantly accumulated whilst its immediate downstream dihydrofolate (DHF) and 5‐methyl‐tetrahydrofolate (5‐mTHF) were however reduced (Figure 4b). Further investigations indicated that such changes were likely associated with reduced activities of the NADPH‐dependent dihydrofolate reductase (DHFR) enzyme, caused by the diminished availability of its coenzyme substrate NADPH (Figure 4c).

The homeostasis of NADPH is determined by its net rates of production and consumption. One major consumption route activated under this scenario is potentially the ethyl alcohol metabolism, which can be conducted by 3 different enzymes: alcohol dehydrogenase (ADH), catalase (CAT) and the NADPH‐dependent CYP2E1 (P4502E1). To understand how neurons metabolize ethanol, pharmacological inhibitors against these enzymes were co‐treated during the CIE paradigm (Figure 4d). Changes in levels of acetaldehyde—the immediate downstream product of ethanol oxidation, so as the DHFR activity were the most dramatic when P4502E1 was pharmacologically antagonized (Figure 4e). Similar reversal effects were found in NADP+, NADPH, SAM and SAH levels as well, confirming that the altered levels of these metabolites in neurons were downstream to ethanol oxidation via the CYP2E1 axis. Whilst such CYP2E1 antagonization could directly halt the aberrant consumption of NADPH, it is also plausible that NADPH level restoration was also in part contributed by its massive production from the glucose‐driven pentose phosphate pathway (PPP) (Chen et al., 2019). Utilizing the neuronal CIE model, glycolytic rate was gradually reduced upon chronic ethanol exposure (Figure 4f), matching the findings in the global metabolomic analysis (Figure 4a). Further, stable isotope tracing was performed to trace down the immediate fates and competition between ethanol‐^13^C_2_,1,1,2,2,2‐d_5_ and glucose‐^13^C_6_ isotopes in neurons (Figure 4g). It was expected that when ethanol metabolism dominated, this would preferentially yield the (m + 5) acetyl‐CoA and (m + 4) forms of tricarboxylic acid (TCA) cycle intermediates (i.e., citrate and succinate). In contrast, if glucose metabolism dominates, this would give rise to (m + 2) forms of all these TCA cycle metabolites instead (Figure 4g). Upon 2 h of exposure, isotopologue profiling revealed that when both heavy isotopes were simultaneously added, (m + 5) acetyl‐CoA and (m + 4) TCA metabolites were instantly found, indicating ethanol metabolism acutely dominated over glycolysis (Figure 4g). Along the same vein, levels of (m + 5) ribulose 5‐phosphate—a glucose‐derived metabolite in the PPP—were diminished (Figure 4g). However, when CYP2E1 was antagonized, glucose metabolic activities in these cells were preserved (Figure 4h). These together indicated that the dominance of ethanol metabolism via the CYP2E1 axis is the major cause of neuronal NADPH 1‐carbon metabolic dyshomeostasis.

### 2.4 Altered pathway choice of DNA cross‐link repair under a disrupted 1‐carbon network leads to persistent repair intermediates and senescence responses

**FIGURE 5:**
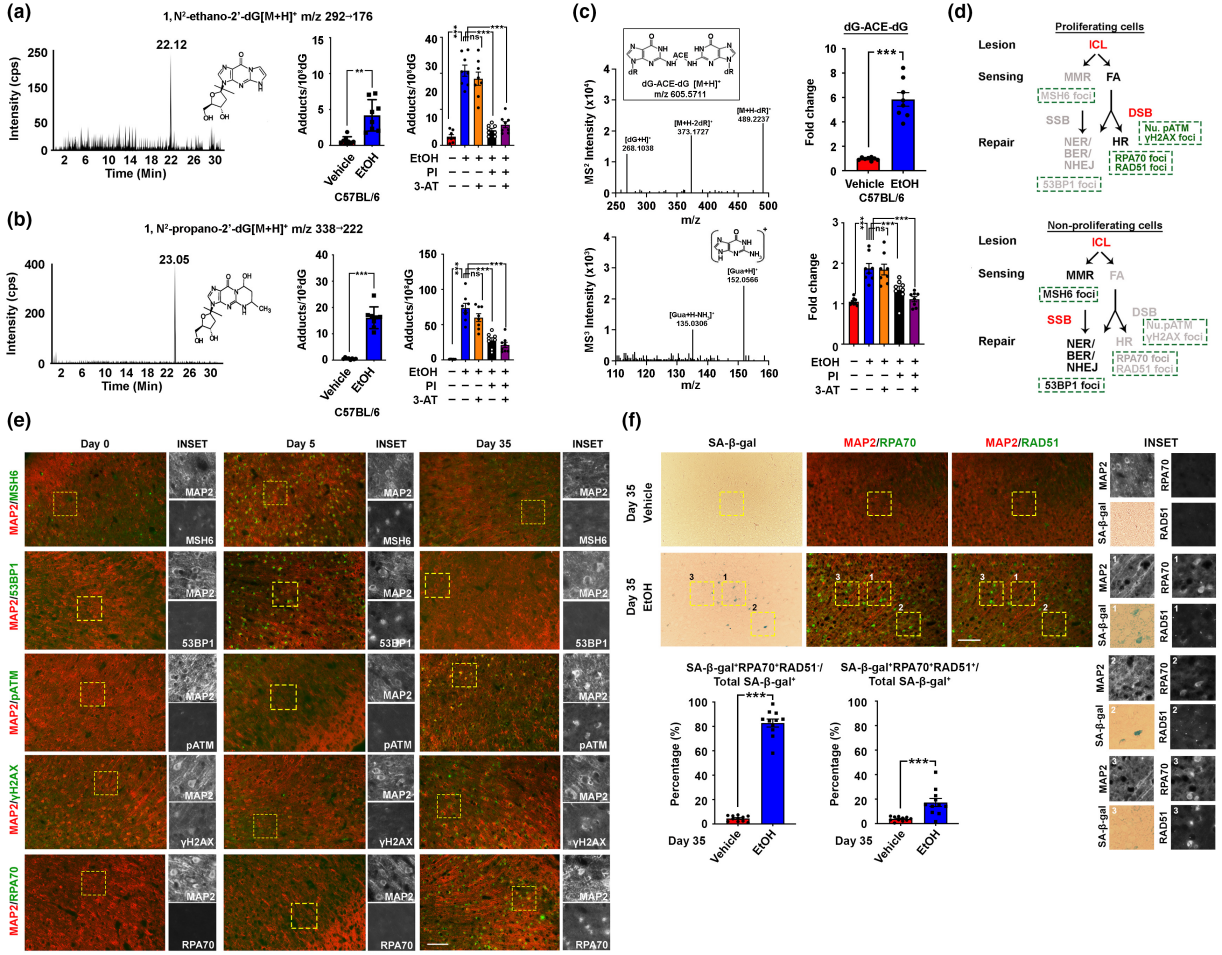
Prolonged ethanol exposure results in DNA‐repair infidelity in cortical neurons. (a) Quantifications of 1, N^2^‐ethanol‐2′‐dG and (b) 1, N^2^‐propano‐2′‐dG adducts in brain cortical tissues (*N* = 8, ***p < 0.0001, two‐tailed unpaired *t*‐test) and primary cortical neuron subjected to the CIE and drug co‐treatment for 72 h (10 μM PI and 20 μM 3‐AT) (*N* = 8, ***p < 0.0001, **p < 0.001, *p < 0.01, one‐way ANOVA). (c) Product ion spectra of acetaldehyde‐induced DNA crosslinks measured as dinucleosides. Quantification of their relative abundances in brain cortical tissues (*N* = 8, ***p < 0.0001, two‐tailed unpaired *t*‐test) and primary cortical neuron subjected to the CIE and drug co‐treatment for 72 h (10 μM PI and 20 μM 3‐AT) (*N* = 8, ***p < 0.0001, **p < 0.001, *p < 0.01, one‐way ANOVA). (d) Schematic diagram showing the possible repair pathways for ICL lesions in proliferating and non‐proliferating cells. (e) Representative immunofluorescent staining images of the prefrontal cortex regions of brain samples harvested from mice subjected to different length periods of the 2BC‐DID paradigm (*N* = 10, scale bar: 200 μm). (f) Representative SA‐β‐gal and immunofluorescent staining images indicating the relationship between cellular senescence and the failure of RPA1 (i.e., RPA70) and RAD51 exchange in neurons. Quantifications were shown (*N* = 10, ***p < 0.0001, two‐tailed unpaired *t*‐test, scale bar: 200 μm). Values represent the mean ± SEM.

Alongside with metabolic rewiring effect, another immediate outcome of prioritized ethanol oxidation is the production of acetaldehyde, a potent inducer of DNA adducts (Brooks & Theruvathu, 2005). In brain cortex tissues harvested in Arm #2, adducts such as 1, N^2^‐ethano‐2′‐dG and 1, N^2^‐propano‐2′‐dG were significantly elevated upon ethanol administration (Figure 5a,b, Figure S6a,b). Similar observations were found in primary neurons subjected to the CIE paradigm for 72 h (Figure 5a,b, Figure S6). Amongst the adducts, 1, N^2^‐Propano‐2′‐dG was aberrantly enriched, which is a potent primer for the formation of inter‐strand crosslinks (Figure 5c, Figure S6c–g). In alignment with the observations above, the accumulation of ICLs in neurons was reduced when CYP2E1 was effectively antagonized (Figure 5c, Figure S6c–g).

Previous studies suggested that Fanconi anaemia (FA) is the major crosslink sensing mechanism in proliferative cells (Lopez‐Martinez et al., 2016), as subsequent utilization of the cell cycle‐dependent HR machinery for repair is possible (Michl et al., 2016). In non‐mitotic cells, however, the mismatch repair (MMR) sensing (Kato et al., 2017) followed by the recruitment of cell cycle‐independent excision‐based repair is much preferred (Lai et al., 2016; SenGupta et al., 2013) (Figure 5d). In mice, a short‐term 2BC‐DID for 5 days yielded signals indicating an activated MMR‐mediated repair network (i.e., MSH6, 53BP1) (Figure 5e, Figure S7a). However, as 2BC‐DID prolonged to 35 days, these signals gradually diminished but those indicating an activated FA‐mediated HR axis emerged instead (i.e., pATM, γH2AX, RPA70) (Figure 5e, Figure S7a). Indeed, the timing when cell cycle‐reengaged neurons emerged was closely associated with the activation of the FA‐HR axis (Figure S7b). During HR, resections of the DNA are introduced at the break sites, creating extensive single‐stranded overhangs covered with RPA1 proteins, which shall then be exchanged by the RAD51 protein for completing the repair (Feringa et al., 2018). Upon completing the entire 2BC‐DID, nuclear signals of RPA70—the 70 kDa DNA‐binding subunit of the RPA1 heterotrimeric complex—were abundantly seen (Figure 5e,f), however parallel induction of RAD51 foci was not equally robust, as reflected by the lack of co‐localizing RAD51 signals in most RPA70‐positive neurons (Figure 5f). Notably, these RPA70^+^RAD51^−^ neurons were positive for the SA‐β‐gal whilst those successfully recruited RAD51 (i.e., RPA70^+^RAD51^+^) were not (Figure 5f). This indicated that when stretches of resected DNA covered with RPA1 protein failed to properly engage in the subsequent steps of the HR (i.e., recruitment of RAD51), they were likely remained as persistent repair intermediates, driving permanent cell cycle arrest and cellular senescence.

**FIGURE 6:**
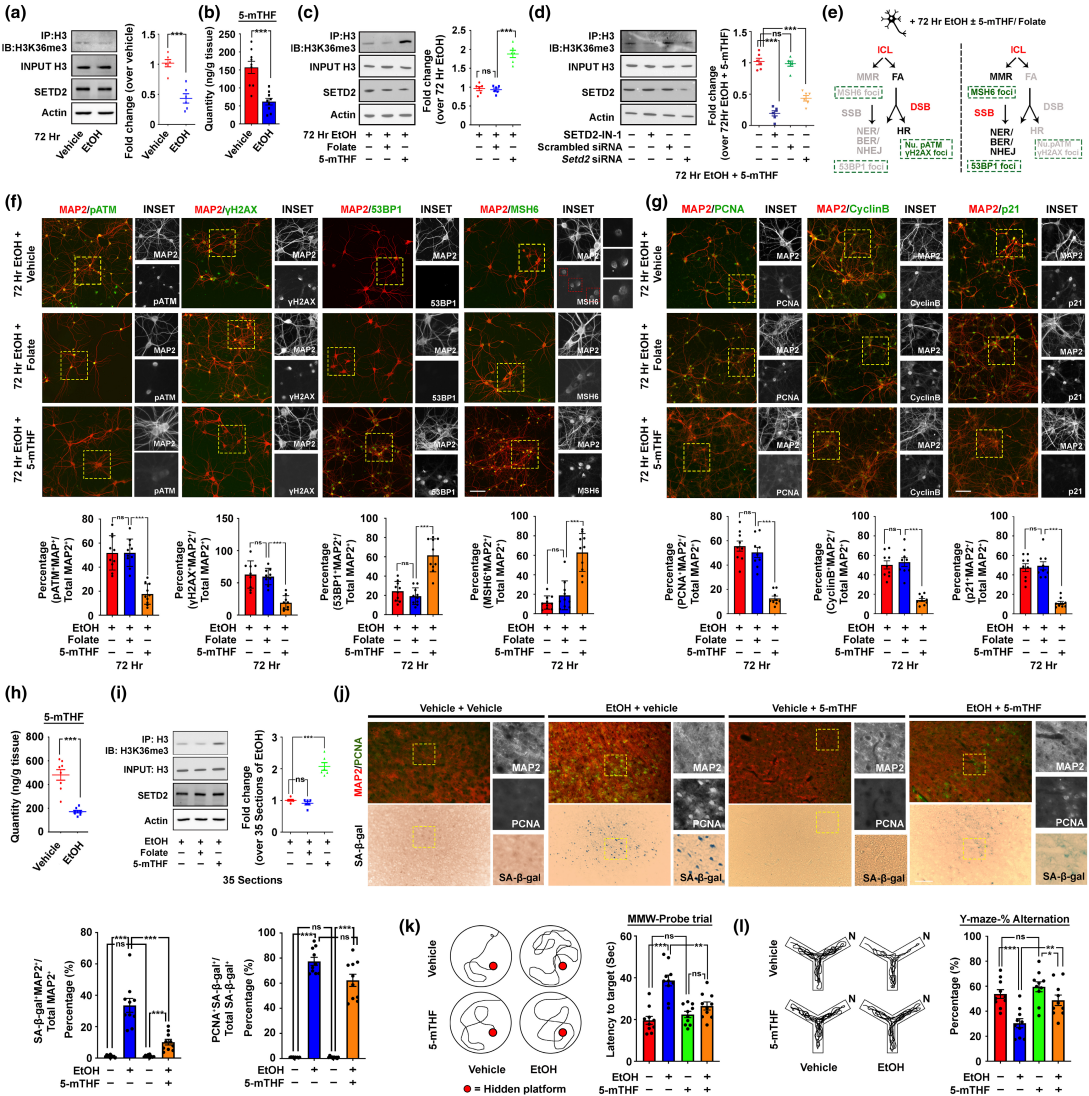
5‐methyl tetrahydrofolate (5‐mTHF) supplementation supports mismatch repair signalling, and prevents neuronal cell senescence and brain function decline induced by chronic ethanol exposure. (a) Representative Western blots show that CIE treatment for 72 h in primary neurons results in reduced activities of SETD2, as reflected by reduced levels of H3K36me3. Quantification of the relative band intensities between H3K36me3 over total histone H3 levels was shown (*N* = 6, ***p < 0.0001, two‐tailed unpaired *t*‐test). (b) Quantification of 5‐mTHF in primary neurons subjected to 72 h of CIE treatment (*N* = 9, ***p < 0.0001, two‐tailed unpaired *t*‐test). (c) Representative Western blots showing reduced activities of SETD2 in primary neurons subjected to CIE treatment for 72 h was prevented by co‐administration of 100 nM 5‐mTHF but not the same quantity of folate. Quantification of the relative band intensities between H3K36me3 over total histone H3 levels was shown (*N* = 6, ***p < 0.0001, ns = non‐significant, one‐way ANOVA). (d) Representative Western blots showing the effect mediated by 100 nM 5‐mTHF on H3K36me3 was dependent on active SETD2 enzyme. Quantification of the relative band intensities between H3K36me3 over total histone H3 levels was shown (*N* = 6, ***p < 0.0001, ns = non‐significant, one‐way ANOVA). (e) Schematic diagram showing the possible repair pathway choices for ICL lesions in neurons subjected to CIE and 100 nM 5‐mTHF supplementation. (f and g) Representative immunofluorescent staining images of markers in (f) different repair ICL repair pathways, (g) cell cycle reengagement and cellular senescence in primary neurons subjected to the exposure of alcohol for 72 h in the CIE treatment paradigm. Quantifications of relative cell populations were shown below (*N* = 10, ***p < 0.0001, ns = non‐significant, one‐way ANOVA, scale bar: 100 μm). (h) Quantification of 5‐mTHF in brain cortex tissues subjected to the entire 2BC‐DID paradigm (*N* = 9, ***p < 0.0001, two‐tailed unpaired *t*‐test). (i) Representative Western blots showing the reduction in activities of SETD2 in the brain cortex subjected to the entire 2BC‐DID was prevented by intra‐nasal co‐administration of 100 ng/day of 5‐mTHF but not the same quantity of folate (*N* = 6, ***p < 0.0001, ns = non‐significant, one‐way ANOVA). (j) Representative SA‐β‐gal and immunofluorescent staining images indicating cellular senescence and neuronal cell cycle re‐entry phenomenon in the prefrontal cortex regions were alleviated upon intra‐nasal co‐administration of 100 ng/day 5‐mTHF (*N* = 10, ***p < 0.0001, own‐way ANOVA, scale bar: 200 μm). (k) Representative swimming patterns of mice revealed that the lasting impact of chronic binge‐like drinking was alleviated upon intra‐nasal co‐administration of 100 ng/day 5‐mTHF. The latency to the target of the probe trial in the MWM test was quantified (*N* = 10, ***p < 0.0001, **p < 0.001, ns = non‐significant, one‐way ANOVA). (l) Representative walking patterns of mice revealed that the lasting impact of chronic binge‐like drinking was alleviated upon intra‐nasal co‐administration of 100 ng/day 5‐mTHF. The percentage of alternation was recorded and calculated (*N* = 10, ***p < 0.0001, **p < 0.001, *p < 0.01, ns = non‐significant, one‐way ANOVA). Values represent the mean ± SEM.

With these findings, we speculated that the ultimate emergence of senescent neurons was likely due to the infidelity of MMR‐mediated repair in the first place caused by a failure in folate metabolism. At the molecular level, this could be related to the dynamic generation of histone H3 K36 trimethylation (H3K36me3) which marks sites needing repair (Huang et al., 2018; Li et al., 2013) by the SET domain containing‐2 (SETD2) enzyme whose activity depends heavily on SAM but inhibited by SAH (S. Yang et al., 2016). As hinted from the metabolomics, chronic ethanol administration introduced an environment unfavourable for SETD2 (Figure 4a,b). In line with this, 72 h of the CIE paradigm resulted in significant reductions in the H3K36me3 level (Figure 6a). This phenomenon was likely due to the failure in converting folate to the bioactive 5‐mTHF (Figure 6b), as an excess 5‐mTHF but not folate supplementation (i.e., 100 nM) along with ethanol exposure was effective in restoring H3K36me3 (Figure 6c). The role of SETD2 was also confirmed with the pharmacological antagonist or specific silencing RNAs (Figure 6d). Further downstream analyses indicated that 5‐mTHF supplementation was able to maintain an activated MMR response, allowing the HR repair remained quiescent (Figure 6e,f). The treatment also diminished the number of neuronal cell cycle (i.e., PCNA) and senescent events (i.e., nuclear cyclin B and p21) (Figure 6g). When extended to in vivo where 5‐mTHF was intranasally supplemented (i.e., 100 ng/day) during the entire 2BC‐DID paradigm (Figure 6h, Figure S2a,b), similar protective effects were found at both the molecular and cell levels (Figure 6h–j, Figure S8c); leading to a less severe lasting functional decline in both the memory (Figure 6k) and cognitive (Figure 6l) domains.

### 2.5 Common *ALDH2* loss‐of‐function mutation exacerbates neuronal senescence, but could be alleviated by a metabolic drug‐nutrient dyad strategy

**FIGURE 7:**
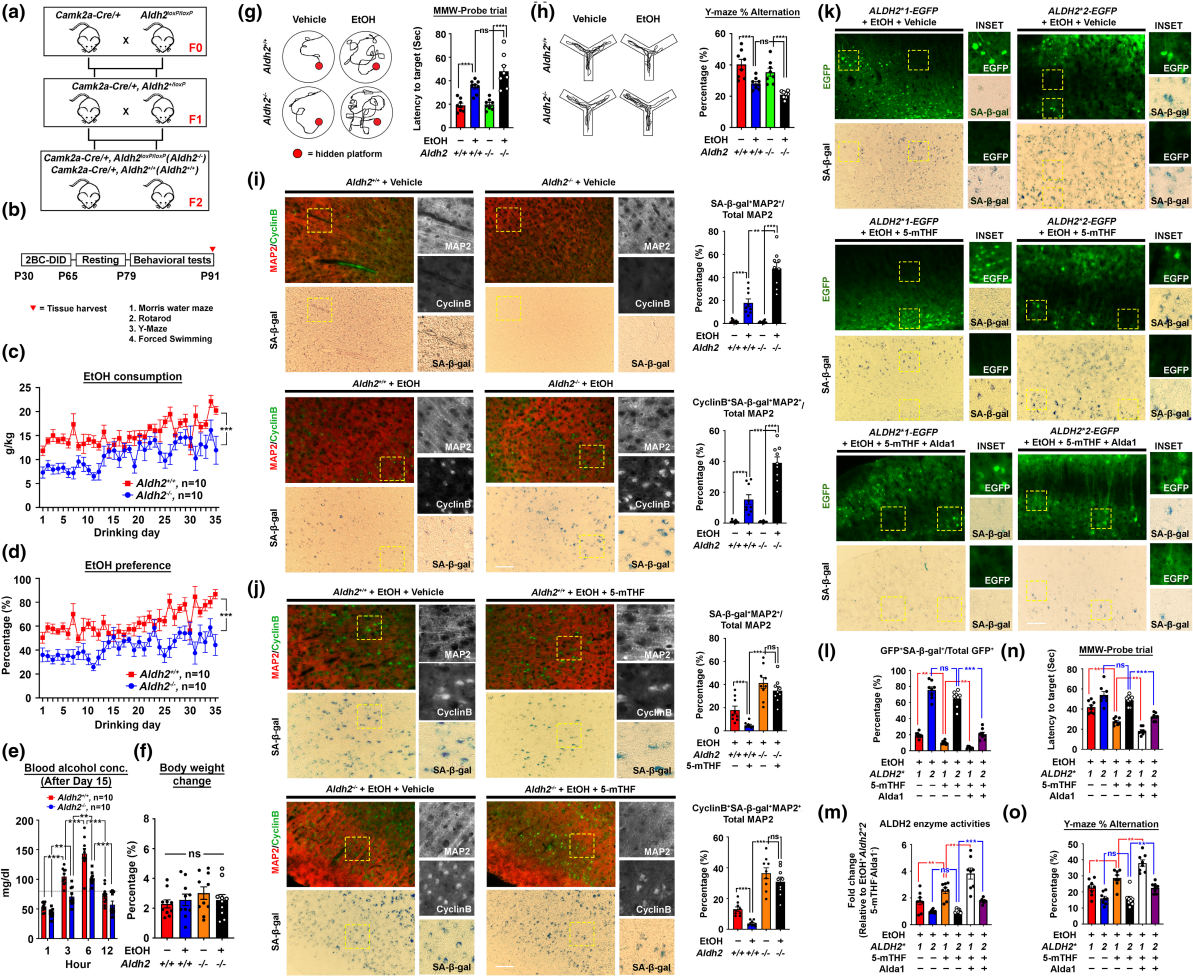
The common ALDH2 loss‐of‐function mutation exacerbates DNA damage‐associated stress and neuronal senescence but was alleviated by a metabolic drug‐nutrient dyad strategy. (a) Schematic diagram showing the mouse breeding scheme. (b) By 3 months of age, mice were subjected to the 2BC‐DID paradigm with or without intranasal drug supplementation, followed by 14 days of alcohol abstained period before undergoing thorough behavioural testing to evaluate the effect of neuronal *Aldh2* knockout at both cell and behaviour levels. (c) Ethanol consumption trends and (d) preference of mice along the entire 2BC‐DID treatment paradigm (N = 10). (e) Blood alcohol concentration of all alcohol‐administering mice at different time points after the initiation of drinking on Day 15 (*N* = 24, **p < 0.001, ***p < 0.0001, one‐way ANOVA). (f) Percentage body weight changes in mice from all arms and all treatments measured on the day after drinking Day 35 relative to the day before the drinking treatment started (*N* = 10, ns = non‐significant, one‐way ANOVA). (g) Representative swimming patterns in the MWM probe trial, and latencies to target were quantified (*N* = 8, ***p < 0.0001, ns = non‐significant, one‐way ANOVA). (h) Representative walking patterns in the Y‐maze paradigm, percentage of alternation was quantified (*N* = 8, ***p < 0.0001, ns = non‐significant, one‐way ANOVA). (i) Representative brain section images illustrating the cyclin B‐ and SA‐β‐gal‐double positive neuronal loads in the prefrontal cortex regions of *Aldh2* ^*+/+*^ *and Aldh2* ^*−/−*^ mice after the entire 2BC‐DID treatment paradigm (*N* = 9, ***p < 0.0001, **p < 0.001, one way ANOVA, scale bar: 200 μm). (j) Representative brain section images illustrating the effect of 5‐mTHF supplementation on the accumulation of cyclin B‐ and SA‐β‐gal‐double positive neurons after the 2BC‐DID treatment paradigm (*N* = 9, ***p < 0.0001, ns = non‐significant, one‐way ANOVA, scale bar: 200 μm). (k–o) By re‐expressing the human ALDH2*1 or ALDH2*2 variant specifically into prefrontal cortex neurons of *Aldh2* ^*−/−*^ mice, supplementation of 100 ng/day 5‐mTHF ±25 μg Alda1 or vehicle in 10 μl total volume along the entire 2BC‐DID treatment paradigm was performed. (k) Representative images and (l) quantification of the proportion of SA‐β‐gal and GFP double‐positive neurons were shown (*N* = 8, ***p < 0.0001, **p < 0.001, ns = non‐significant, one‐way ANOVA, scale bar: 200 μm). (m) the overall brain cortex ALDH2 activities, (n) behavioural performances in MWM and (o) Y‐maze paradigms were analysed (*N* = 8, ***p < 0.0001, **p < 0.001, *p < 0.01, ns = non‐significant, one‐way ANOVA). Values represent the mean ± SEM.

Our data by far demonstrated that the dominance of ethanol oxidation can promote DNA damage‐associated neuronal senescence. The accumulation of these non‐dying but dysfunctional cells is potentially a factor contributing to lasting functional impairments. This suggested that any factors that exacerbate such genomic stress would aggravate the damaging effect. The second reaction of ethanol oxidation involves the conversion of DNA‐damaging acetaldehyde to a non‐genome toxic acetate, catalysed mainly by the mitochondrial aldehyde dehydrogenase (ALDH2) which is mutated in around 8% of the global population as a semi‐dominant, non‐functional variant (ALDH2*2 Glu504Lys) (Hirohashi et al., 2020). In mice, *Aldh2* is predominantly expressed in neurons located at the prefrontal cortex (Figure S9a). Based on that, a conditional knockout mouse model where the *Aldh2* gene in the forebrain neuronal lineage being knockout was generated (Figure 7a, Figure S9b), and subjected to the same 2BC‐DID paradigm as performed to the wildtype (*Aldh2* ^*+/+*^) mice (Figure 7b).

Like the wildtype *Aldh2* ^*+/+*^mice, alcohol consumption and preference increased as drinking days prolonged but such changes were less obvious in the *Aldh2*‐conditioned knockout (*Aldh2* ^*−/−*^) mice (Figure 7c,d). Likely because of that most of that in *Aldh2* ^*+/+*^ animals had their mean blood alcohol concentration reached over the human binge‐level drinking threshold (i.e., ≥80 mg/dl) by 3 h after drinking started; it, however, took around 6 h to achieve so in the *Aldh2* ^*−/−*^ mice (Figure 7e). These suggested that selective loss of *Aldh2* in neurons was somehow protective against the degree of alcoholism in mice, similar to the case found in humans (Edenberg, 2007). Despite so, levels of acetaldehyde accumulated in the prefrontal cortex harvested from the *Aldh2* ^*−/−*^ mice were much higher even with much lower levels of alcohol consumption (Figure S9c); and their acetate content trended in the reverse manner (Figure S9c). On the contrary, brain levels of NADPH, NADP+, 5‐mTHF and DHFR activities altered almost in similar extents in both *Aldh2* genotypes (Figure S9d), implying that the degree of changes in NADPH homeostasis was similar regardless of the ALDH2 status. Whilst the overall differences in body weights at times before and after the entire 2BC‐DID remained insignificant amongst all the testing groups (Figure 7f), behavioural performance related to spatial learning (Figure 7g), working memory and prefrontal cortical functions (Figure 7h) were more deteriorated in the *Aldh2* ^*−/−*^ mice, which was associated with aggravated accumulation of acetaldehyde (Figure S9c), DNA adducts (Figure S9e) and inter‐strand crosslinks (Figure S9f). Suffering from such a heightened degree of DNA damage‐related stress, the *Aldh2* ^*−/−*^ mice revealed higher loads of nuclear cyclin B‐ and SA‐β‐gal‐positive senescent neurons (Figure 7i), explaining the poorer functional performances amongst these mice.

Similar to the 5‐mTHF protective effect observed in the C57BL/6 mice (Figure 6i–l, Figure S8c), daily intranasal supplementation of 100 ng/day 5‐mTHF was able to alleviate loads of SA‐β‐gal^+^ (Figure 7j), nuclear cyclin B^+^ (Figure 7j) and γH2AX^+^ (Figure S9g) senescent neurons in the *Aldh2* ^*+/+*^ mice. However, changes were not as obvious in the *Aldh2* ^*−/−*^ mice (Figure 7j, Figure S9g), except when the wildtype ALDH2*1 but not the functionally defective ALDH2*2 variant was ectopically re‐expressed (Figure 7k, Figure S9h,i). By realizing an existing pharmacological ALDH2 agonist—Alda1—which could activate the enzyme regardless of its variant forms (Perez‐Miller et al., 2010); and our goal of facilitating the normal DNA repair whilst limiting the DNA lesion produced, a modified strategy which combined 100 ng/day 5‐mTHF with 25 μg/day Alda1 was tested. In *Aldh2* ^*−/−*^ mice with ectopic re‐expression of ALDH2*1, the modified drug‐metabolite combined strategy revealed a better efficacy in reducing a load of SA‐β‐gal^+^ senescent neurons (Figure 7k,l). Similarly, in *Aldh2* ^*−/−*^ mice with ectopic re‐expression of ALDH2*2, administration of Alda1 was able to sensitize the effect of 5‐mTHF via the re‐activation of the ALDH2*2 defective variant (Figure 7m), resulting in reduced loads of SA‐β‐gal^+^ senescent neurons (Figure 7k,l). Such changes were extended to better preservation in both cognitive and memory functions amongst these mice (Figure 7n,o).

## 3 DISCUSSION

This work demonstrates how the metabolism of ethyl alcohol in neurons chronically alters their physiological metabolic network and cross‐talks to aspects related to genome integrity, DNA‐damage response and cellular senescence. As a metabolite, ethanol oxidation immediately yields acetaldehyde in neurons, via predominantly the actions of NADPH‐dependent CYP2E1. In these cells, acetaldehyde leads to the generation of DNA adducts, particularly the N^2^‐propano‐2′‐dG type which could further evolve as a wide variety of toxic ICLs, creating a cascade of genomic stress suffered by these fully differentiated, postmitotic cells. Accumulation of unrepaired DNA damage as a result of repair infidelity is one of the major drivers of cellular senescence, which occurs in these cells. Mechanistically, this could be in part attributed to the impaired folate catabolism via the DHFR enzyme, where the latter is de‐activated by the NADPH dyshomeostasis resulting from both the prioritized actions of CYP2E1 and the suppression of NADPH regeneration via the glucose‐PPP axis. Failing folate metabolism depletes SAM whilst the toxic SAH by‐product of the methionine cycle accumulates. Both would negatively impact SETD2 activities that are needed to initiate the non‐cell cycle dependent repair machinery for ICL repair amongst these post‐mitotic cells. With these pathways become dysfunctional, neurons however utilize the alternative FA‐HR axis; with a toll to re‐activating the cell cycle machinery. Instead of completing a full cell cycle, these cells however became permanently arrested by an irreversible senescent response. Their accumulation is associated with poorer cognitive and memory functions, even after a brief period of alcohol abstinence.

Our study revealed that DNA‐damage‐associated neuronal senescence response is an unexpected outcome of the altered NADPH homeostasis induced by prolonged ethanol metabolism. Glucose is the physiological fuel in neurons (Suzuki et al., 2011). Glycolysis on the one hand supplies pyruvate for energy production in the TCA cycle; on the other hand, it also contributes carbons to the PPP that supports the cytosolic NADPH production. Compromised glycolytic catabolism hijacked by the prioritized usage of ethyl alcohol not only reduces the PPP flux and NADPH production; but also deprives the existing NADPH pool via the CYP2E1 reaction. NADPH is a classic electron carrier for maintaining cellular redox homeostasis; it may also serve as a coenzyme substrate. In neurons, it is mainly for sustaining the folate and methionine one‐carbon metabolic network, which cross talks to the choice of downstream ICL repair machinery. In contrast, elevated cellular levels of SAH and Hcy are also observed, which may also contribute to vascular complications commonly found in cognitive impairment and dementia (Price et al., 2018). The resulting hypo‐glycolytic flux is another established risk of dementia, including Alzheimer's disease (Crane et al., 2013; Willette et al., 2015).

Adult neurons are typically considered permanently postmitotic, and they must constantly suppress cell cycle events (Y. Yang et al., 2003). Nevertheless, ample amount of evidence supports that these cells may re‐engage in a cell‐cycle‐like process in response to stress (Y. Yang et al., 2003). However, rather than completing a full cycle of cell division, these cells may eventually die from mitotic catastrophe or persist in the “cycling” stage in the brain for long periods of time (Y. Yang & Herrup, 2005). Here we propose these “lingering” cycling neurons are indeed engaged in a senescence response, triggered potentially by persistent repair intermediates that emerged from aberrant usage of cell cycle‐dependent HR‐mediated repair. Traditionally, HR is considered the most accurate mechanism of repair; however, the process involves sister chromatid alignment and extensive processing of the DNA which also increases the chances for the formation of complex intermediate structures (Piazza & Heyer, 2019). We speculated that these events are particularly disfavoured by non‐proliferating cells like mature neurons (von Zglinicki et al., 2021). One of the best‐known unresolved repair intermediates generated from HR is the resected DNA labelled by the single‐strand binding protein RPA without parallel recruitment of RAD51 (Mehta & Haber, 2014), which is robustly found in neurons under prolonged ethanol exposure. This is indeed equivalent to a prolonged p53‐dependent DNA damage response. Sustained expression of p53 target p21 then promotes cyclin B1 nuclear translocation, signalling a “point of no return” trigger to a senescence fate (Charrier‐Savournin et al., 2004; Feringa et al., 2018). In our study model, we believe this irreversible change in cellular status is a key contributor to behavioural deficits that persisted 20 days after the entire drinking paradigm. With young adolescent mice chosen to match up with the age when the drinking started amongst the subjects of the human single nucleus RNA‐seq dataset (Brenner et al., 2020); we realize the functional deficits observed could be in part rooted in interference of neuronal maturation during this age period (Semple et al., 2013). In contrast, our brief alcohol abstinence model may shed light, but it remains insufficient to conclude at this stage it is directly associated with accelerated cognitive and memory decline in the later stage of life. Nonetheless, a recent study, however, proposes this could potentially be the case as it is suggested that even a single dose of ethanol intoxication could lead to acute but also lasting neuronal and synaptic changes in the brain (Knabbe et al., 2022). Future in vivo studies are warranted with longer treatment and observation periods in older mice to clarify these issues.

Our analyses suggested that these cellular ageing events are metabolic consequences resulted from prolonged and prioritized ethanol oxidation, hinted the potentials of a metabolic correction strategy that aimed at addressing the root biochemical causes of disease via re‐supplementing the necessary micronutrients to regain the body's homeostatic balances (Miranda‐Massari et al., 2016). We revealed that direct brain supplementation of 5‐mTHF but not folate via the intranasal route was beneficial in preserving both cognitive and memory functions in the study model. 5‐mTHF is the bioactive form of folate, its supplement bypasses the need for processing by the NADPH‐dependent DHFR to support the folate cycle to rescue the function of the methionine synthase—the key enzyme that connects the folate and methionine cycles via receiving 1‐carbon moieties from 5‐mTHF for converting homocysteine to methionine. The proper functioning of the methionine synthase indeed also depends on the availability of its coenzyme cobalamin (i.e., Vitamin B12) and cofactor zinc (Abdel‐Azeim et al., 2011). Whilst their brain quantities remained insignificantly different in our study model (Figure S9j,k), their statuses were also taken into consideration as high folate supplementation alone in aged individuals with low body cobalamin may worsen cognitive decline (Moore et al., 2014). Apart from that, we also noticed that neurons harbouring the inactive variant of *ALDH2* did not seem to be responding to 5‐mTHF supplementation alone. In light of knowing there is an existing ALDH2 agonist Alda1 available, which activates both the wildtype ALDH2*1 and the semi‐dominant inactive ALDH2*2 variants (Perez‐Miller et al., 2010), a modified drug‐nutrient strategy which constitutes both the 5‐mTHR and Alda1 was adopted and such was able to yield better protective effect than 5‐mTHF alone.

In conclusion, our study demonstrated the molecular and cellular basis on how chronic binge‐like drinking leads to lasting impairment in cognitive and memory function, contributing to the risk of dementia in long term. The study also provides evidence showing that the 1‐carbon network is a potential therapeutic target for preventing neuronal senescence caused by excessive drinking.

## 4 METHODS

### 4.1 Study design

The objective of this study was to characterize the persistent molecular changes induced by chronic alcohol (i.e., ethyl alcohol) exposure which underlies its lasting effect on the brain physiology, thereby making itself a risk factor for age‐related dementia. Complementary transcriptomic, metabolomic and pharmacological approaches were employed to elucidate the effect of ethyl alcohol metabolism in vivo and in vitro. The effect of chronic ethyl alcohol metabolism on animal behaviour, DNA damage response and cell cycle status of neurons were evaluated in wildtype mice; forebrain neuron‐specific aldehyde dehydrogenase 2 knockout (*Aldh2* ^*−/−*^) mice, and in the same *Aldh2* ^*−/−*^ mice with ectopic re‐expression of human ALDH2*1 and ALDH2*2 variant in neurons. Human studies entailed re‐assessment of a publicly available single nuclei RNA‐sequencing dataset of human forebrain tissues from patients with alcohol use disorder and unaffected subjects.

Sample sizes for animal experiments were determined based on our previous studies. For all animal experiments involving genetic modification of *Aldh2* and quantification of the outcome measures, littermates were used as controls. For animal experiments using commercially obtained mice, grouping was randomized. Cell experiments were repeated at least three times with at least three replicates within each condition. Investigators were blinded to treatment assignments and/or sample group information wherever practical. All animal protocols were approved by the responsible authorities at the Hong Kong University of Science and Technology (HKUST) and the Chinese University of Hong Kong (CUHK).

### 4.2 Availability of data and materials

All data generated during this study have been included in the manuscript. Further data supporting the findings of this study are available from the corresponding authors on request. The original RNA sequencing dataset is now deposited in GEO Omnibus (GSE202183).

### 4.3 Reagents, RNA inferences, open reading frame plasmids

Unless otherwise specified, all chemicals and reagents were purchased from Sigma‐Aldrich. Details of antibodies, special reagents, assay kits, sequence‐based reagents and analytical software, as sequences for oligos and a list of unique reagents are provided in Table S6. Unique reagents generated in this study will be made available upon reasonable request to the lead contact with a completed Materials Transfer Agreement.

### 4.4 Animal maintenance and brain tissues harvesting

#### 4.4.1 Animal maintenance

C57BL/6J and Ca2+/calmodulin‐dependent protein kinase II alpha (CaMKIIα)‐Cre (B6.Cg‐Tg(Camk2a‐cre)T29‐1Stl/J) were obtained from the Jackson Laboratory. Aldh2‐Flox (C57BL/6‐Aldh2em1(flox)Smoc) line was obtained from the Shanghai Model Organisms. Mouse colonies were maintained and bred in the Animal and Plant Care Facility of HKUST and the Laboratory Animal Services Centre of CUHK. All animal experimental protocols were approved both by the Animal Ethics Committees at both HKUST and CUHK, and their care was in accord with both the institutional and Hong Kong guidelines.

#### 4.4.2 Brain tissue harvesting

This was performed by first anaesthetizing the mice with intraperitoneal administration of 1.25% (vol/vol) Avertin at a dosage of 30 ml/kg body weight. The heart of each mouse was then surgically exposed, the left chamber was catheterized, and the right atrium was opened. Chilled physiological saline was perfused transcardially for 3 min to remove blood from the body. After perfusion, the cranial bones were opened; cortex and cerebellum tissues were harvested, snap‐frozen in liquid nitrogen, and stored at −80°C until use.

### 4.5 Two‐bottle choice‐drinking in the dark (2BC‐DID) binge‐like drinking model

#### 4.5.1 2BC‐DID

A previously published protocol two‐bottle choice (2BC)‐Drinking in the Dark (DID) was adopted in this study (Huynh et al., 2019). The 2BC drinking, also known as free choice drinking, preference drinking or social drinking, two bottles of solution is continuously available in the home cage one of which contains water and the other contains a diluted solution of ethanol (20% v/v). Mice have constant access to both bottles, and therefore, can choose how much to drink from each bottle. Mice from the control group were given two bottles of water, mice from the ethanol group were given one bottle of 20% (v/v) ethanol (from 190 proof 95% ethanol, Decan Laboratories) solution and one bottle of water at equal volume. Bottles and mice were weighed daily and bottle positions (left/right) were alternated between each alcohol access session. As for the DID procedure, limited access to alcohol took place 3 h after entering the dark cycle, which lasted for 12 h, with bedding and food available ad libitum. The daily assessment of ethanol consumption of each mouse (g/kg), as well as ethanol preference ratio (volume of ethanol consumed/total volume liquid consumed), were performed. On the 15th alcohol‐drinking night, blood was collected from separate mice at 1, 3, 6 and 12 h after the initiation of drinking to measure blood alcohol levels. To sample BEC during drinking, mice were briefly immobilized (<1 min) in a restraint tube (Braintree Scientific, Braintree MA) and a scalpel was used to make a small nick at the end of the tail. All blood was collected in heparinized tubes and immediately centrifuged to separate and collect plasma. BAC in the collected plasma was measured via the Analox GL6 analyser (Analox Instruments, Lunenburg, MA). Mice were given 35 consecutive sessions (7 weeks) of access to alcohol in total.

#### 4.5.2 Intranasal drug delivery

Administration of 5‐mTHF alone or with ALDH2 agonist Alda1 was performed through the intranasal route everyday within 30 min after the end of the 12‐hour drinking section in the light cycle. With a firm grip on the scruff, the tip of the pipettor containing the sample was placed near the left nostril of the mouse at a 45° angle, and about 5 μl of the sample was administered to the nostril with a 2–3 s interval in between for a total of 5 μl/nostril. The mouse was held in this position for 5 s or until it regained consciousness, and then the administration step was repeated for the other nostril for a total of 10 μl/mouse. After the mouse had received all drops, animals were kept restrained on its back until the material disappeared into the nares and then returned to its cage until the entire treatment plan was finished.

Following the entire 35 sections of 2BC‐DID procedure, mice were separated into two arms—Arm #1 and #2. In Arm #1, a group of experimental and control mice were immediately tested for spatial learning and memory performance using a Morris water maze, which allows the mice to utilize distal cues to navigate from start locations around the perimeter of an open swimming arena to locate a submerged escape platform. Short‐term memory was also assessed by the Y‐maze‐based experiments with spontaneous alternation protocol. Spatial reference memory was also tested by the Y‐maze‐based novel arm entry paradigm. Depressive‐like behaviour was assessed by the force‐swimming test, and motor coordination by the rotarod test. For mice assigned in Arm #2, they were allowed to stay abstinent for 2 weeks prior to undergoing the same set of behavioural tests as mentioned above. After all the behavioural tests, all animals were sacrificed. Brain tissues were harvested for various types of analyses.

### 4.6 Animal behavioural tests

All tests were performed as previously described with slight modifications (Zhou et al., 2020). All behavioural tests were performed during the light phase of the circadian cycle between 09:00 and 17:00 in counterbalanced order across different treatment groups. We and others have previously shown that the behaviours being measured in this study are not altered by either testing time of day or single housing C57BL/6J, the wildtype background strain (Keers et al., 2012; Zhou et al., 2020). All behavioural testing began by allowing mice to habituate to the testing rooms before tests. Experiments were performed blind to the genotype and treatments when behavioural tests were carried out. With an overhead camera and the Smart 3.0 Video Tracking system (Panlab), animal behavioural tests were performed as described below:

#### 4.6.1 Morris water maze (MWM) test

The Morris water maze test was conducted as described previously (Zhou et al., 2020). Briefly, a blue circular tank (90 cm (diameter)*35 cm (high)) was filled with water (≈22°C), and a platform (10 cm in diameter) was submerged 1 cm beneath the surface of the water in a target quadrant. The walls surrounding the tank contained bright and contrasting shapes that served as reference cues. The training was conducted over six consecutive days with four trials/day using an inter‐trial interval of 1–1.5 min. Mice were placed randomly into each of the four starting locations for each of the four daily training trials. In each trial, mice swam until they found the hidden platform or were gently guided to it by the experimenter if not found within 60 s. Mice remained on the platform for 15 s before returning to the home cage. Daily data were averaged across the four trials. On day 7, a probe trial was conducted, and the hidden platform was removed, mice were placed in the pool and allowed to swim for 60 s. The time spent in each of the target quadrants and the latency time to target was recorded.

#### 4.6.2 Rotarod test

Mice were placed on a stationary rotarod (IITC Life Sciences) in a well‐lit room that was then activated and accelerated from 0 to 45 revolutions per minute over 5 min as described previously (Zhou et al., 2020). The latency of mice to fall off the rod was measured. Trials were repeated four times with intertribal intervals of 30 min over a single day.

#### 4.6.3 Forced swim test

The forced swim test was used for the evaluation of depressive‐like behaviours as described previously (Zhou et al., 2020). The tanks (15 cm (D) × 30 cm (H)) were filled with tap water set at 22°C. Mice were placed in the water and their escape‐related mobility behaviour was measured for 8 min.

#### 4.6.4 Y‐maze test

The short‐term working memory was assessed in the Y‐maze spontaneous alternation test using a grey opaque Perspex Y‐maze with three arms each containing a visual cue (arm dimensions: 15 × 10 × 10 cm) as previously described (Zhou et al., 2020). In this discrete trial procedure, there are two phases to each trial: a sample phase (the information‐gathering stage, where the animal runs to one goal arm of the maze and a memory trace of this event is formed) and a choice phase (the animal's choice between the sampled and unsampled arms may or may not be guided by the memory of recently visiting the former). The sample phase began with the placement of the mouse in the designated starting arm of the maze. The mouse could freely explore two of the three arms for 5 min. The mouse was then returned to the home cage for 30 min before the start of the choice phase. The choice phase began with the placement of the mouse in the same starting arm as the sample phase and was allowed to freely explore all three of the arms for another 5 min. An arm entry was defined as four limbs within the arm. The percentage number of alternations was calculated as the number of actual alternations divided by the maximal number of alternations (the total number of arm entries minus 2). The total number of moves was also recorded as an index of ambulatory activity.

### 4.7 Mouse cortex tissues bulk RNA sequencing

Frozen cortex tissues harvested from mice subjected to the 2BC‐DID were harvested and sent to the BGI Genomics for total RNA extraction and RNA sequencing with the DNBSET™ platform. FASTQ files obtained were subsequently analysed by the FastQC (V. 0.11.9) of Babraham Bioinformatics, serving as a quality control assessment tool for these data. After assuring the quality of the data files, genome indexes were generated using the STAR software using. These indexes were generated with the mouse annotation and mouse genome assembly provided by Gencode Release 28 (GRCm39, released in May 2021). The cleaned dataset was then aligned with the genome indexes using the STAR alignment software. FeatureCounts was then used to quantify the sequencing data. Data extraction, matrix construction and differential gene expression analysis were then performed using the DESeq2 package on R. Pathway analysis was performed with the Ingenuity IPA (Qiagen) platform and Enrich R. The original FASTQ files were deposited on GEO Omnibus (GSE202183).

### 4.8 Single‐cell RNA sequencing data mining and analysis

The original and raw single nuclei‐RNA sequencing data of the prefrontal cortex of control and AUD individuals were downloaded from the GEO database (GSE141552).

DropletUtils was then used as the first filter to remove any cells with extremely low expression in each of the samples, with the false positive rate set as 0.01. Filtered cells were then merged into an integrated dataset which was then analysed by the Seurat 4.0.5. Genes expressed in at least three nuclei were retained, and those outlier nuclei with a high ratio of mitochondrial encoded transcripts (i.e., >20%, <200 UMI) relative to the total RNA, and those potential doublets (>6000 UMI) were discarded in the subsequent analysis. The top 30 dimensions and resolution with 0.5 as input were used to build the tSNE graph and cell clusters.

Major brain cell types were annotated with well‐validated markers, these include *CAMK2A* and *GRIA1* as markers for neurons; *SLC1A2* for astrocytes; *MOBP* for oligodendrocytes; *VCAN* is for oligodendrocytes precursor cells and *P2RY12* for microglia and *FLT1* for endothelial cells.

For cell cycle gene analysis, a list of cell cycle‐related genes was downloaded from KEGG (hsa04110) and selected for targeted analysis. Their average expression in each cell cluster was obtained by the AverageExpression functions in the Seurat 4.0.5 package. Those enriched in target clusters were then clustered for pathway analysis using the EnrichR software, referencing the Reactome dataset.

The population of cell cycle re‐engaged neurons was compared between the control and AUD groups, and the significance was calculated by the Wilcoxon test.

The identification of differentially expressed genes of the targeted clusters between the control and AUD groups were calculated using the FindMarkers function in the Seuret 4.0.5 package with the cut‐off value set at p‐value <0.01 and min.pct = 0.25. Subsequent analyses were performed using the EnrichR software, referencing the Reactome dataset.

### 4.9 Quantification procedures and statistical analysis

For each experiment, no statistical methods were used to predetermine sample sizes, but our sample sizes were similar to those reported in recent publications (Chow et al., 2021; Zhou et al., 2020). Data distribution was assumed to be normal, but this was not formally tested. All samples were analysed, and the data collected was blinded to the experimental conditions. All experiments were performed on at least three independent occasions. Quantification of cellular morphology parameters was performed in a blinded manner. Analyses of the qPCR data were performed on Prism 8.0. Differences between groups were analysed using two‐tailed unpaired Student's *t*‐test (for two groups) or one‐way ANOVA (for more than three groups) for normally distributed data or using a Wilcoxon signed‐rank test for skewed data. Two‐way ANOVA was used to determine the effect of two nominal predictor variables. Pathway analysis was performed on Enrich R (https://maayanlab.cloud/Enrichr/). All statistical analyses were performed using GraphPad Prism 8.0 and the SigmaStat statistics software package. Log2FC ± 0.5 together with *p* < 0.05 were considered to indicate statistical significance.

Further information on methods is available in the Supplemental Information.

## AUTHOR CONTRIBUTIONS

Hei‐Man Chow and Ronald P. Hart conceptualized the study. Hei‐Man Chow, Jacquelyne Ka‐Li Sun, Genper Chi‐Ngai Wong, Meigui Yang, Tsun‐Ming Lau and Ronald P. Hart performed morphological analysis, biochemical and molecular assays, behavioural tests, mass spectrometry analyses, primary cultures and animal experiments. Deng Wu performed single‐cell transcriptomic analysis. Hei‐Man Chow wrote the manuscript. Hei‐Man Chow, Ronald P. Hart, Ho Yin Edwin Chan and Kin‐Ming Kwan discussed and edited the manuscript. Hei‐Man Chow supervised the project. All authors reviewed and gave final approval to the manuscript.

## CONFLICT OF INTEREST

None.

## Supporting information


AppendixS1

TableS1

TableS2

TableS3

TableS4

TableS5

TableS6

FigureS1

FigureS2

FigureS3

FigureS4

FigureS5

FigureS6

FigureS7

FigureS8

FigureS9

FigureS10

## Data Availability

The data that support the findings of this study are available from the corresponding authors upon reasonable request. Unique reagents generated in this study will be made available upon reasonable request to the lead contact with a completed Materials Transfer Agreement. The bulk RNA‐sequencing dataset is deposited in GEO Omnibus (GSE202183).
