# Supplementary material for: Chronic alcohol metabolism results in DNA repair infidelity and cell cycle‐induced senescence in neurons
Source: Aging Cell. 2023 Jan 23;22(2):e13772. doi: 10.1111/acel.13772 (PMC9924945; doi:10.1111/acel.13772)
Supplement: Supplementary file 1 — AppendixS1 [file ACEL-22-e13772-s017.docx]

**Supplementary Materials**

**Chronic alcohol metabolism results in DNA repair infidelity and cell cycle-induced senescence in neurons**

Jacquelyne Ka-Li SUN**^1^***

Deng WU^1*^

Genper Chi-Ngai WONG^1^

Tsun-Ming LAU**^1^**

Meigui YANG**^1^**

Ronald P HART**^5^**

Kin-Ming KWAN^1,2,3^

Ho Yin Edwin CHAN^1,4^

Hei-Man CHOW**^1,#^**

**Contents:**

1. **Supplementary Methods**
2. **Supplementary Figures and Legends**

**Supplementary Methods**

**Construction of the humanized *ALDH2* lentiviral plasmids**

A plasmid containing the NM_000690 isoform cDNA of human *ALDH2* was purchased from OriGene. Wild-type sequences were mutagenized to the E504K variant using the NEBaseChanger kit (New England Biolabs) with mutagenesis primers 5’-GGCATACACTaAAGTGAAAACTGTCACAGTCAAAGTG-3’ and 5’-TGCAGCCCGTACTCGCCC-3’. Mutagenesis was confirmed by sequencing. The coding sequence was extracted by PCR from both wild-type and E504K and assembled into a lentivirus backbone, driven by a human synapsin I promoter and fused with GFP at the C-terminal via a T2A “self-cleaving” linker, using the NEB HiFi Assembly Master Mix (New England Biolabs). Successful clones were confirmed by sequencing.

**Lentivirus production and transduction**

Lentivirus stocks were produced as previously described with slight modifications(Chow et al., 2019). Human embryonic kidney 293FT cells were transfected using Lipofectamine 2000 (Thermo Fisher) with the expression of two helper plasmids: psPAX2 and pMD2.G. Ten micrograms of the transfer vector, 5 µg psPAX2 and 5 µg pMD2.G of DNA were used per 10‐cm plate. Forty‐eight hours after transfection, the supernatants of four plates were pooled, centrifuged at 780 × g for 5 min, filtered through a 0.45‐µm pour size filter, and further centrifuged at 24 000 rpm for 2 h. The resulting pellet was re‐suspended in 100 µL of PBS. Lentivirus titration was performed on DIV5‐6 at titre range 107 IU/mL. In most experiments, cultures were infected overnight, rinsed twice with virus‐free medium the next morning and incubated in normal culturing medium for another 48–72 hours prior assay.

**Intracerebroventricular lentivirus injection**

The stereotactic injections of viral vectors were performed in isoflurane-anesthetized and head-fixed mice using a 10 μL Hamilton syringe. Injection volume and speed were controlled using a WPI Ultra Micro Pump. Lentiviral injections were performed with the in house packaged lentivirus carrying either the *hSyn-EGFP-ALDH2*1* or *hSyn-EGFP-ALDH2*2* constructs at titre 1.02 x 10^3^ genome copies per ml. EGFP expression at the site of injection was used to verify injection coordinates and determine the precise location of the injection. Injection coordinates at the medial prefrontal cortex were AP 1.4 mm to AP 2.8 mm, ML 0.25 mm, DV 2.3 mm (Nanoject, Neurostar). The needle was slowly withdrawn after injection. Animals were placed on a warming pad to recover and returned to the cage. Ectopic expression of genes was allowed for another 25-30 days before tissue collection and subsequent analyses.

**Mouse primary neuronal culture**

Mouse embryonic cortical neurons were isolated by standard procedures. Gravid females were killed on the 16th day of gestation and E16.5 embryos collected in ice‐cold PBS‐glucose. The cortical lobes were removed, following which the meninges were removed and the cortices were placed in 1× trypsin-EDTA solution for 10 min, with manual shaking for 5 min. After digestion, an equal volume of DMEM with 10% (vol/vol) FBS was added to inactivate the trypsin. Samples were then centrifuged at 2,500 rpm for 5 min. Supernatant was removed, followed by transferring the pellet to fresh Neurobasal medium supplemented with B‐27, penicillin/streptomycin (1×) and L‐glutamine (2 mM; GlutaMAX) prior to gentle resuspension. Tissue was triturated ten times through a 5 mL pipette and allowed to settle to the bottom of a 15‐mL conical tube. Dissociated cells in solution above the pellet were removed. Surviving cells were identified by trypan blue exclusion and counted before plating on poly‐L‐lysine‐coated (0.05 mg mL^−1^) glass coverslips. Enrichment of neuronal culture was performed using both a previously reported. 1.5 μM 5-fluoro-2’-deoxyuridine (FdU) (Sigma) was added at DIV4 and incubated for 24 hours to kill any proliferating cells in the dish. Medium containing FdU was then replaced with fresh medium without FdU (old to new complete Neurobasal medium in 1:1 ratio). Half of the medium was then replaced every 3 days for maintenance (Hui, Zhang, & Herrup, 2016). Unless otherwise specified, cells were plated in 24‐well plates at 50 000 cells per well and allowed to mature for over 7–10 days in vitro (DIV) before transfection or lentiviral transduction. For other experiments, cells were grown for a minimum of 14 days in vitro (DIV14) before any drug‐treatment experiments.

**Primary astrocyte culture**

In mouse primary astrocyte culture, we have adopted the culturing protocol developed by the Barres lab (Foo et al., 2011; Zhang et al., 2016). For mouse astrocytes cultures, purification of ultrapure brain cortex astrocytes from postnatal pups (P1-2), the experimental step contains three major steps, including tissue dissociation, additional myelin removal, and astrocyte isolation as previously reported (Batiuk et al., 2017). Brain cortical tissue was dissociated using the Neural Tissue Dissociation Kit P (papain) (Miltenyi Biotec.). Following enzymatic digestion, cells were released using mechanical trituration with 10 mL serological pipettes (three rounds of ten strokes each). Freshly dissociated cells were then passed through a 20‐µm nylon net filter to remove remaining tissue clumps. Myelin removal using equilibrium density centrifugation was then performed. With 90% Percoll PLUS in 1 × Hank's balanced salt solution (HBSS) with calcium and magnesium added to the cell suspension, a final Percoll concentration of 24% was achieved. DNase I was then added (1250 units per 10 mL of suspension), and the suspension was mixed and then spun down at 300 × g for 11 min at room temperature in a Hettich 320R universal centrifuge. The cell‐containing pellet was resuspended in 0.5% BSA in PBS without calcium and magnesium (Thermo Fisher Scientific). Additional myelin removal step was performed with Myelin Removal Beads II (Miltenyi Biotec.) according to the manufacturer's instructions along with the LS magnetic column (Miltenyi Biotec.). The flow‐through was subsequently collected and made to proceed to astrocyte isolation with the ACSA‐2 kit (Miltenyi Biotec.) according to the standard protocol, using two runs of enrichment on consecutive MS columns (Miltenyi Biotec.). Portion of enriched astrocytes were subjected purity testing while the rest was used for culture in astrocyte growth media for initial proliferation phase until confluence (around 3 days), followed by switching to astrocyte base media for differentiation (another 4-5 days) prior applying any treatments. Both astrocyte growth media and base media were prepared freshly in the same formula as previously reported (Foo et al., 2011).

**Chronic intermittent ethanol (CIE) exposure**

CIE exposure was performed for various time courses and initiated at around DIV8-9. Briefly, conditioned neuronal culture medium was used for the CIE exposure paradigm by supplementing ethanol to a final concentration at 20 mM. The control plates were fed with identical medium but without ethanol. The 95% ethanol purchased from 190 proof Decan Laboratories was used. The primary neurons were fed with the above medium which being refreshed in every 24 h and the entire treatment lasted for different durations as indicated in the corresponding figures. The so-called “compensating system” designed to prevent ethanol evaporation in the culture dishes consisted of saturation of the atmosphere over the dishes by placing them inside an expanded polystyrene container (250 x 140 x 180 mm) with an open pan containing 50 mM ethanol (4.125 mmoles) in aqueous solution (Eysseric et al., 1997). The container was closed but not sealed in such it did not prevent the passage of CO2 and air through the polystyrene when placed in the incubator. Culture dishes were placed directly inside the polystyrene box and a persistent exposure to 20 mM ethanol was maintained by daily refreshment with new conditioned medium with 20 mM ethanol. Changes in ethanol concentration in the medium was examined with the Analox GL6 analyser (Analox Instruments, Lunenburg, MA).

**Cell culture and transfection**

Immortalized human embryonic kidney cells (HEK293FT cell line, Thermo Fisher Scientific) were cultured in DMEM/10% (vol/vol) foetal bovine serum/penicillin–streptomycin medium (Gibco). Primary neurons were isolated from embryonic day 16 embryos of C57BL/6J mice and cultured as described above. DNA constructs were transfected with Lipofectamine 2000 or Lipofectamine LTX with Plus Reagent into HEK293FT cells and primary neuronal cultures, respectively. Following the manufacturer’s protocol, 6 hours after transfection, cells were refreshed with fresh culture medium and further incubated for 48–72 hours to allow recovery and ectopic expression.

**Glycolysis stress test**

The XF Glycolysis Stress Test (Seahorse Bioscience, Agilent) was used to assess glycolysis function in cells, which was conducted using the XFe24 Analyzer. By directly measuring ECAR, the kit provided a standard method to assess the following key parameters of glycolysis flux: glycolysis, glycolytic capacity and glycolytic reserve, in addition to non-glycolytic acidification. Cells were seeded at a density of 45,000 cells/well. Cells were first incubated in pyruvate-free glycolytic assay medium for 1 hour prior to the first injection of a saturated concentration of glucose (final concentration: 10mM). The cells catabolize glucose into pyruvate via the glycolysis pathway, producing ATP, nicotinamide-adenine dinucleotide (reduced form), water and protons. The discharge of protons into surrounding medium leads to a sudden increase in ECAR, which was used to define the basal glycolytic capacity. The second injection was oligomycin (final concentration: 1 µM), which may divert energy production to glycolysis by restricting mitochondrial ATP production. Consequently, the sharp increase in ECAR indicates the level of glycolytic capacity. The final injection was 2-deoxy-glucose [2-DG; final concentration: 50 mM], which is a glucose analogue that inhibits glycolysis through competitive binding to glucose hexokinase; the first enzyme in the glycolytic pathway. The resulting decrease in ECAR confirmed that the ECAR produced in the experiment was caused by glycolysis. The gap between glycolytic capacity and glycolysis was defined as the glycolytic reserve. The ECAR prior to glucose injection is referred to as non-glycolytic acidification and may occur due to additional processes in the cell.

**Quantitative RT-PCR (qPCR) analysis**

Total cellular RNA was purified from brain tissues or cultured cells using RNease kit (Qiagen) following the manufacturer’s protocol. For quantitative real-time PCR (qPCR), RNA was reverse-transcribed using the High-Capacity cDNA Reverse Transcription Kit (Applied Biosystems) according to the manufacturer’s instructions. The resulting cDNA was analysed by qRT-PCR using SYBR Green PCR Master Mix (Applied Biosystems). All reactions were performed in a Roche LightCycler (LC) 480 instrument using the following protocol: pre-incubation at 95^o^C for 15 min (1 cycle); denaturation at 94^o^C for 15 seconds, annealing and extension at 55^o^C for 30 seconds (40 cycles), melting at 95^o^C for 5 seconds, 65^o^C for 60 seconds and 95^o^C continues (1 cycle) followed by cooling at 40^o^C for 30 seconds. The specificity of the primers was confirmed by observing a single melting point peak. qPCR efficiency was calculated from the slope between 95 and 105% with co-efficiency of reaction R^2^=0.98-0.99. A total of 7-9 biological replicates x at least 3 technical replicates were performed for each treatment group. Normalization of results to stabilized expressed reference gene *Rpl13* in the brain was performed *(Rydbirk et al., 2016)*. Data was analysed using the comparative Ct method (ΔΔCt method).

**SDS-PAGE-Western Blotting**

Isolated brain tissues or cell pellets were homogenized in RIPA buffer (Millipore) with 1X complete protease inhibitor mixture (Roche) and 1X PhosSTOP phosphatase inhibitor mixture (Roche) on ice then centrifuged for 10 minutes at 18,400 x g to remove large debris. The protein concentration of the supernatant was determined by Bradford Assay (Bio-Rad). For SDS-PAGE (polyacrylamide gel electrophoresis), 100 µg of proteins derived from cell or tissue lysates were prepared in 5X sample buffer [10% w/v SDS; 10mM beta-mercaptoethanol; 20% v/v glycerol; 0.2M Tris-HCl, pH6.8; 0.05% Bromophenol Blue]. With a Bio-Rad system, separating gels of different acrylamide percentages (6%-15%) were prepared with the following components in double distilled water: acrylamide/bis-acrylamide (30%/0.8% w/v); 1.5M Tris (pH=8.8); 10% (w/v) SDS; 10% (w/v) ammonium persulfate and TEMED) and a 5% stacking gel (5ml prep: 2.975ml Water; 1.25ml 0.5M Tris-HCl, pH6.8; 0.05 ml 10% (w/v) SDS; 0.67ml acrylamide/bis-acrylamide (30%/0.8% w/v); 0.05ml 10% (w/v) ammonium persulfate and 0.005ml TEMED) were prepared. Samples were run in SDS-containing running buffer [25mM Tris-HCl; 200mM Glycine and 0.1% (w/v) SDS] until the dye front and the protein marker reached the foot of the glass plate. Standard immune-blotting procedures were used, which include protein transfer to polyvinylidene difluoride (PVDF) membranes, blocking with non-fat milk, and incubation with primary and secondary antibody, followed by visualization with the SuperSignal West Dura/Femto Chemiluminescent Substrate (ThermoFisher Scientific).

**Immunocytochemistry and immunohistochemistry**

For immunocytochemistry, primary neuronal cultures were grown on 13-mm coverslips in 24-well plates, whereas for immunohistochemistry, 10 µm cryo-section of frozen mouse brains were used. Samples were fixed with fresh 4% (wt/vol) paraformaldehyde (Sigma-Aldrich) for 10 min, washed and followed by permeabilization with 0.3% Triton-X100 in PBS for 10 min. After blocking with 5% (wt/vol) BSA in PBS for 1 h, primary antibodies were added and incubated overnight at 4^o^C. The following day, coverslips were washed three times (10 min each) with PBS. After rinsing, secondary antibodies were applied for 1 h at room temperature followed by three additional washes with PBS. The coverslips were then inverted and mounted on glass slides with ProLong Gold Antifade Reagent (Life Technologies). Immuno-florescence was analysed, and Z-stack maximum projected images were photographed using TCS SP8 confocal microscope (Leica Microsystems Inc.)

**Senescence-associated β-galactosidase assay with immunostaining**

Senescence β-galactosidase staining was performed as we previously reported (Chow et al., 2019) with a colorimetric kit according to the manufacturer’s protocol. In brief, 50,000 DIV14 neurons seeded on 13-mm poly-L-lysine-coated glass coverslips were washed with PBS and fixed with neutral-buffered 4% formaldehyde for 5 min at room temperature. Fixed cells were washed twice with PBS before incubation in SA-β-gal staining solution with an uniform pH = 7-7.2 at 37 °C. Using the cerebellar Purkinje neurons on the same brain section as reference, our incubations were stopped at 6 ± 0.5 h when these huge neighbouring cells had not yet developed any β-gal signals while signals started appearing in the targeted forebrain brain regions.

After incubation, samples were washed twice with PBS, observed and the numbers of blue SA-β-gal-positive cells were quantified using a microscope. For staining of tissue samples, frozen specimens sectioned at 10-µm thick were briefly fixed in 1% formaldehyde for 1 min, then staining was performed using the same procedures as for the cultured cells. Caution was taken in determining the incubation time in brain sections as subpopulations of neurons in the brain will sometimes develop false-positive signals if the incubation time extends beyond 12 hour or overnight.

Immunostaining was sometimes performed after the SA-β-gal staining procedures. In brief, cells or sections were washed twice in PBS after the blue colour was developed, then they were permeabilized with 0.5% Triton X-100 in PBS for 5 minutes at room temperature. After that, blocking with 0.5% BSA in PBS was performed for 1 hour, and primary antibody incubation in 0.5% BSA was allowed for another 3 h at room temperature. Once this incubation step was completed, samples were washed three times with PBS (10 minutes each) and incubated with secondary antibodies in 0.5% BSA for 1 h at room temperature. The washing step was then repeated, followed by nuclei staining with 1 µg ml–1 4,6-diamidino-2-phenylindole (DAPI) solution for 5 min. Samples were washed twice in PBS, mounted, and observed under microscope.

**Sudan Black B Staining**

Sudan Black B staining modified from Evangelou and Gorgoulis (Evangelou & Gorgoulis, 2017) was conducted to visualise lipofuscin, aggregates of lipid and protein indicative of cell senescence. In brief, 0.7% Sudan Black B in 70% ethanol solution was prepared and filtered. Cells were first dehydrated in 70% ethanol for 2 minutes and then stained overnight at room temperature. After this, the coverslips were washed in 50% ethanol solution until a clear background was obtained for subsequent brightfield imaging analysis.

**Colorimetry assay**

Cellular or tissue contents of Vitamin B12 (Novusbio, NBP2-59958), S-adenosyl Methionine (SAM) (abbexa, abx156749), S-adenosylhomocysteine (SAH) (Cell Biolabs, MET-5151) and 5-Methyltetrahydrofolate (5-mTHF) (Biomatik, EKU02032) were evaluated by commercially available ELISA kits following the manufacturers’ instructions. For cellular or tissue levels of Zinc 2+ ions (Abcam, ab102507), NADPH and NADP+ (BioVision, K347), acetaldehyde (BioAssay Systems, EACT-100), acetate (Abnova, KA3764) and dihydrofolate reductase assay (BioVision, K246) were evaluated by commercially available colorimetric assay kits following the manufacturers’ instructions.

**Untargeted metabolome analysis by capillary electrophoresis time-of-flight mass spectrometry (CE-TOFMS) and liquid chromatography (LC)-TOFMS**

Metabolome analyses was performed in mouse frontal cortex tissue, primary neuron and astrocyte cultures using CE-TOFMS for both cationic and anionic metabolites on the basis of service purchased from Human Metabolome Technologies’ standard library. Samples were sent to HMT where their weight were first measured. For CE-TOFMS preparation, samples were mixed with 1,500 µl of 50% acetonitrile in water (v/v) containing internal standards (10 µM) and homogenized by a homogenizer (1,500 rpm, 120 sec x 1 times). The supernatant (400 µl) was then filtrated through 5-kDa cut off filter (ULTRAFREE-MC-PLHCC, HMT) to remove macromolecules. The filtrate was centrifugally concentrated and resuspended in 50 µl of ultrapure water immediately before measurement. Whereas for LC-TOFMS preparation, weighted samples were mixed with 300 µl of 1% formic acid in acetonitrile (v/v) containing internal standards (10 µM) and homogenized by a homogenizer (1,500 rpm, 120 sec x 2 times). The mixture was yet again homogenized after adding 100 µl of Milli-Q water and then centrifuged (2,300 x g, 4^o^C, 5 min). After the supernatant was collected, 300 µl of 1 % formic acid in acetonitrile (v/v) and 100 µl of MilliQ-water were added to the precipitation. The homogenization and centrifugation were performed as described previously, and the supernatant was mixed with previously collected one. The mixed supernatant was filtrated through 3-kDA cut-off filter (NANOCEP 3K OMEGA, PALL Corporation, Michigan, USA) to remove proteins and far filtrated through column (Hybrid SPE phospholipid 55261-U, Supelco, Bellefonte, PA, USA) to remove phospholipids. The filtrate was desiccated and resuspended in 200 µl of 50% isopropanol in Milli-Q water (v/v) immediately before the measurement.

*CE-TOFMS measurement:* The compounds were measured in the Cation and Anion modes of CE-TOFMS based metabolome analysis in the following conditions as previous reported (Chow et al., 2021). Samples were diluted in 2 folds for measurement, to improve analysis qualities of the CE-MS analysis.

Peaks detected in both CE-TOFMS and LC-TOFMS were extracted using automatic integration software (MasterHands ver. 2.17.1.11 developed at Keio University) in order to obtain peak information including m/z, migration time (MT) in CE, retention time (RT) in LC, and peak area. The peak area was then converted to relative peak area by the following equation. The peak detection limited was determined based on signal-noise ratio = 3.

**Relative Peak Area = Metabolite Peak Area / Internal Standard Peak Area x Sample Amount**

Putative metabolites were then assigned from HMT’s standard library and Known-Unknown peak library on the basis of m/z and MT or RT. The tolerance was ± 0.5 min in MT and ± 0.3 min in RT, ± 10 ppm (CE-TOFMS) and ± 25 ppm (LC-TOFMS) in m/z. If several peaks were assigned the same candidate, the candidate was given the branch number.

**Mass error (ppm) = (Measured Value – Theoretical Value) / Measured Value x 10^6^**

Subsequent absolute quantification was performed in target metabolites. All the metabolite concentrations were calculated by normalizing the peak area of each metabolite with respect to the area of the internal standard and by using standard curves, which were obtained by single-point (100 µM or 50 µM) calibrations. Significantly changed metabolites (with Log_2_FC ± 0.5; P<0.05) were enriched and analysed by the Metabolite Set Enrichment Analysis (MESA) or the Joint Pathway Analysis module (with KEGG metabolic gene expression data) on MetaboAnalyst (<https://www.metaboanalyst.ca/MetaboAnalyst/ModuleView.xhtml> ).

**Stable-isotope labelled glucose and ethanol metabolite tracing**

Metabolic fate and competitive metabolic flux of glucose and ethanol were performed. In primary neurons, glucose-^13^C_6_ isotope alone or simultaneously with Ethanol-^13^C_2_,1,1,2,2,2-d_5_ incubation for 2 hours was first performed, followed by tracing by the capillary electrophoresis-time of flight mass spectrometer (CE‐TOF/MS). DIV12-14 neurons were incubated in the glucose-free medium supplemented with glucose-^13^C_6_ isotope (Cambridge Isotope, CLM-1396) alone or also in the presence of Ethanol-^13^C_2_,1,1,2,2,2-d_5_ isotope (Sigma-Aldrich, 682586). Cell lysates were collected at 2-h post incubation by being washed twice with 10 mL of 5% mannitol aqueous solution, and subsequently incubated with 1 mL of methanol containing 25 µm internal standards (methionine sulfone, 2‐(N‐morpholino) ‐ethanesulfonic acid (MES) and D‐camphor‐10‐sulfonic acid) for 10 min. Four hundred microliters of the extracts were mixed with 200 µL Milli‐Q water and 400 µL chloroform and centrifuged at 10 000 g for 3 min at 4 °C. Subsequently, 400 µL of the aqueous solution was centrifugally filtered through a 5‐kDa cut‐off filter to remove proteins. The filtrate was centrifugally concentrated and dissolved in 50 µL of Milli‐Q water that contained reference compounds (200 µm each of 3‐aminopyrrolidine and trimesate) immediately before metabolome analysis.

The relative concentrations of all the charged metabolites in samples were measured by CE‐TOFMS, following the methods as previously reported (Soga et al., 2006). In brief, a fused silica capillary (50 µm internal diameter × 100 cm) was used with 1 m formic acid as the electrolyte. Methanol: water (50% v/v) containing 0.1 µm hexakis (2,2‐difluoroethoxy) phosphazene was delivered as the sheath liquid at 10 µL min−1. Electrospray ionization (ESI)‐TOFMS was performed in positive‐ion mode, and the capillary voltage was set to 4 kV. Automatic recalibration of each acquired spectrum was achieved using the masses of the reference standards. [(13C isotopic ion of a protonated methanol dimer (2 MeOH + H)]^+^, m/z 66.0632) and ([hexakis (2,2‐difluoroethoxy) phosphazene + H]+, m/z 622.0290). Quantification was performed by comparing peak areas to calibration curves generated using internal standardization techniques with methionine sulfone. The other conditions were identical to those described previously (Soga et al., 2006). To analyze anionic metabolites, a commercially available COSMO (+) (chemically coated with cationic polymer) capillary (50 µm internal diameter × 105 cm) (Nacalai Tesque) was used with a 50 mm ammonium acetate solution (pH 8.5) as the electrolyte. Methanol: 5 mM ammonium acetate (50% v/v) containing 0.1 µm hexakis (2,2‐difluoroethoxy) phosphazene was delivered as the sheath liquid at 10 µL min−1. ESI‐TOFMS was performed in negative ion mode, and the capillary voltage was set to 3.5 kV. For anion analysis, trimesate and CAS were used as the reference and the internal standards, respectively. The other conditions were identical to those described previously (Soga et al., 2009). MPE of isotopes, an index of isotopic enrichment of metabolites, was calculated as the percent of all atoms within the metabolite pool that are labelled according to the established formula (Green et al., 2016).

**DNA adducts detection**

All of the chemicals used were of the highest purity grade commercially available. This analysis involves a number of key steps as listed below:

1. DNA extraction—DNA was isolated by the modified chaotropic NaI method, as previously described (Wang, Hirayasu, Ishizawa, & Kobayashi, 1994). Briefly, the tissues (500 mg) or the cellular pellets (3 × 108 cells) were homogenized in 10 mL of a lysis solution (320 mM sucrose, 5 mM MgCl2, 10 mM Tris-HCl, 0.1 mM desferroxamine, and 1% (v/v) Triton X-100 at pH 7.5). After centrifugation at 1500g for 10 min, the pellets were resuspended in 10 mL of the lysis solution and centrifuged one more time at 1500g for 10 min. The pellets were then suspended in 6 mL of 10 mM Tris-HCl buffer (pH 8.0) containing 5 mM EDTA, 0.15 mM desferroxamine, and 350 μL of 10% SDS. The enzymes RNase A (30 μL, 10 mg/mL) and RNase T1 (4 μL, 20 U/μL) in 10 mM Tris-HCl buffer (pH 7.4) containing 1 mM EDTA and 2.5 mM desferroxamine were added, and the reaction mixture was incubated at 37 °C. After 1 h, 300 μL of proteinase K (20 mg/mL) was added, and the reaction was incubated at 37 °C for 1 h. After centrifugation at 5000g for 15 min, the liquid phase was collected, 1 mL of a solution containing 7.6 M NaI, 40 mM Tris-HCl (pH 8), 20 mM EDTA, and 0.3 mM desferroxamine was added, and then 5 mL of isopropanol was added. The contents of the tube were mixed well by inversion until a white precipitate appeared. The precipitate was collected by centrifugation at 5000g for 15 min, washed with 5 mL of 60% isopropanol, centrifuged at 5000g for 15 min, washed with an additional 5 mL of 70% ethanol, and centrifuged at 5000g for 15 min. The DNA pellet was solubilized in 500 μL of desferroxamine (0.1 mM). The DNA concentration was measured spectrophotometrically at 260 nm.

2. Enzymatic hydrolysis of DNA—sodium acetate buffer (1 M, pH 5, 4 μL) and 33 fmol of [^15^N_5_]-1,N2-εdGuo were added to an aliquot of a 0.1 mM desferroxamine solution containing 200 μg of DNA. The DNA was then digested with 2 units of nuclease P1 at 37 °C for 30 min. Tris-HCl buffer (1 M, pH 7.4, 8 μL), 8 μL of phosphatase buffer, and 6 units of alkaline phosphatase were then added for an additional 1 h of incubation at 37 °C. The final volume of the solution was adjusted to 200 μL with water before the second incubation. The enzymes were precipitated by centrifugation at 5000g for 3 min, and the resulting aqueous layer was subjected to HPLC/ESI/MS-MS analysis (100 μL of the DNA solution/injection). The amounts of the reagents and labelled internal standards were proportionally adjusted for hydrolysis and analysis of other DNA quantities.

3. Synthesis of the 1, N^2^-etheno-2’-deoxyguanosine unlabelled standard—The 1, N^2^-εdGuo unlabelled standard was obtained by reacting dGuo with chloroacetaldehyde with subsequent purification by HPLC, as described previously. The identity of the compound was confirmed by ESI/MS and 1H NMR.

4. Synthesis of the [^15^N_5_]-1,N^2^-etheno-2’-deoxyguanosine Internal standard—[^15^N_5_]-1,N2-εdGuo was obtained by reacting [^15^N_5_]-dGuo with chloroacetaldehyde with subsequent purification by HPLC, as described previously. The identity of the [^15^N_5_]-adduct was confirmed by mass spectrometry analysis.

5. Synthesis of the 1,N^2^-Propano-2’-deoxyguanosine (6R,8R) and (6S,8S) Unlabelled Standards— dGuo (25 μmol) was dissolved in 2 mL of phosphate buffer (50 mM, pH 7.5) containing 1 mmol of acetaldehyde and 0.05 mmol of lysine (as a catalyst for adduct formation). The solution was mixed at 500 rpm at 37 °C for 12 h. The adducts were purified by HPLC (Shimadzu, Kyoto, Japan) with a Luna C18(2) analytical column (250 mm ×4.6 mm i.d., 5 μm, Phenomenex, Torrance, CA). The following water/acetonitrile gradient method was used: from 0 to 30 min, 0 to 8% acetonitrile and 0.8 to 0.5 mL/min; from 30 to 50 min, 8 to 15% acetonitrile and 0.5 to 0.6 mL/min; from 50 to 60 min, 15 to 50% acetonitrile, 0.6 to 0.8 mL/min; from 60 to 65 min, 50 to 0% acetonitrile and 0.8 mL/min; and from 65 to 70 min, 0% acetonitrile and 0.8 mL/min. The identities of the two diastereomeric products were confirmed by the following spectroscopic features: UV λmax 260 nm, ε = 15600 M−1 cm−1 (6S,8S) and ε = 15700 M−1 cm−1 (6R,8R) (SPD-E10A/VP Shimadzu). ESI/MS: m/z 338 ([M + H − 2-d-erythro-pentose]^+^), 222 ([M + H]+). 1H, 1H-1H COSY NMR (D2O) (Bruker DRX 500 MHz).

6. High-Performance Liquid Chromatography/Electrospray Ionization Tandem Mass Spectrometry (HPLC/ESI/MS-MS)—Applied Biosystems, Foster City, CA). The 1, N2-εdGuo and 1, N2-propanodGuo adducts in the DNA samples were detected by multiple reaction monitoring (MRM). Agilent HPLC system consisting of an autosampler (1200 High performance), a column oven at 18 °C (1200 G1216B), an automated switching valve, a 1200 Binary Pump SL, a 1200 Isocratic Pump SL, and a UV detector (1200 DAD G1315C) were used for sample injection and clean-up of the analytical column (Luna C18(2), 250 mm ×4.6 mm i.d., 5 μm, Phenomenex, Torrance, CA). The adduct was eluted from this column with a gradient of water and acetonitrile containing 0.1% formic acid with the following method: from 0 to 10 min, 10 to 40% acetonitrile and 0.65 to 0.2 mL/min; from 10 to 16 min, 40 to 30% acetonitrile and 0.2 mL/min; from 16 to 20 min, 30 to 60% acetonitrile and 0.2 mL/min; from 20 to 21 min, 60 to 40% acetonitrile and 0.2 mL/min; from 21 to 22 min, 40 to 90% acetonitrile and 0.2 to 0.65 mL/min; from 22 to 26 min, 90% acetonitrile and 0.65 mL/min; from 26 to 27 min, 90 to 10% acetonitrile and 0.65 mL/min; from 27 to 32 min, 10% acetonitrile and 0.65 mL/min.

An isocratic pump was used to simultaneously load a second column (Eclipse XDB-C18, 150 mm × 4.6 mm i.d., 5 μm, Agilent) at 0.2 mL/min with a solution of 60:40 water/acetonitrile with 0.1% formic acid and maintained a constant flow of the mobile phase to the mass spectrometer during the analysis. The position of the switching valve was changed twice: at 13 min to allow the eluent from the first column to enter the second column and at 25 min to permit the first column to be washed while the adduct was eluted through the second column to the mass spectrometer. The total time spent on this analysis was 32 min.

The DNA hydrolysates containing 32.5-33.1 fmol of the [^15^N_5_]-1,N2-εdGuo and [^15^N_5_]-1,N2-propanodGuo internal standards were injected into the system described above. The [M + H]+ ions corresponding to the m/z values 292/176 (1,N2-εdGuo), 297/181 ([^15^N_5_]-1,N2-εdGuo) and 338/222 (1,N2-propanodGuo), 343/227([^15^N_5_]-1,N2-propanodGuo) were monitored with a dwell time of 200 ms.

All of the parameters of the mass spectrometer were adjusted for acquisition of the best [M + H]+/[M + H − 2-d-erythro-pentose]+ transition. The curtain gas was adjusted to 25 psi, the source temperature was held at 450 °C, the nebulizer and auxiliary gas were maintained at 60 psi, the Turbo Ion Spray voltage was 5500 V, the collision gas was set on high, the interface heater was held at 100 °C, and the entrance potential was set to 10 V. For the 338/222 and 343/227 transitions, the following were selected: collision energy, 19 V; collision cell exit, 20 V; and declastering potential, 51 V. For the 292/176 and 297/181 transitions were selected 17 V of collision energy, 16 V of collision cell exit, and 41 V of declastering potential. The data were processed using Analyst 1.4.2 software.

**DNA crosslink detection**

This procedure was performed as previously reported (Hu, Chang, Cooke, & Chao, 2019) and generally involved the following key steps.

1. DNA purification and enzymatic hydrolysis—DNA from cells or tissues was precipitated and recovered using a solution of NaI, 2-prorano, and ethanol, as described above. The DNA pellet obtained was dissolved in 500 μL of 0.1 mM DFO solution, to a DNA concentration of ~500 μg/mL. The DNA recovery was nearly 100%, as determined by the dG content using LC-MS/MS. For DNA enzymatic hydrolysis, a previously reported three enzyme combination protocol (nuclease P1, alkaline phosphatase and snake venom phosphodiesterase I) was employed to selectively release the ICLs as modified dinucleosides.

2. Liquid chromatography-high resolution mass spectrometry—The DNA crosslinks was separated by a reversed-phase HPLC system (Waters, Acquity UPLC, Milford, MA, USA) using an Inertsil ODS-3 C18 column (150 x 2.1 mm i.d., 5 µm, GL Sciences, Tokyo, Japan). The LC conditions were as follow: mobile phase A was 1 mM aqueous ammonium acetate (AA) solution, and mobile phase B was 100% MeOH containing 0.1% (v/v) formic acid (FA). A linear gradient elusion was performed as follows: 0.5% of mobile phase B at 0-1 min, 30% of mobile phase B at 29 min, 99.5% of mobile phase B at 46.5 min with a 5 min hold, and then a 8.5 min re-equilibration to the initial condition. The eluents were diverted to waste for the first 8.5 min of the run to remove salts and avoid contamination of the MS system with the matrix. The flow rate was 0.25 mL/min and the total run time was 60 min. The injection volume was 15 µl. Mass spectrometry analysis was performed on an LTQ Orbitrap Fourier transform mass spectrometry (FTMS) instrument (LTQ Orbitrap Elite, Thermo Fisher Scientific, MA, USA) equipped with a HESI-II electrospray source, operated in positive ion mode. The source voltage was 3.5 kV. Both the heater and capillary temperatures were 200 ^o^C. The gas settling of sheath gas, auxiliary gas, and sweep gas were 30, 15 and 1 arbitrary unit, respectively. The S-lens level was set at 40%. Data acquisition and processing were conducted by Xcalibur software 2.2 (Thermo Fisher Scientific).

3. Data-dependent neutral loss-driven MS3 (DD-NL-MS^3^) acquisition and analysis—Followed by crosslink separation, data-dependent neutral loss-driven MS^3^ (DD-NL-MS^3^) was initiated with repeated full scan detection in the FTMS, followed by MS^2^ acquisition and constant neutral loss triggering of MS^3^ fragmentation. The FTMS full scan ranged from m/z 220 to 800 and was performed in the Orbitrap at a resolution of 60,000 with automatic gain control (AGC) of 1 x 10^6^ and a maximum ion injection time of 150ms. The three most intense full scan ions together with the ion intensity greater than 7,500 from the MS^1^ full scan spectra were selected and fragmented in the ion trap (MS^2^ fragmentation by collision-induced dissociation, CID), at a resolution of 15,000. Normal 2’-deoxyribonucleosides and their ion adducts (e.g., [M + Na]+, [M + K]+, [M + NH3]+, and [M + H + H_2_O]+) were excluded from the MS^2^ fragmentation. The MS^2^ fragmentation parameters were as follows: the ion injection time was 50 ms, the isolation mass width was 2 amu, the normalized collision energy (NCE) was set at 40% and the AGC was 5 x 10^4^. Data-dependent acquisition parameters were as follows: dynamic exclusion repeat count of one, repeat duration of 30 s, exclusion list size of 250, exclusion time of 9 s, and exclusion mass width of ± 5 ppm. MS^3^ fragmentation utilizing higher collision energy dissociation (HCD), using a NCE of 50%, was triggered if a neutral loss of two dR moieties (232.0948 amu ± 5 ppm) from the parent ion was detected, and one of the 10 most intense product ions from the MS^2^, giving a minimal signal of 250, was observed. Except for NCE, other MS^3^ fragmentation parameters (i.e., resolution, ion injection time, isolation mass width, and AGC value) were the same as those used for the MS^2^ fragmentation event, as described above. All spectra were acquired using two common background ion signals, including m/z at 391.284 [bis(2-ethylhexyl) phthalate] and m/z at 445.120 (dodecamethylcyclohexasiloxane) as lock masses to ensure mass accuracy.

**Supplementary Figures and Legends:**


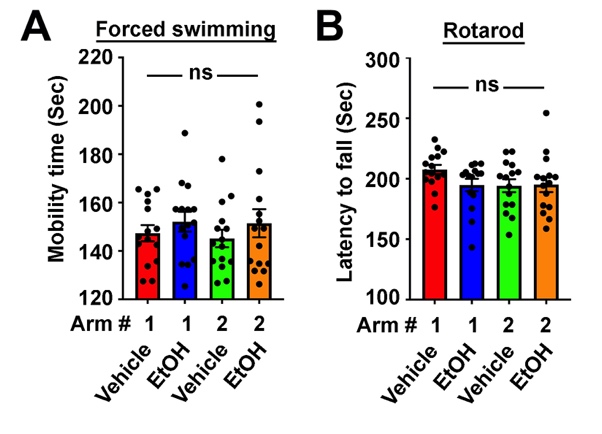


**Supplementary Figure 1. Depressive-like behaviour and motor coordination are not chronically affected upon binge-like drinking.** Mobility time of the forced swimming paradigm was quantified (N=15, ns=non-significant, one-way ANOVA). (P) Latency to fall in the rotarod paradigm was quantified (N=15, ns=non-significant, one-way ANOVA). Values represent the mean ± SEM.


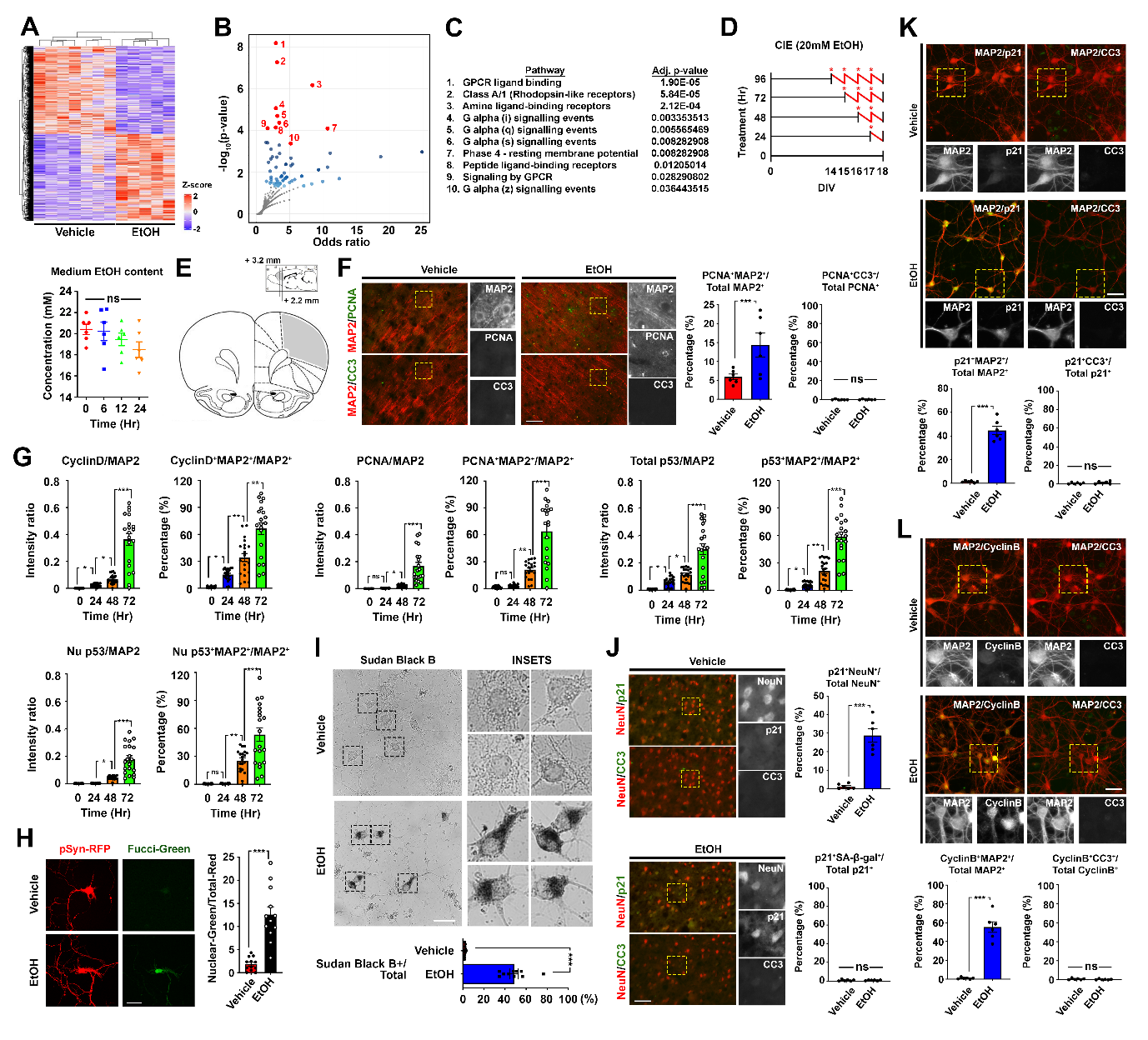


**Supplementary Figure 2. Chronic ethanol exposure promotes neuronal cell cycle re-entry and senescence.** (A) Heatmap illustrates the transcriptomic differences in cortical tissues harvested from vehicle or ethanol-exposed animals. (B-C) With the Reactome pathway database, significantly downregulated genes were clustered, the (B) volcano plot indicated the odds ratio and significance of the enriched pathways. (C) Top 10 pathways were listed out. (D) Schematics of the chronic intermittent ethanol (CIE) exposure paradigm, 20mM EtOH was refreshed along with new conditioned medium in every 24 hours. Histogram illustrated the rate of change in ethanol in medium over 24-hour period of time (N=6, ns=non-significant, one-way ANOVA). (E) Schematic brain map illustrated the motor-and somatosensory- cortex regions of the prefrontal lobe were the locations where majority of senescent neurons were identified from the immunohistochemistry analyses. (F) Representative immunohistochemistry images revealing PCNA positive neurons were negative for apoptotic cleaved caspase-3
(CC3) signals. Quantifications were shown (N=6, ***P<0.0001, ns=non-significant, two-tailed unpaired t-test, , scale bar: 200μm). (G) Quantifications on the proportion of primary neurons labelled with different cell cycle-related markers in Figure 2F upon the CIE-treatment for different courses (N=20, ***P<0.0001, **P<0.001, *P<0.01, one-way ANOVA). (H) Representative images of nuclear Fucci-Green signals in primary neuronal cultures subjected to the CIE treatment for 72 hours (N=12, ***P<0.0001, two-tailed unpaired t-test, scale bar: 20μm). (I) Representative images of Sudan Black B signals in primary neuronal culture subjected to CIE paradigm for 72 hours (N=10, ***P<0.0001, two-tailed unpaired t-test, scale bar: 100μm). (J) Representative immunohistochemistry images revealing p21 positive neurons were negative for apoptotic CC3 signals. Quantifications were shown (N=6, ***P<0.0001, ns=non-significant, two-tailed unpaired t-test, , scale bar: 200μm). (K-L) Representative immunocytochemistry images of p21, cyclin B and CC3 signals in primary neurons subjected to 72 hours of CIE paradigm. Quantifications were shown (N=6, ***P<0.0001, ns=non-significant, two-tailed unpaired t-test, , scale bar: 100μm). Values represent the mean ± SEM.


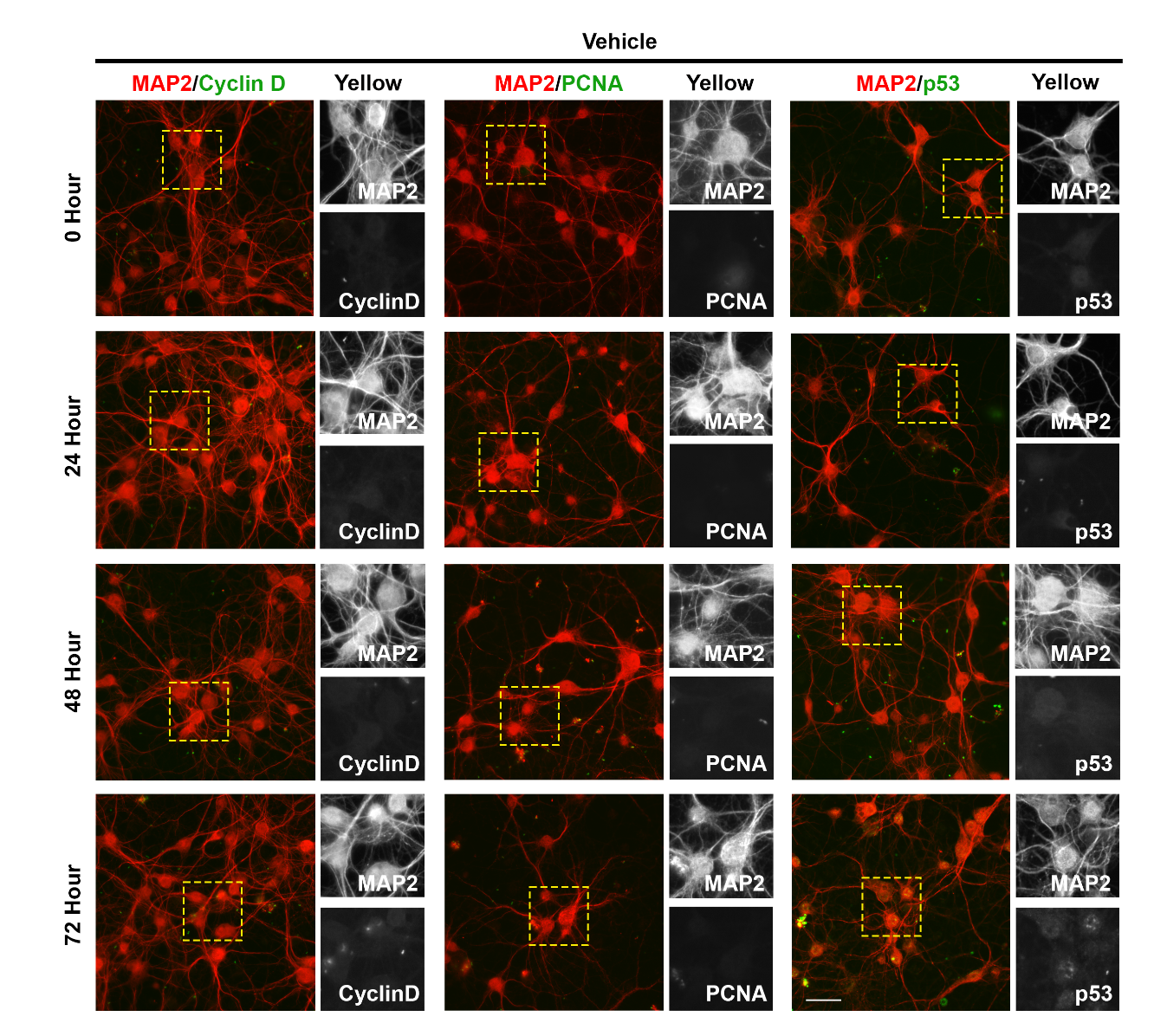


**Supplementary Figure 3. Baseline signals of cell cycle markers in vehicle-treated cortical neurons.** In complement to Figure 2F, representative immunofluorescent staining images of primary neurons subjected to exposure to vehicle for different time course in the CIE treatment paradigm (N=20, scale bar: 100μm).


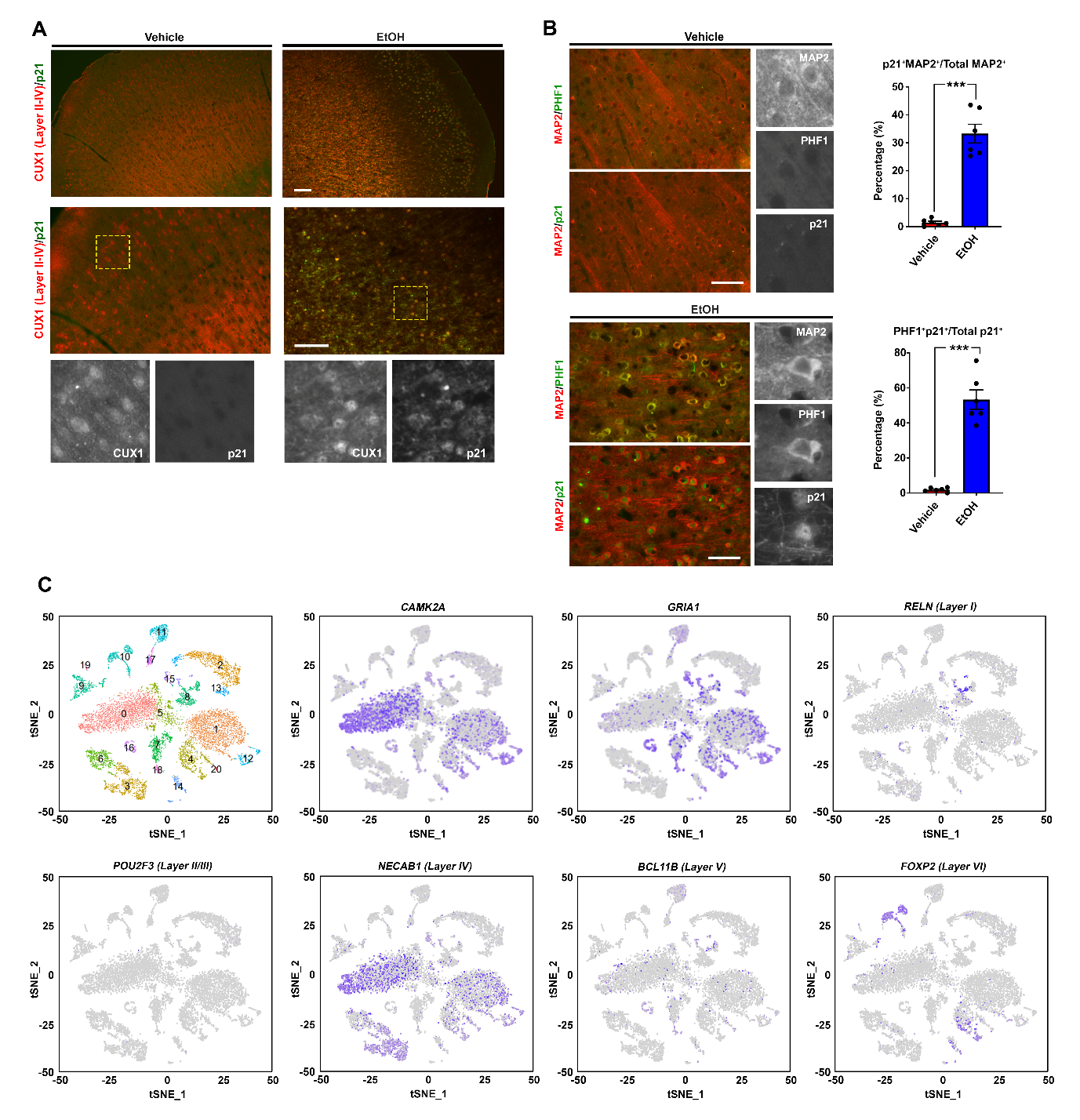


**Supplementary Figure 4. Upper cortical layer identities of senescent neurons.** (A) Representative immunohistochemistry images illustrating p21+ senescent neurons were predominantly overlapped with CUX1 signals, a classic marker for Layer II-IV neurons (N=5, Scale bar=200μm). (B) Representative immunohistochemistry images illustrating most p21+ senescent neurons were also positive for PHF1 phospho-Tau signals. Quantifications were shown (N=6, ***P<0.0001, two-tailed unpaired t-test). (C) T-distributed stochastic neighbour embedding (t-SNE) plot of all nuclei extracted from the dataset, which was then segregated and coloured as 20 distinct clusters of brain cells, based on their transcriptome features. Specific cortical layer markers were used to identify neurons from different layers. Clusters 0 and 5 excitatory neurons were mainly located at Layer IV.


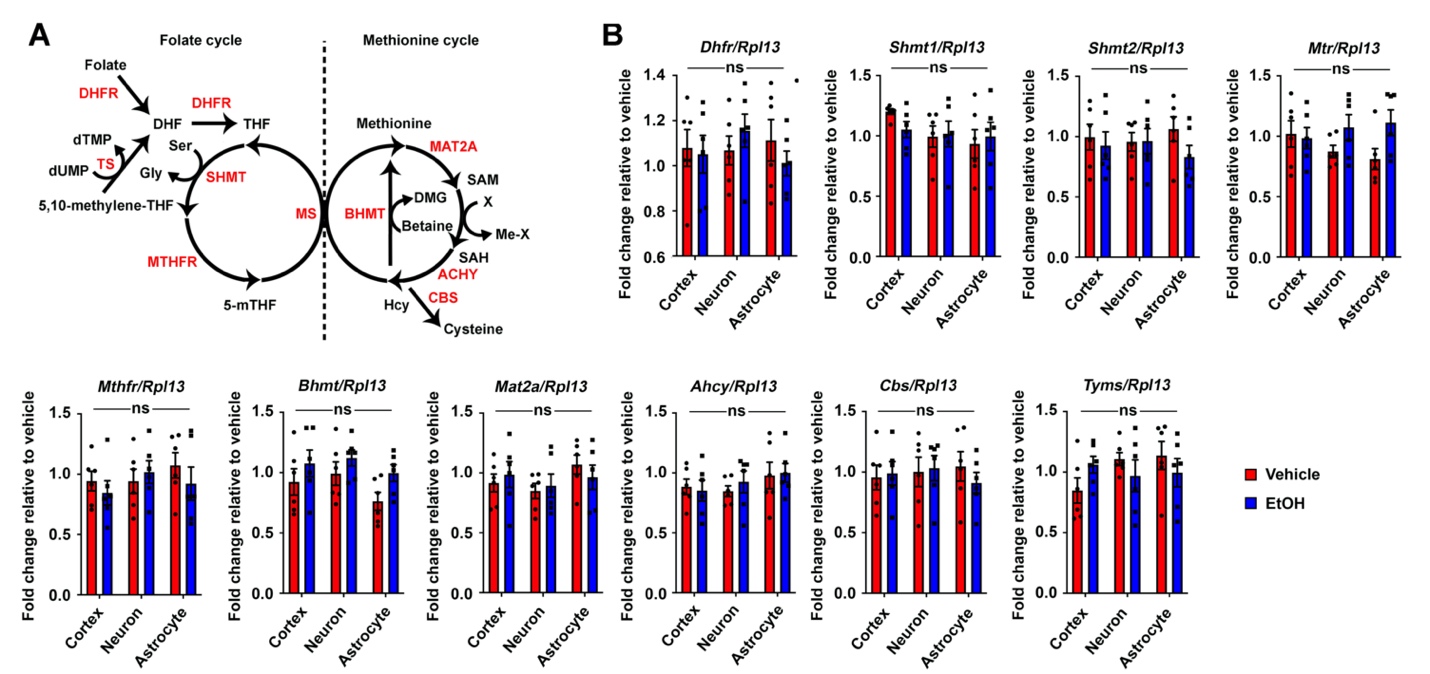


**Supplementary Figure 5. Ethanol exposure has no effect on expression of enzymes in the folate-methionine network.** (A) Schematic diagram showing the genes of key enzymes involved in regulating the folate-methionine network, (B) their expression levels were not affected upon chronic ethanol exposure (N=8, ns=non-significant, two-tailed unpaired t-test).

**
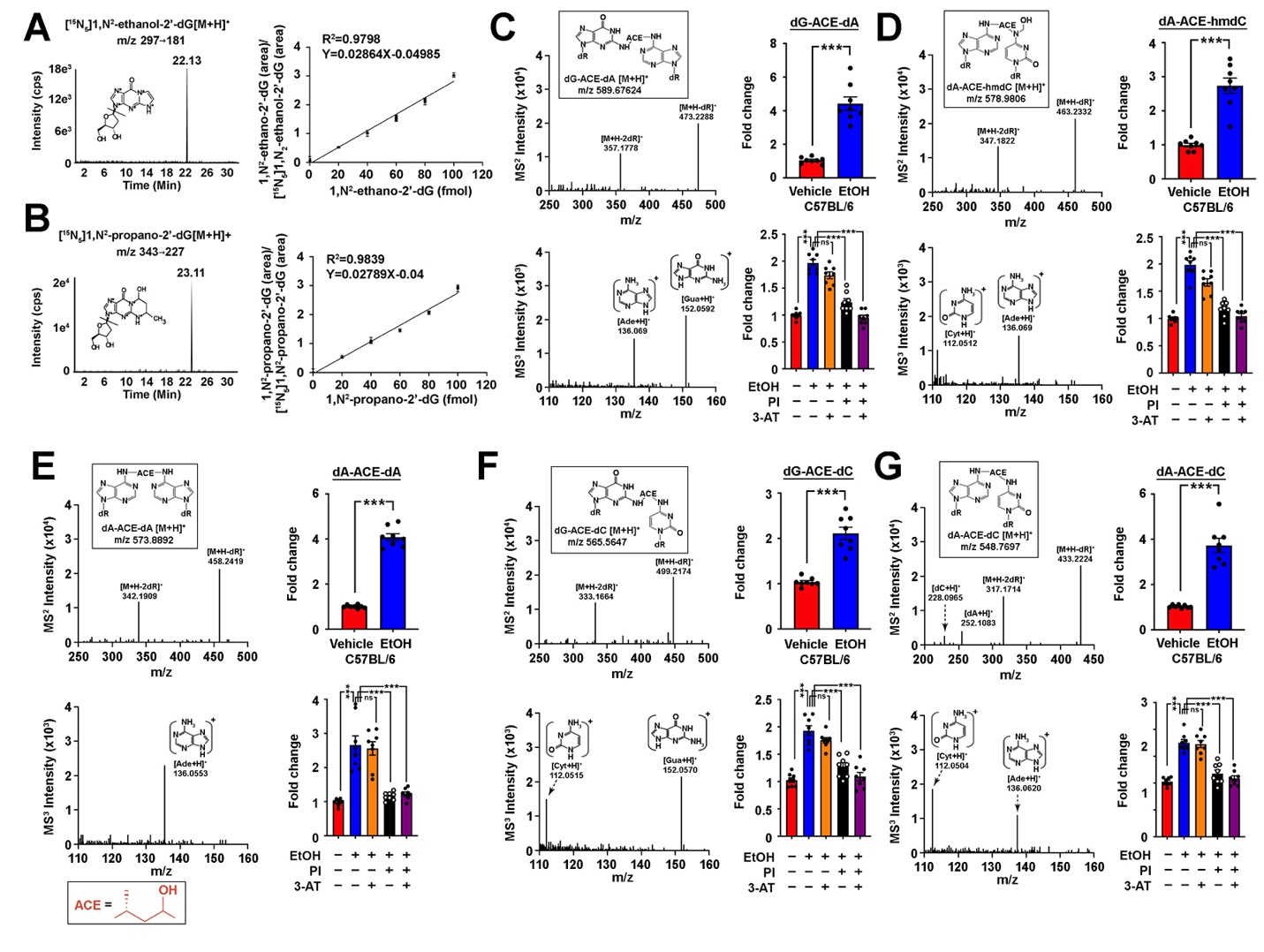
**

**Supplementary Figure 6. HPLC/ESI/MS-MS detection of DNA adducts and crosslinks.** (A) Representative ion chromatograms isotopic internal standards [^15^N_5_]-1,N^2^-ethano-2’-dG and (B) [^15^N_5_]-1,N^2^-propano-2’-dG, and the calibration curves were shown. Calibration curves were obtained by plotting the relative area ratios of unlabelled adducts to the isotopically ^15^N_5_-labelled adduct versus increasing amounts of [^15^N_5_]-1,N^2^-ethano-2’-dG and [^15^N_5_]-1,N^2^-propano-2’-dG (N=6). Conditions were as described in the Experimental Procedures section. (C-G) Product ion spectra of acetaldehyde-induced DNA crosslinks measured as dinucleosides. Quantification of their relative abundances in brain cortical tissues (N=8, ***P<0.0001, two-tailed unpaired t-test) and primary cortical neuron subjected to the CIE and drug co-treatment for 72 hours (10μM PI and 20μM 3-AT) (N=8, ***P<0.0001, one-way ANOVA).


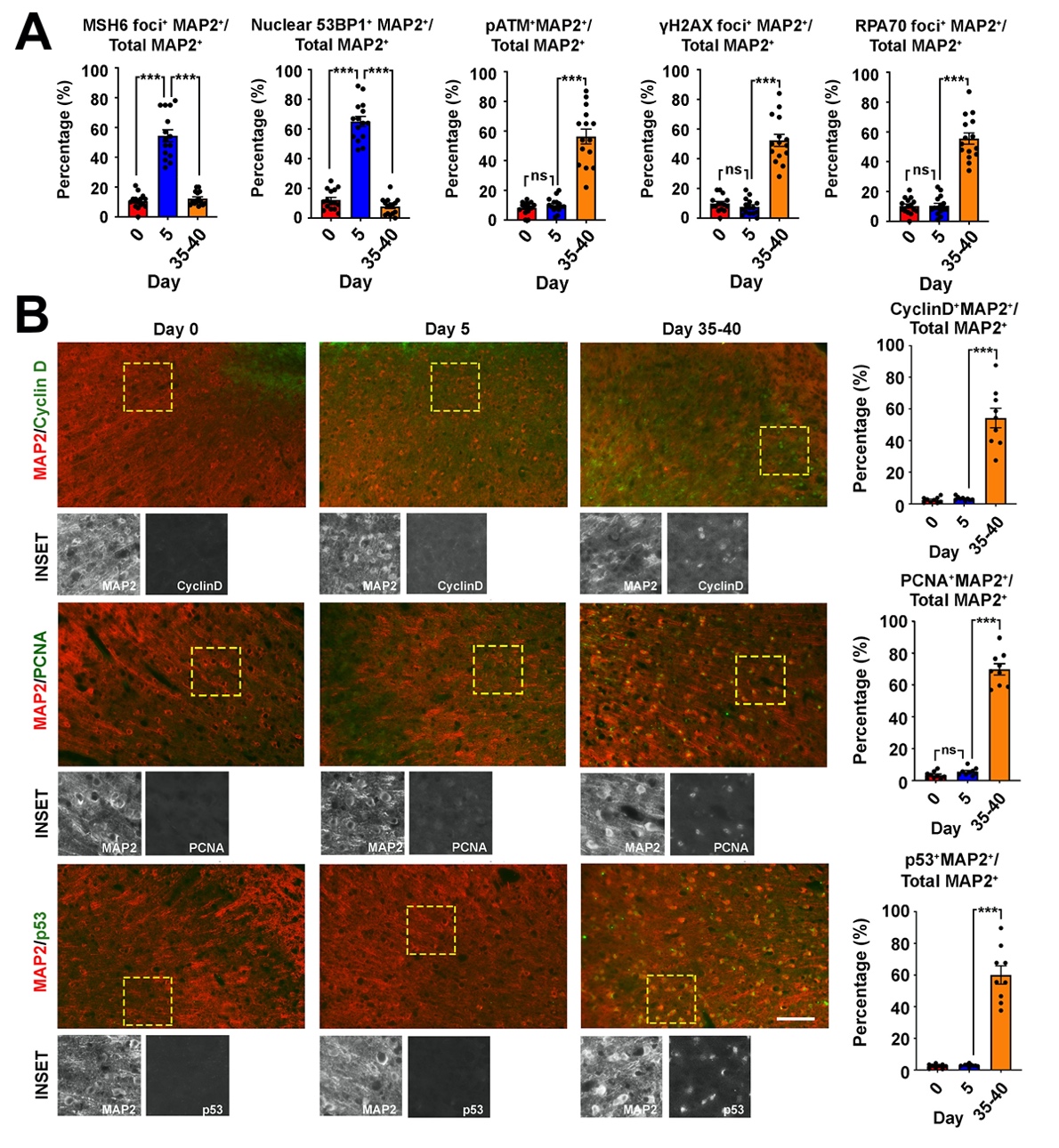


**Supplementary Figure 7.** **Additional data on illustrating the relationship between the length of chronic alcohol drinking period and neuronal cell cycle re-entry responses** (A) Quantification of the relative abundance of neurons positive for different markers of repair pathways as shown in Figure 6A (N=15, ***P<0.0001, ns=non-significant, one-way ANOVA). (B) Representative immunohistochemistry images of neuronal cell cycle markers emerged at different time points of the 2BC-DID paradigm. Quantifications were shown (N=9, ***P<0.0001, ns=non-significant, one-way ANOVA, scale bar: 200μm).


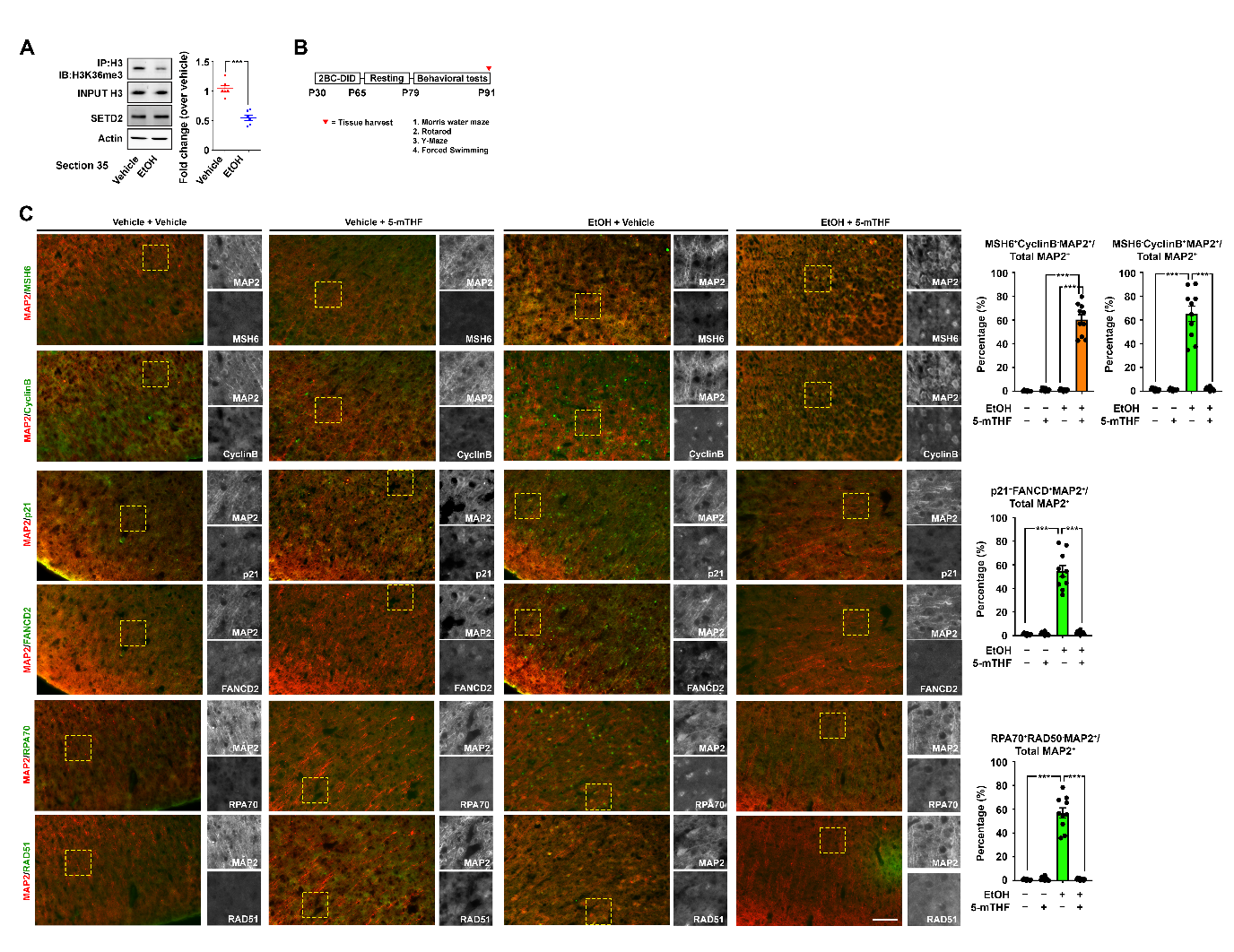


**Supplementary Figure 8. Additional information on 5-methyl tetrahydrofolate (5-mTHF) supplementation supports mismatch repair signalling, prevents neuronal cell senescence and brain function decline upon chronic ethanol exposure.** (A) Representative Western blots showing that 2BC-DID treatment for 35 days resulted in reduced activities of SETD2, reflected by reduced levels of H3K36me3. Quantification of the relative band intensities between H3K36me3 over total histone H3 levels were shown (N=6, ***P<0.0001, Two tailed unpaired t-test). (B) Schematic diagram showing the mouse treatment scheme. By three months of age, mice were subjected to the 2BC-DID paradigm with or without intranasal 5-mTHF supplementation every night, followed by 14 days of alcohol abstained period before undergoing through behavioural testing to evaluate if neuronal *Aldh1* knockout would affect the lasting behaviour and cellular changes. (C) Representative brain section images illustrating the effect of 5-mTHF supplementation on the pathways choice of repair, cell cycle and senescence markers. Quantification of neurons positive for different markers were shown (N=10, ***P<0.0001, One-way ANOVA, Scale bar=200μm).


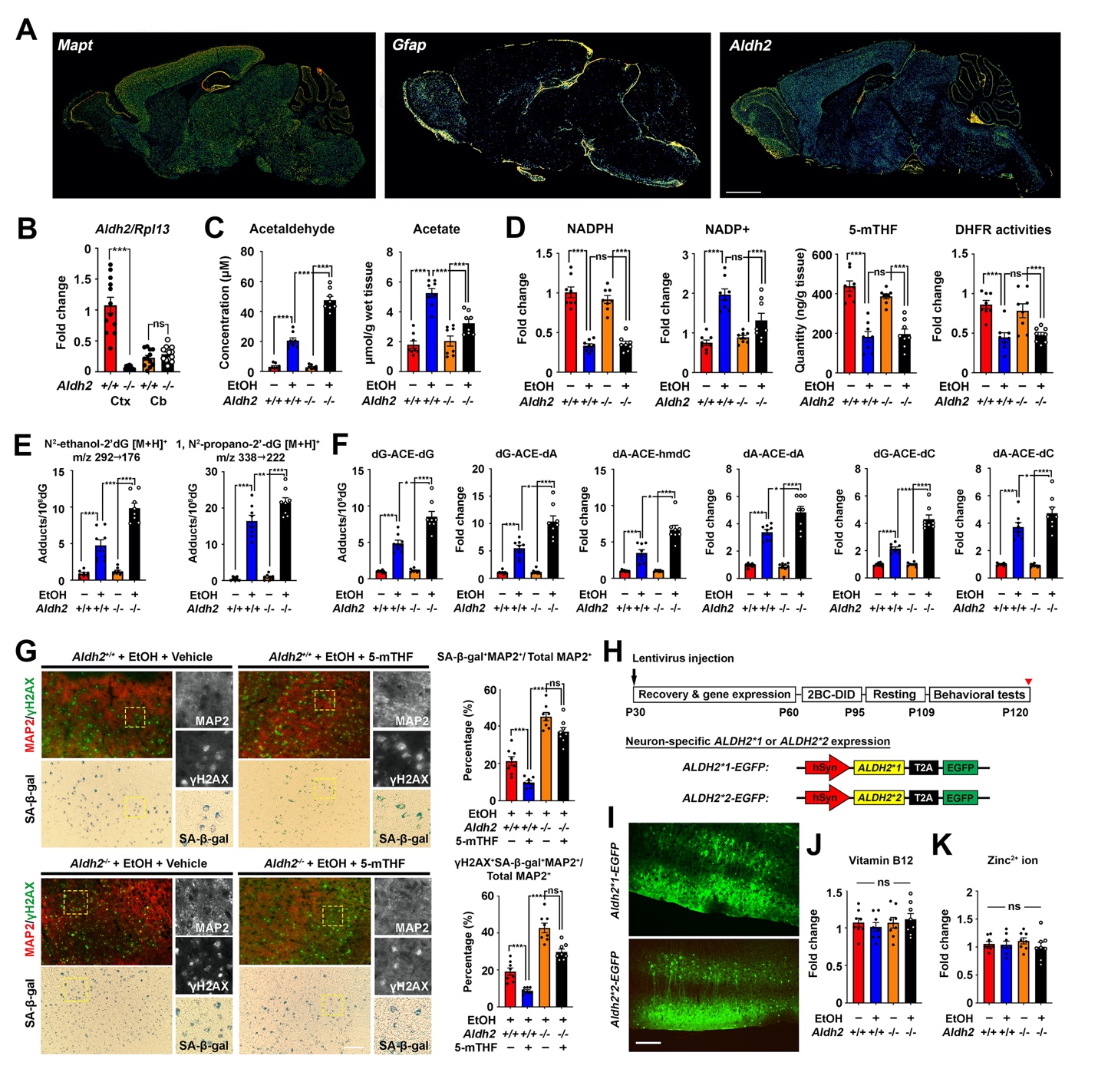


**Supplementary Figure 9. Additional data on how *ALDH2* loss-of-function mutation exacerbates neuronal senescence but could be alleviated by a metabolic drug-nutrient dyad strategy.** (A) Representative in situ hybridization showing the relative abundance of *Aldh2* mRNA in different brain regions and cell types of a mouse brain, adopted from the Allen Brain Mouse Atlas. (B) Quantification of relative abundance of *Aldh2* mRNA levels in the cortex (Ctx) versus cerebellum (Cb) regions of the *Aldh2^+/+^* and *Aldh2^-/-^* mice (N=12, ***P<0.0001, ns= non-significant, two-tailed unpaired t-test). (C) Quantification of brain cortex acetaldehyde and acetate contents in *Aldh2^+/+^* and *Aldh2^-/-^* mice (N=8, ***P<0.0001, one-way ANOVA). (D) Quantifications of the relative levels of NADPH, NADP+ and 5-mTHF levels as well as the activities of DHFR in cortex tissues of the *Aldh2^+/+^* and *Aldh2^-/-^* mice after completing the 2BC-DID paradigm (N=8, ***P<0.0001, ns=non-significance, one-way ANOVA). (E-F) Quantification of the relative abundance of various forms (E) DNA adducts and (F) crosslinks in the brain cortical tissues harvested from *Aldh2^+/+^* and *Aldh2^-/-^* mice subjected to the 2BC-DID paradigm (N=8, ***P<0.0001, *P<0.01, one way ANOVA). (G) Representative images illustrating the effect of 5-mTHF supplementation on the accumulation of γH2AX- and SA-β-gal-double positive neurons in the prefrontal cortex region of *Aldh2^+/+^ and Aldh2^-/-^* mice induced after the entire 2BC-DID treatment paradigm. Quantifications were shown (N=8, ***P<0.0001, ns=non-significant, one-way ANOVA, scale bar: 200μm). (H) Schematic diagram indicating the relative timing of stereotaxic injection of lentivirus carrying human *ALDH2*1* or *ALDH2*2* construct into the *Aldh2^-/-^* mice, followed by the 2BC-DID paradigm, intranasal co-administration of supplements (100 ng/day 5-mTHF ± 25 µg/day Aldh1 in a total of 10 µl) and behavioural analyses. At the bottom were the design of the *ALDH2*1* and *ALDH2*2* constructs that drove ectopic expression specific in neurons via the control of synapsin-1 promoter. (I) Representative images showing the expression efficiencies of *ALDH2*1* and *ALDH2*2* constructs in the prefrontal cortices of the brain at 30 days after the initial injection (N=8, scale bar=100μm). (J-K) Quantification of brain cortex (J) vitamin B12 and (K) Zinc 2+ ion levels in *Aldh2^+/+^* and *Aldh2^-/-^* mice (N=8, ns=non-significant, one-way ANOVA).


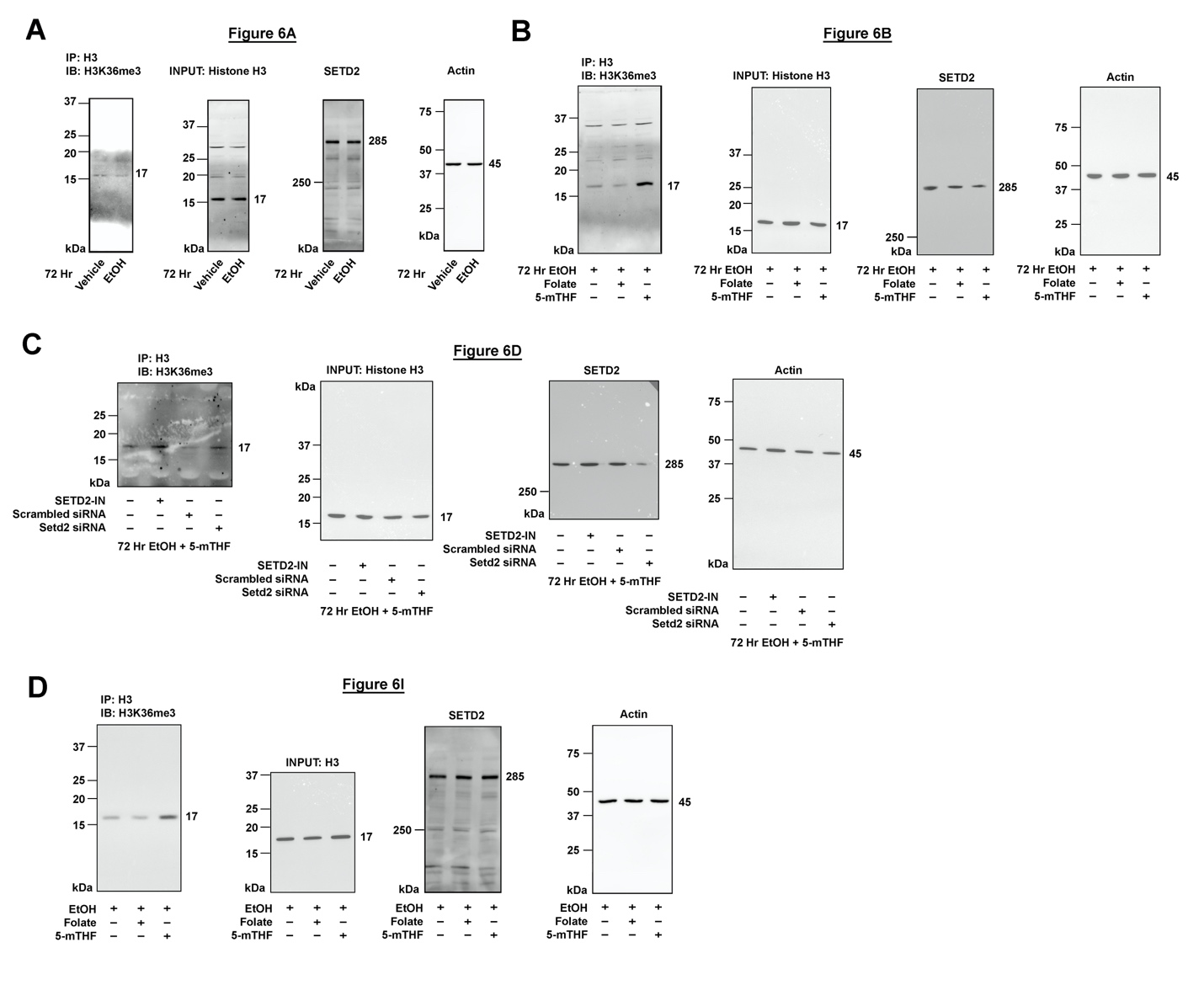


**Supplementary Figure 8. Full scans of representative immunoblot shown in different figures.**

**References**

Batiuk, M. Y., de Vin, F., Duque, S. I., Li, C., Saito, T., Saido, T., . . . Holt, M. G. (2017). An immunoaffinity-based method for isolating ultrapure adult astrocytes based on ATP1B2 targeting by the ACSA-2 antibody. *J Biol Chem, 292*(21), 8874-8891. doi:10.1074/jbc.M116.765313

Chow, H. M., Shi, M., Cheng, A., Gao, Y., Chen, G., Song, X., . . . Herrup, K. (2019). Age-related hyperinsulinemia leads to insulin resistance in neurons and cell-cycle-induced senescence. *Nat Neurosci, 22*(11), 1806-1819. doi:10.1038/s41593-019-0505-1

Chow, H. M., Sun, J. K., Hart, R. P., Cheng, K. K., Hung, C. H. L., Lau, T. M., & Kwan, K. M. (2021). Low-Density Lipoprotein Receptor-Related Protein 6 Cell Surface Availability Regulates Fuel Metabolism in Astrocytes. *Adv Sci (Weinh)*, e2004993. doi:10.1002/advs.202004993

Evangelou, K., & Gorgoulis, V. G. (2017). Sudan Black B, The Specific Histochemical Stain for Lipofuscin: A Novel Method to Detect Senescent Cells. *Methods Mol Biol, 1534*, 111-119. doi:10.1007/978-1-4939-6670-7_10

Eysseric, H., Gonthier, B., Soubeyran, A., Bessard, G., Saxod, R., & Barret, L. (1997). There is not simple method to maintain a constant ethanol concentration in long-term cell culture: keys to a solution applied to the survey of astrocytic ethanol absorption. *Alcohol, 14*(2), 111-115. doi:10.1016/s0741-8329(96)00112-7

Foo, L. C., Allen, N. J., Bushong, E. A., Ventura, P. B., Chung, W. S., Zhou, L., . . . Barres, B. A. (2011). Development of a method for the purification and culture of rodent astrocytes. *Neuron, 71*(5), 799-811. doi:10.1016/j.neuron.2011.07.022

Green, C. R., Wallace, M., Divakaruni, A. S., Phillips, S. A., Murphy, A. N., Ciaraldi, T. P., & Metallo, C. M. (2016). Branched-chain amino acid catabolism fuels adipocyte differentiation and lipogenesis. *Nat Chem Biol, 12*(1), 15-21. doi:10.1038/nchembio.1961

Hu, C. W., Chang, Y. J., Cooke, M. S., & Chao, M. R. (2019). DNA Crosslinkomics: A Tool for the Comprehensive Assessment of Interstrand Crosslinks Using High Resolution Mass Spectrometry. *Anal Chem, 91*(23), 15193-15203. doi:10.1021/acs.analchem.9b04068

Hui, C. W., Zhang, Y., & Herrup, K. (2016). Non-Neuronal Cells Are Required to Mediate the Effects of Neuroinflammation: Results from a Neuron-Enriched Culture System. *PLoS One, 11*(1), e0147134. doi:10.1371/journal.pone.0147134

Rydbirk, R., Folke, J., Winge, K., Aznar, S., Pakkenberg, B., & Brudek, T. (2016). Assessment of brain reference genes for RT-qPCR studies in neurodegenerative diseases. *Sci Rep, 6*, 37116. doi:10.1038/srep37116

Soga, T., Baran, R., Suematsu, M., Ueno, Y., Ikeda, S., Sakurakawa, T., . . . Tomita, M. (2006). Differential metabolomics reveals ophthalmic acid as an oxidative stress biomarker indicating hepatic glutathione consumption. *J Biol Chem, 281*(24), 16768-16776. doi:10.1074/jbc.M601876200

Soga, T., Igarashi, K., Ito, C., Mizobuchi, K., Zimmermann, H. P., & Tomita, M. (2009). Metabolomic profiling of anionic metabolites by capillary electrophoresis mass spectrometry. *Anal Chem, 81*(15), 6165-6174. doi:10.1021/ac900675k

Wang, L., Hirayasu, K., Ishizawa, M., & Kobayashi, Y. (1994). Purification of genomic DNA from human whole blood by isopropanol-fractionation with concentrated Nal and SDS. *Nucleic Acids Res, 22*(9), 1774-1775. doi:10.1093/nar/22.9.1774

Zhang, Y., Sloan, S. A., Clarke, L. E., Caneda, C., Plaza, C. A., Blumenthal, P. D., . . . Barres, B. A. (2016). Purification and Characterization of Progenitor and Mature Human Astrocytes Reveals Transcriptional and Functional Differences with Mouse. *Neuron, 89*(1), 37-53. doi:10.1016/j.neuron.2015.11.013
