# Supplementary figures and images for: Chronic alcohol metabolism results in DNA repair infidelity and cell cycle‐induced senescence in neurons
Source: Aging Cell. 2023 Jan 23;22(2):e13772. doi: 10.1111/acel.13772 (PMC9924945; doi:10.1111/acel.13772)

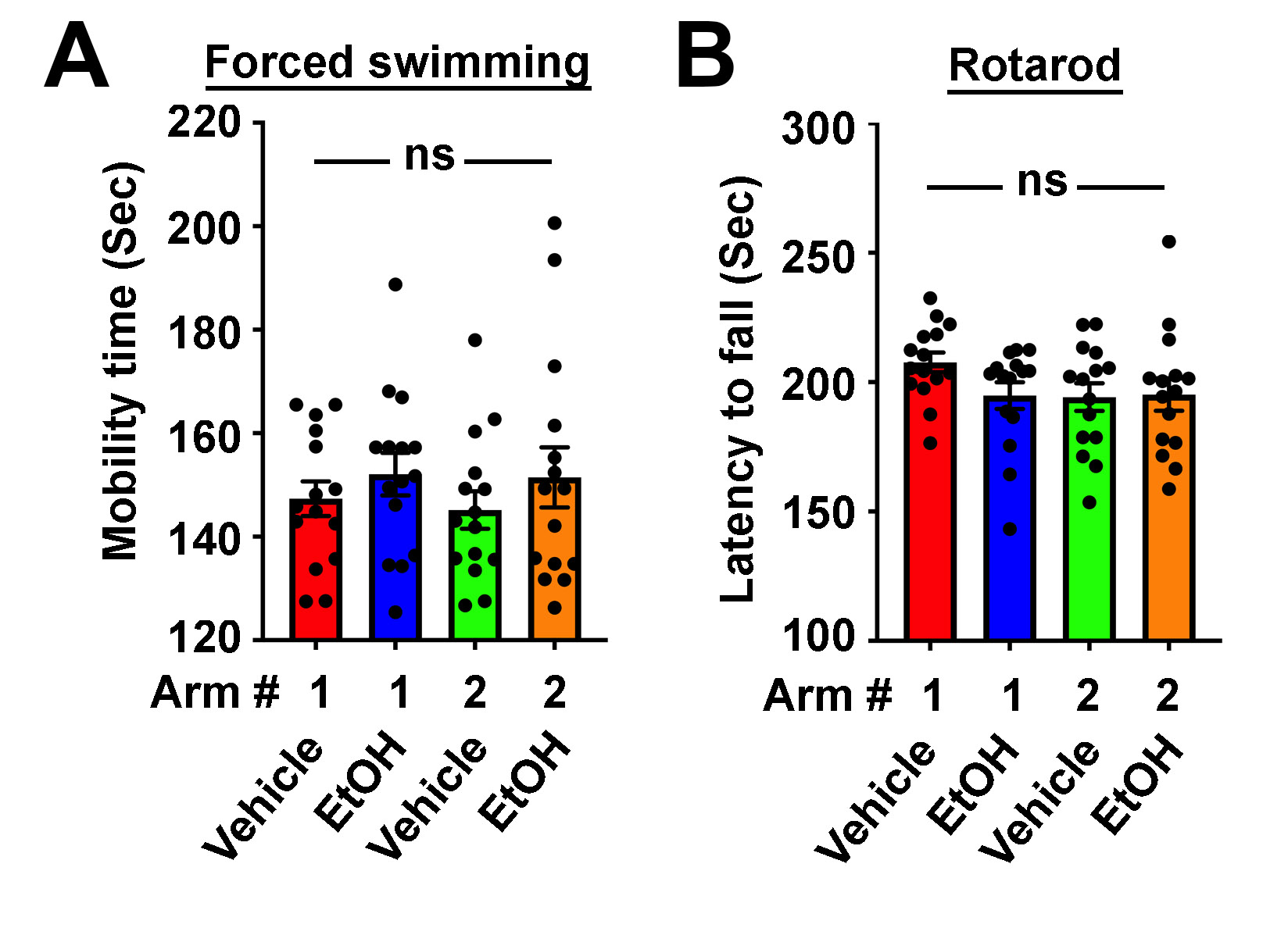

Supplement: Supplementary file 8 — FigureS1 [file ACEL-22-e13772-s008.jpg]

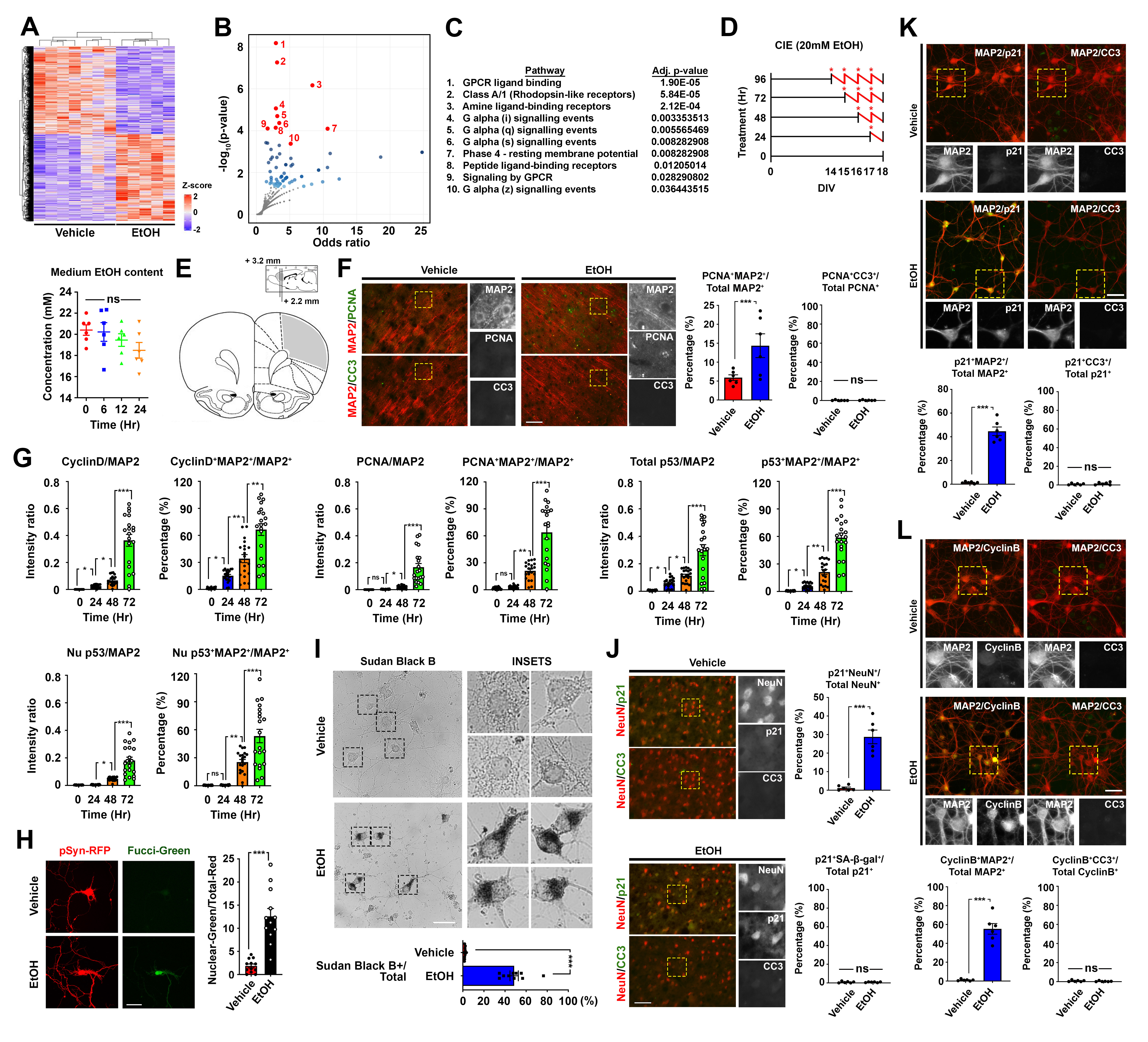

Supplement: Supplementary file 9 — FigureS2 [file ACEL-22-e13772-s003.jpg]

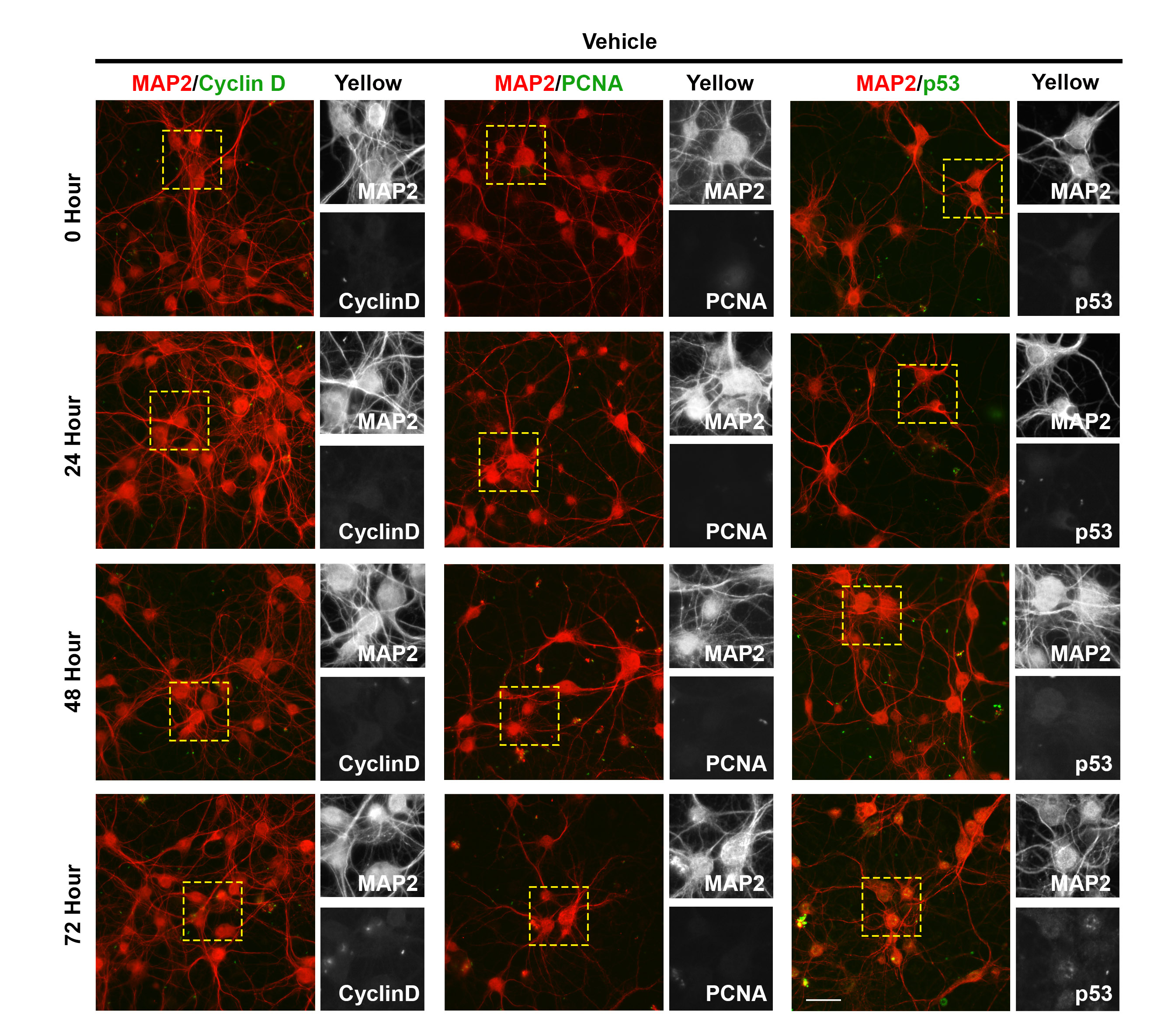

Supplement: Supplementary file 10 — FigureS3 [file ACEL-22-e13772-s013.jpg]

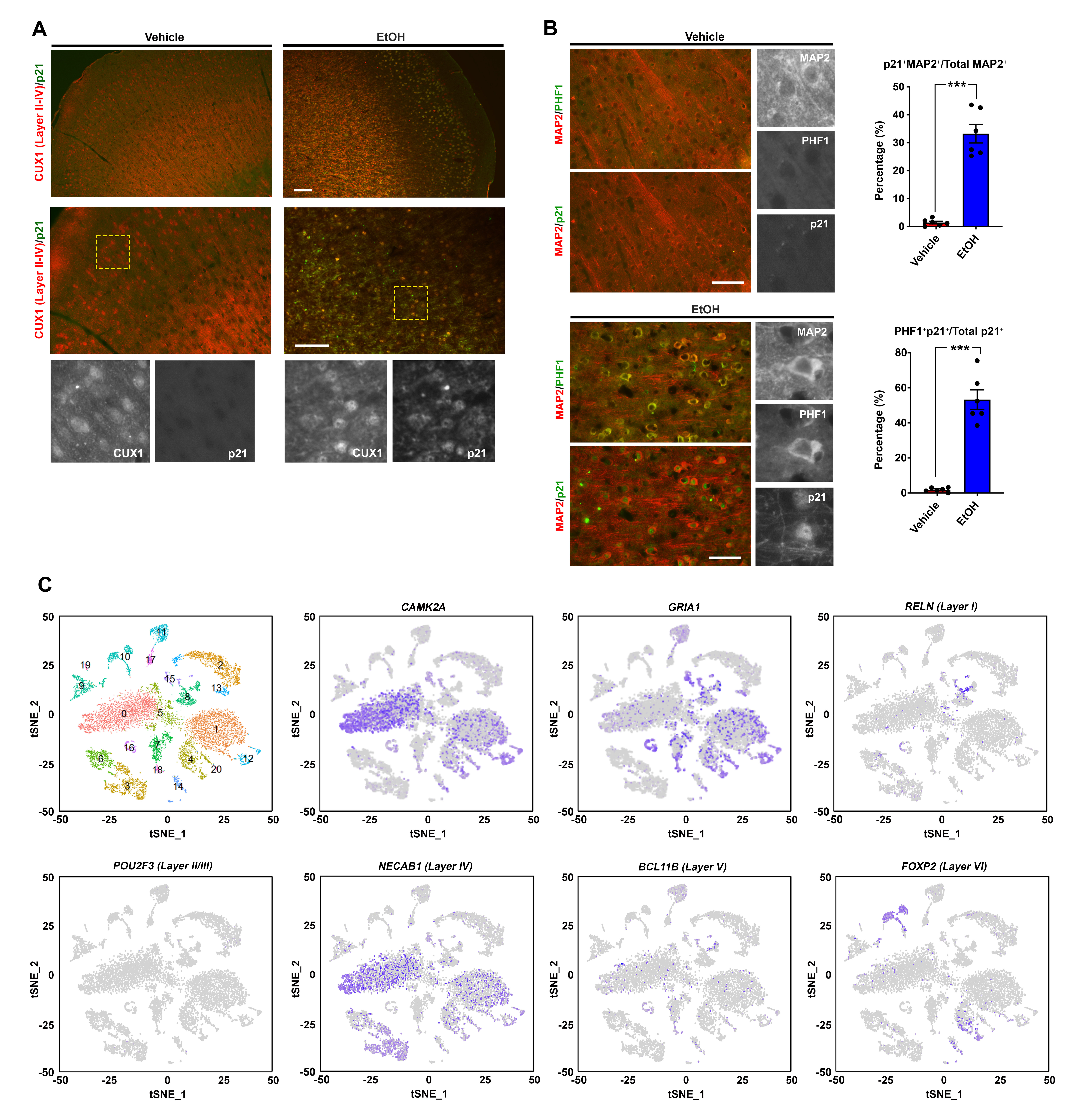

Supplement: Supplementary file 11 — FigureS4 [file ACEL-22-e13772-s004.jpg]

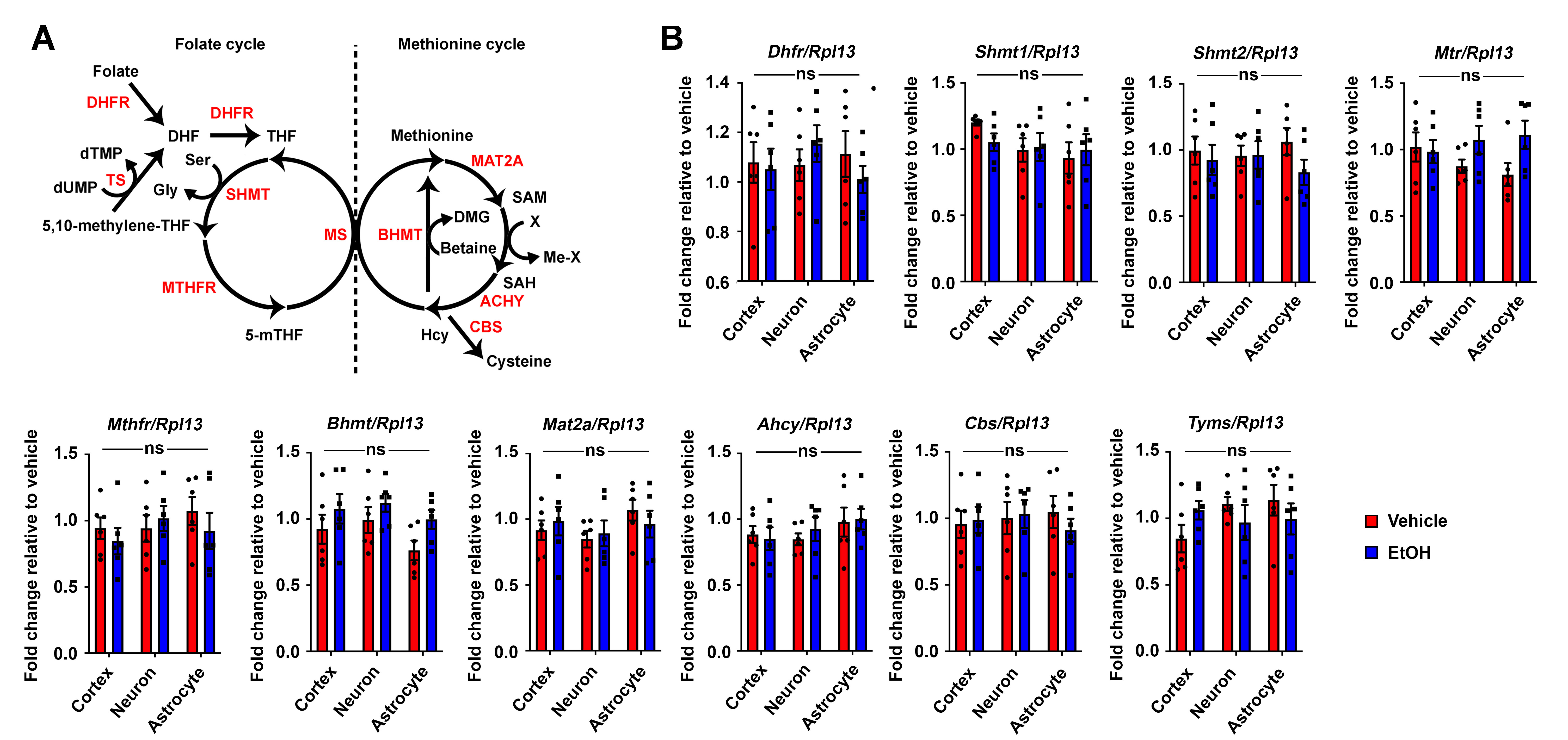

Supplement: Supplementary file 12 — FigureS5 [file ACEL-22-e13772-s009.jpg]

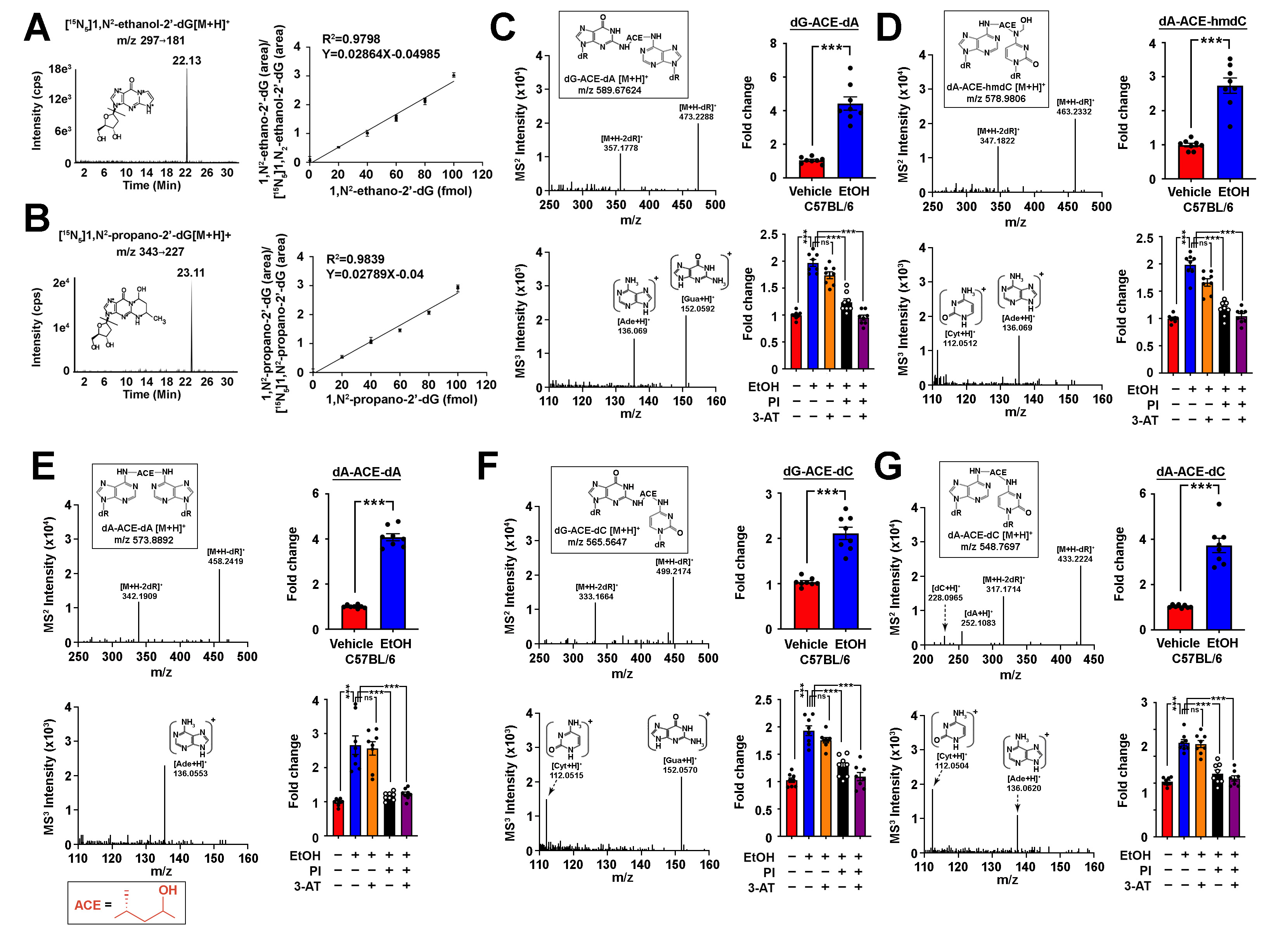

Supplement: Supplementary file 13 — FigureS6 [file ACEL-22-e13772-s011.jpg]

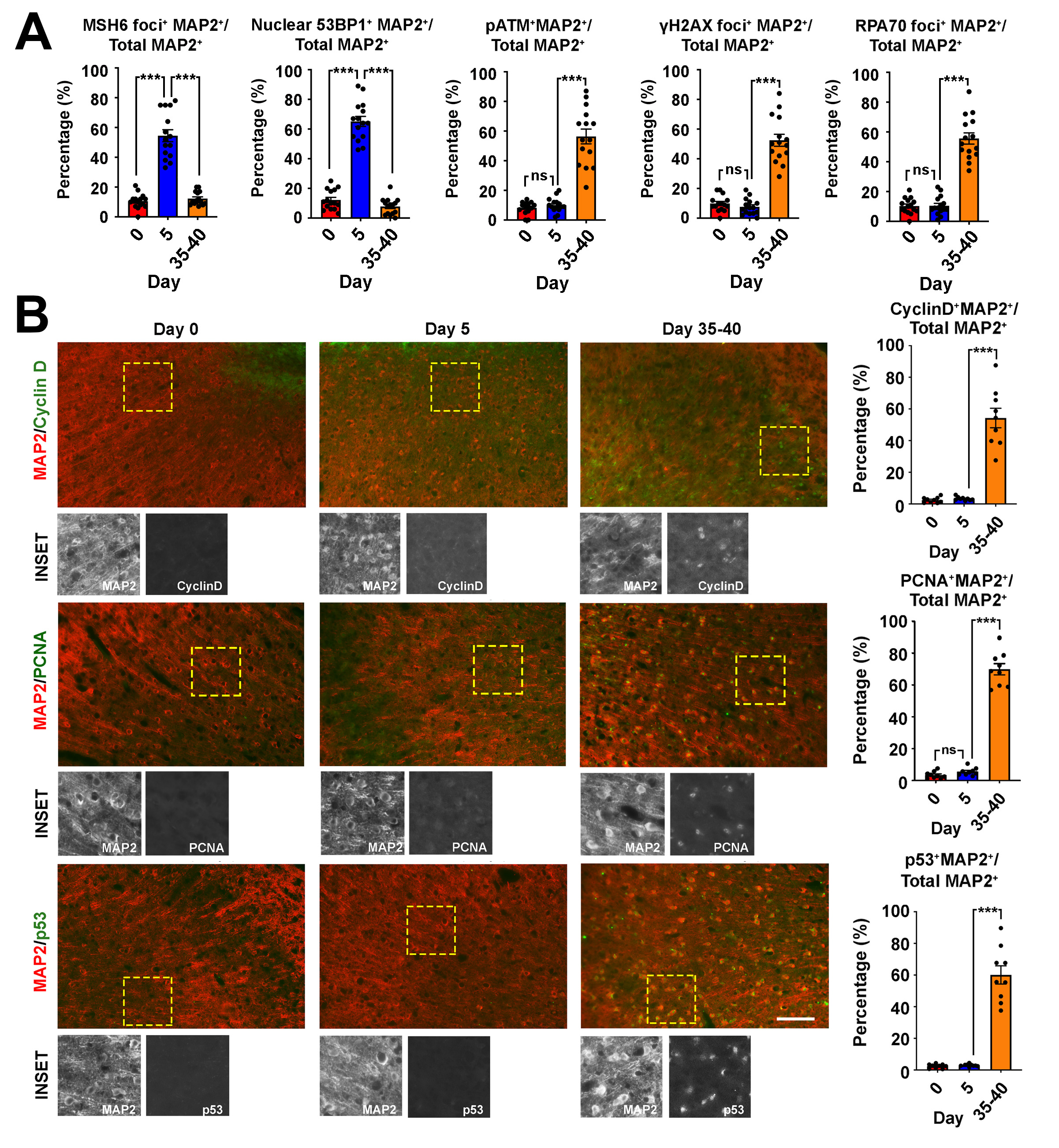

Supplement: Supplementary file 14 — FigureS7 [file ACEL-22-e13772-s002.jpg]

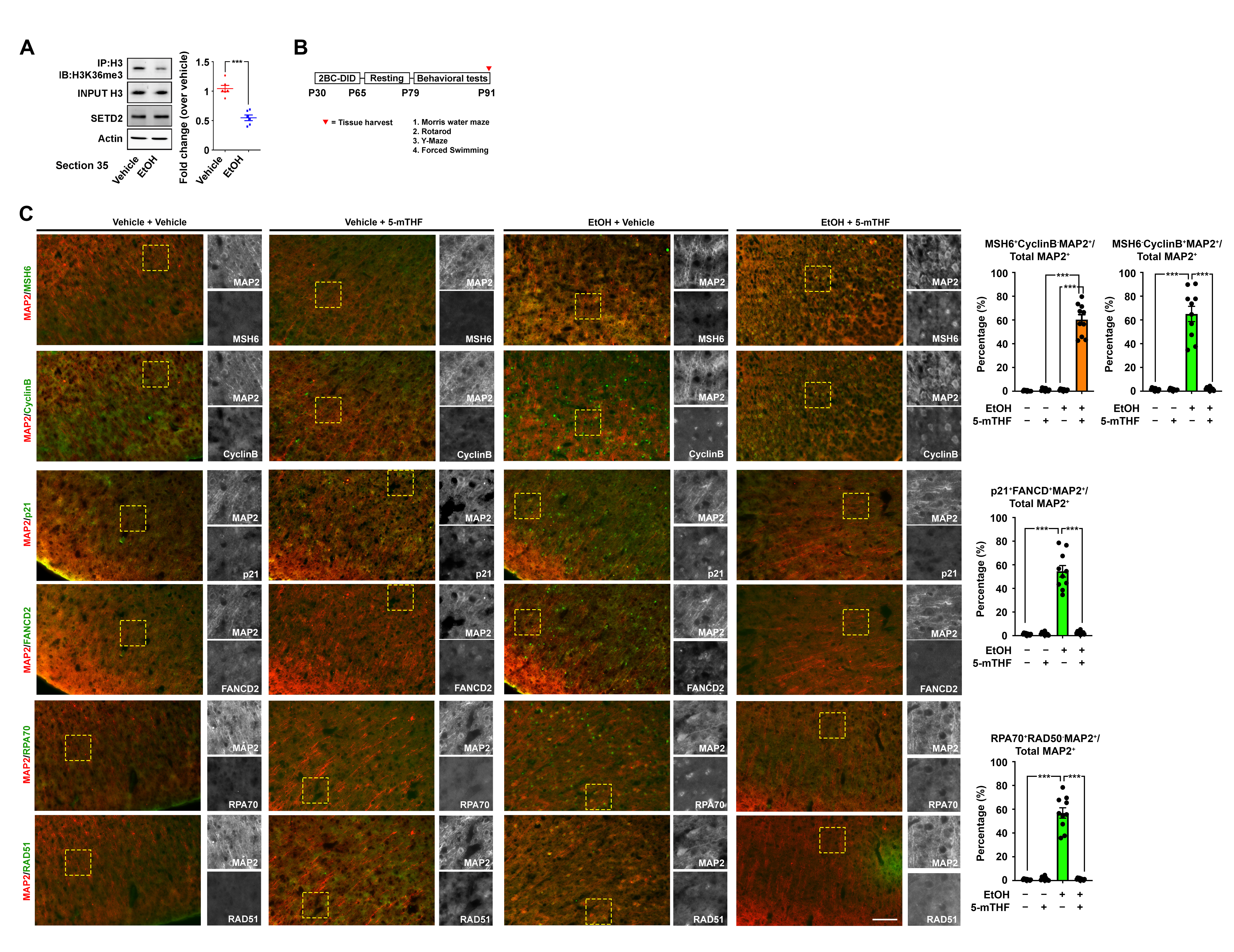

Supplement: Supplementary file 15 — FigureS8 [file ACEL-22-e13772-s016.jpg]

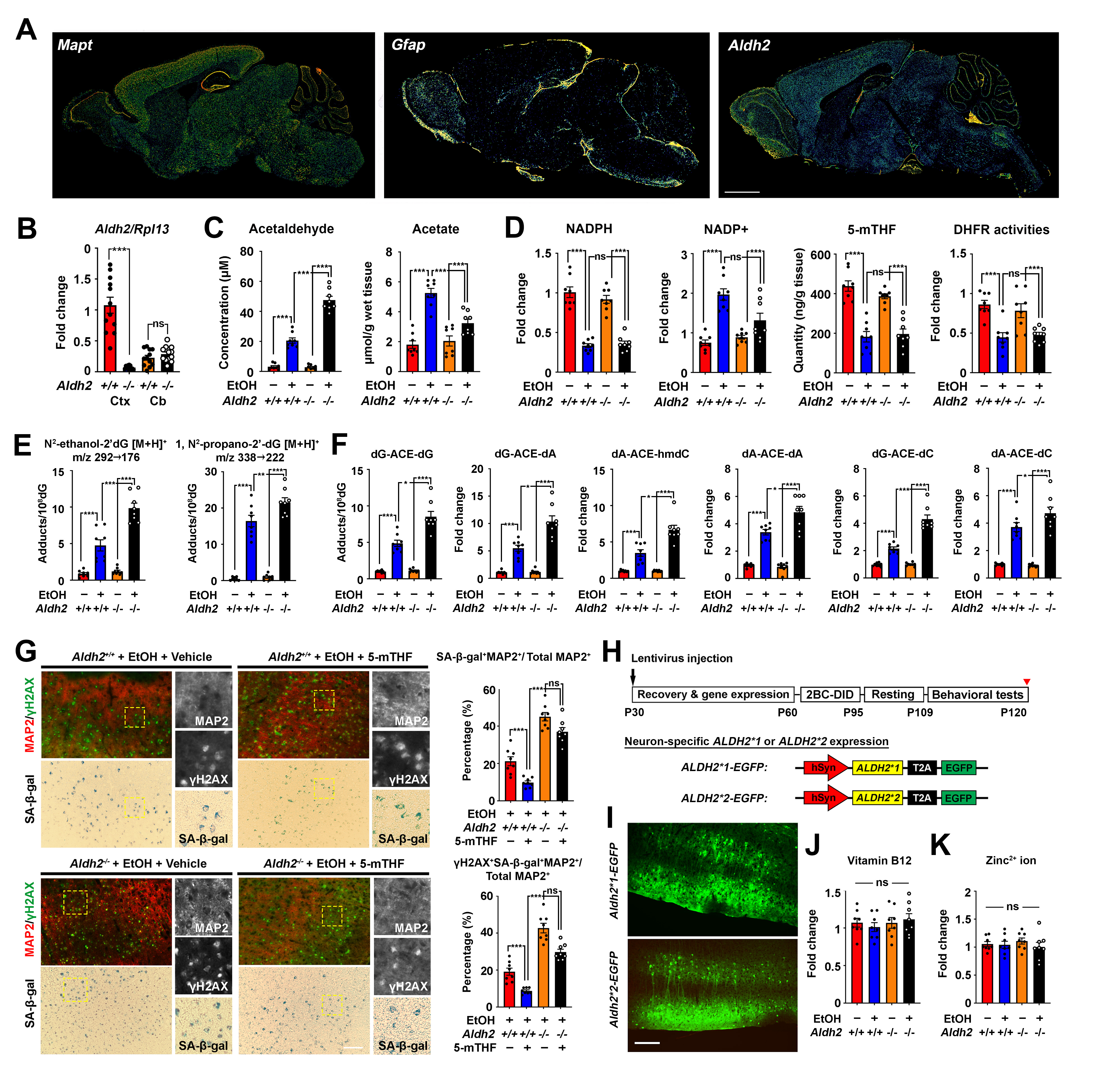

Supplement: Supplementary file 16 — FigureS9 [file ACEL-22-e13772-s010.jpg]

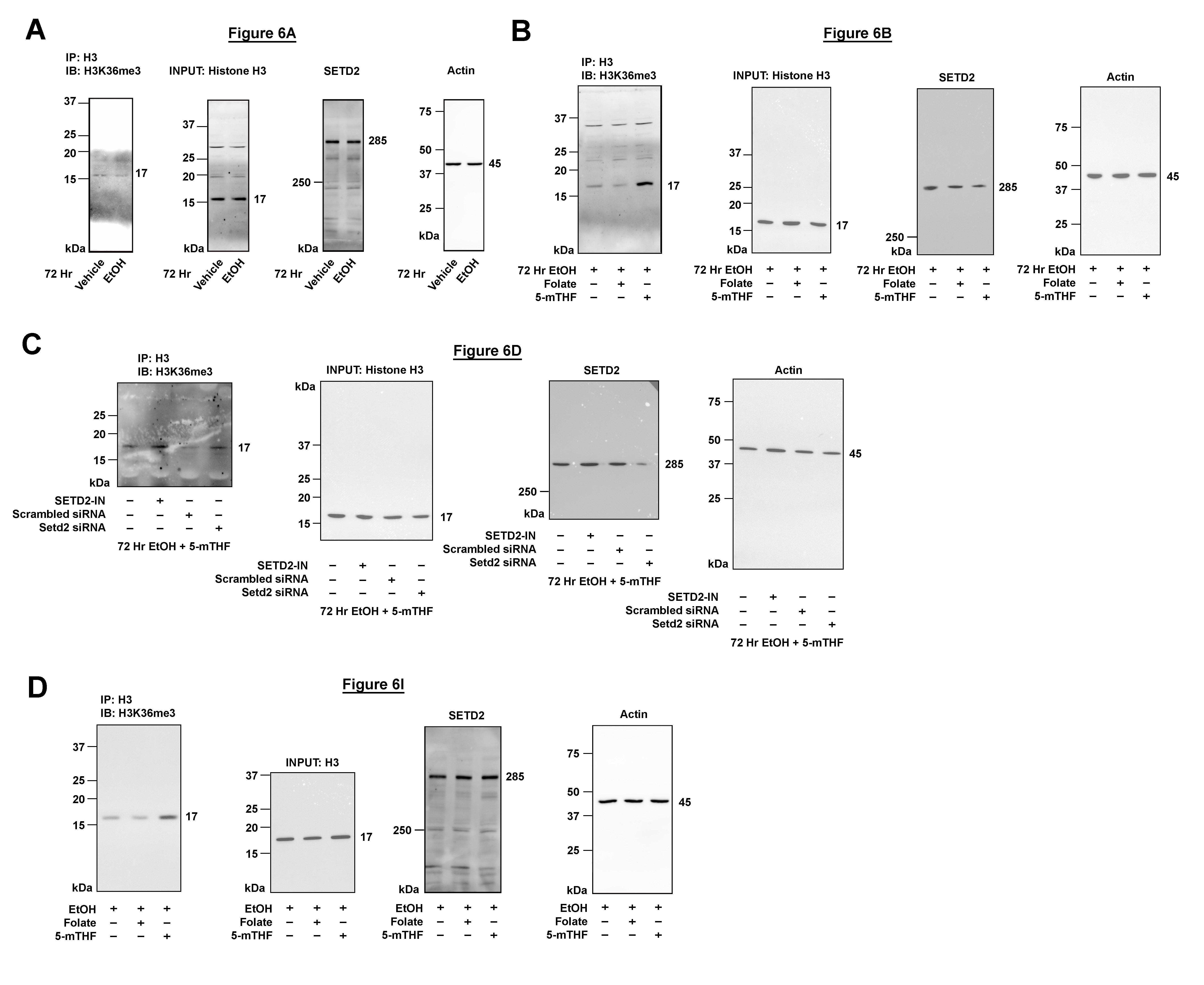

Supplement: Supplementary file 17 — FigureS10 [file ACEL-22-e13772-s014.jpg]
